# A Photo-responsive Transmembrane Anion Transporter Relay

**DOI:** 10.1021/jacs.2c02612

**Published:** 2022-06-02

**Authors:** Toby G. Johnson, Amir Sadeghi-Kelishadi, Matthew J. Langton

**Affiliations:** Chemistry Research Laboratory, University of Oxford, Mansfield Road, Oxford OX1 3TA, U.K.

## Abstract

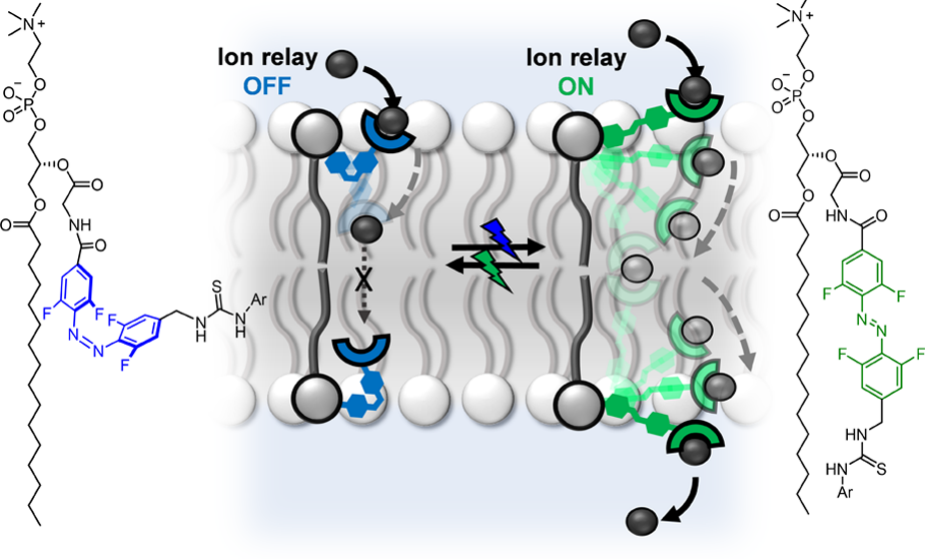

Ion transport across lipid membranes in biology is controlled by stimuli-responsive membrane channels and molecular machine ion pumps such as ATPases. Here, we report a synthetic molecular machine-like ion transport relay, in which transporters on opposite sides of a lipid bilayer membrane facilitate transport by passing ions between them. By incorporating a photo-responsive telescopic arm into the relay design, this process is reversibly controlled in response to irradiation with blue and green light. Transport occurs only in the extended state when the length of the arm is sufficient to pass the anion between transporters located on opposite sides of the membrane. In contrast, the contracted state of the telescopic arm is too short to mediate effective transport. The system acts as a stimuli-responsive ensemble of machine-like components, reminiscent of robotic arms in a factory assembly line, working cooperatively to mediate ion transport. This work points to new prospects for using lipid bilayer membranes as scaffolds for confining, orientating, and controlling the relative positions of molecular machines, thus enabling multiple components to work in concert and opening up new applications in biological contexts.

## Introduction

The transmembrane transport of ions is a fundamental process in biology, mediated by membrane channels and pumps.^1^ The activity of these transport proteins is regulated by external stimuli, including photons, small molecule binding, and membrane potential. The development of synthetic ion transporters such as self-assembled ion channels and mobile ion carriers has attracted significant interest, both as fundamental tools for investigating transmembrane ion transport and as potential therapeutics for diseases that arise from protein ion channel mis-regulation.^2−6^ Stimuli-responsive synthetic transporters, as artificial analogues of their protein counterparts, have potential utility in spatio-temporally targeted applications but remain comparatively rare.^7^ Synthetic channels^8−17^ and mobile ion carriers,^18−23^ which can be switched between inactive and active states, are highly desirable for achieving reversible, stimuli-responsive control over ion transport, but are particularly challenging to develop.

While the field of supramolecular ion transporters is dominated by channels and mobile carriers, new mechanisms of transport have emerged. These comprise Smith’s anion relay^24^ and more recent systems based on molecular machines^25^ including membrane-spanning [2]rotaxanes,^26,27^ molecular motors,^28−30^ and molecular swings.^31,32^ In these systems, the nano-mechanical motion of component parts of an individual molecular machine (e.g., a macrocycle moving back and forth along a rotaxane’s axle or pivoting of a molecular swing) is used to facilitate ion transport.

Confining stimuli-responsive artificial molecular machines within a lipid bilayer also offers the possibility of controlling the relative orientation and position of multiple individual components,^33^ analogous to anchoring molecular machines on a surface.^34^ This opens up the powerful concept of designing molecular machines whose function may be controlled in a stimuli-responsive manner, and where multiple components must work together in a cooperative fashion to carry out a task. However, to the best of our knowledge, this concept has not been explored using synthetic molecular machines within membranes.

**Figure 1:**
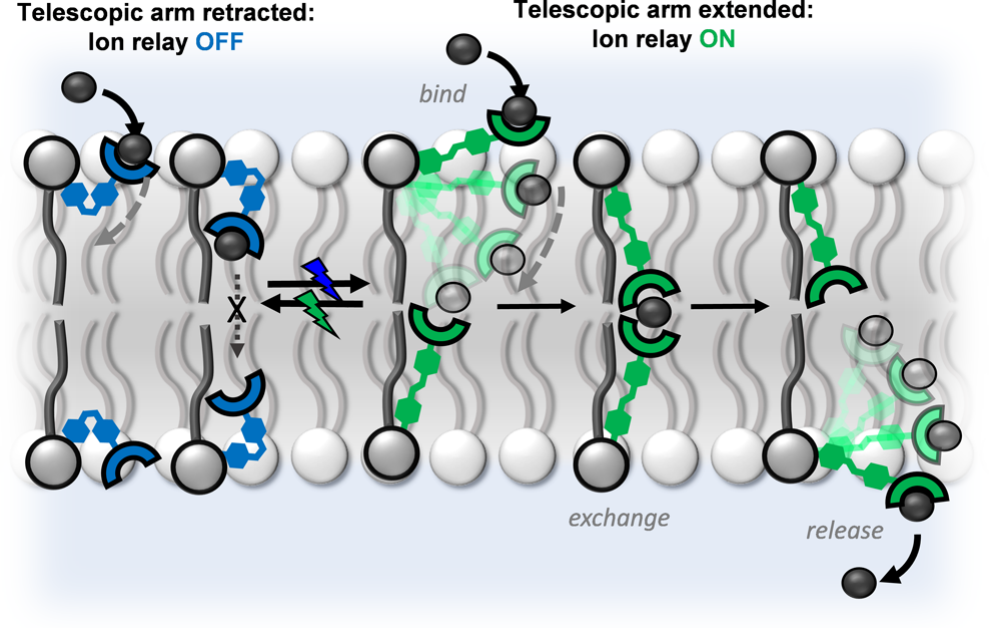
Schematic representation of a photo-responsive transmembrane relay. The relay is controlled by retracting or extending the telescopic arms of molecular machine-like transporters on either side of the membrane. When retracted, the anion-binding arms are incapable of exchanging ions between them (OFF state). Upon photo-irradiation, the transporter arm extends, facilitating ion relay between the transporters located on opposite sides of the membrane (ON state).

Herein, we demonstrate this concept using molecular anion receptors with length-controllable telescopic arms that are placed on either side of a lipid bilayer membrane, to form an anion transport relay (Figure 1). Photo-regulation of the length of these arms is used to control the relay process, such that transport occurs only in the extended state, when the length of the arms is sufficient to pass the anion between them. The ensemble of machine-like relay transporters is reminiscent of robotic arms in a factory assembly line, where multiple components working together are required.

## Results and Discussion

### Design and Synthesis

The design of the machine-like transport relay is shown in Figure 1. Two switchable anion receptors with telescopic arms that can be extended or contracted by photo-isomerization are embedded into opposite leaflets of the lipid bilayer membrane. In the extended state, a transporter in one leaflet of the membrane binds the anion at the water–bilayer interface and transports it to the center of the membrane. The telescopic arm is too short to allow release of the ion into the aqueous phase on the opposite side of the membrane but is capable of passing the anion to a transporter in the opposite leaflet, between the two thiourea binding sites. This in turn is able to transport the anion to the aqueous phase on the opposite side of the membrane. Conversely, when the telescopic arm is contracted, it is too short to facilitate anion exchange between each component of the relay, and transport is inhibited. Dynamic control over the extension and contraction of the transporter telescopic arm is achieved using light, thus providing a wholly new mechanism with which to regulate transmembrane transport, involving the cooperative action of two machine-like components on opposite sides of a membrane.

**Figure 2:**
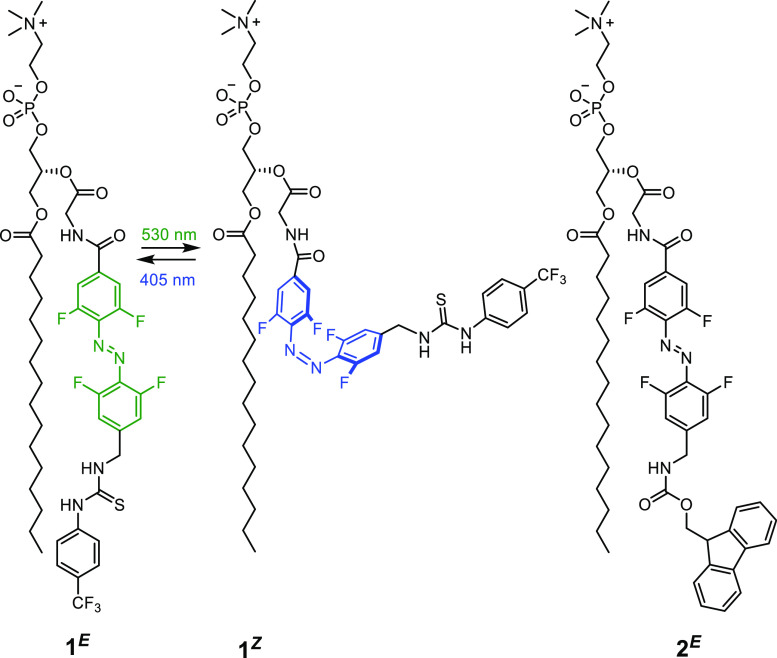
Photo-responsive relay transporters and control compound. Ion transport relay **1**, in the extended (*E*) and contracted (*Z*) states, and control compound **2**.

The lipid component of the transporter ensures excellent membrane uptake of the amphiphilic relay and anchors the transporter in one leaflet of the bilayer. Tetra-*o*-fluoro-azobenzenes^35^ were exploited as the photo-switchable moiety responsible for control over the length of the transporter. These photo-switches have long thermal half-lives (of the order of months) and well separated *E*(n−π*) and *Z*(n−π*) bands, which enables efficient photo-isomerization with bio-compatible wavelengths of visible light.^36−40^ An electron deficient aryl-thiourea was employed as a potent hydrogen bonding anion binding group to facilitate anion complexation and therefore entry into the membrane. The target photo-switchable relay transporter **1** (Figure 2) was accordingly prepared from the corresponding Fmoc-protected amine **2** by base-mediated deprotection and installation of the thiourea anion receptor. Intermediate **2** also acts as a control compound lacking the thiourea anion receptor and was prepared from the corresponding lysophospholipid and carboxylate-functionalized azobenzene. Full synthetic schemes and characterization are provided in the Supporting Information (Section S2).

We first examined the reversible visible-light-mediated photo-switching properties of relay **1**. The red-shifted *ortho*-fluoro-azobenzene derivative could be switched from *E* → *Z* with green light (530 nm) from an LED to achieve a photo-stationary state (PSS) distribution of 84% **1**^***Z***^, while isomerization in the reverse direction was achieved with blue light (405 nm, 95% **1**^***E***^ in the PSS, as determined by ^1^H NMR experiments in DMSO solution, Figure S44).

### Relay-Mediated Ion Transport

Evidence for ion transport by **1**^***E***^ via a relay mechanism was obtained from ion transport assays in large unilamellar vesicles (LUVs). Briefly, the relay transporters were pre-incorporated into the membrane of 1-palmitoyl-2-oleoyl-*sn*-glycero-3-phosphocholine vesicles (POPC LUVs), loaded with the pH-responsive fluorophore 8-hydroxypyrene-1,3,6-trisulfonate (HPTS) and buffered to pH 7.0 in NaCl solution. The ability of the transporter to dissipate a pH gradient generated by addition of a NaOH base pulse was determined by recording the change in the HPTS emission.^41^

**Figure 3:**
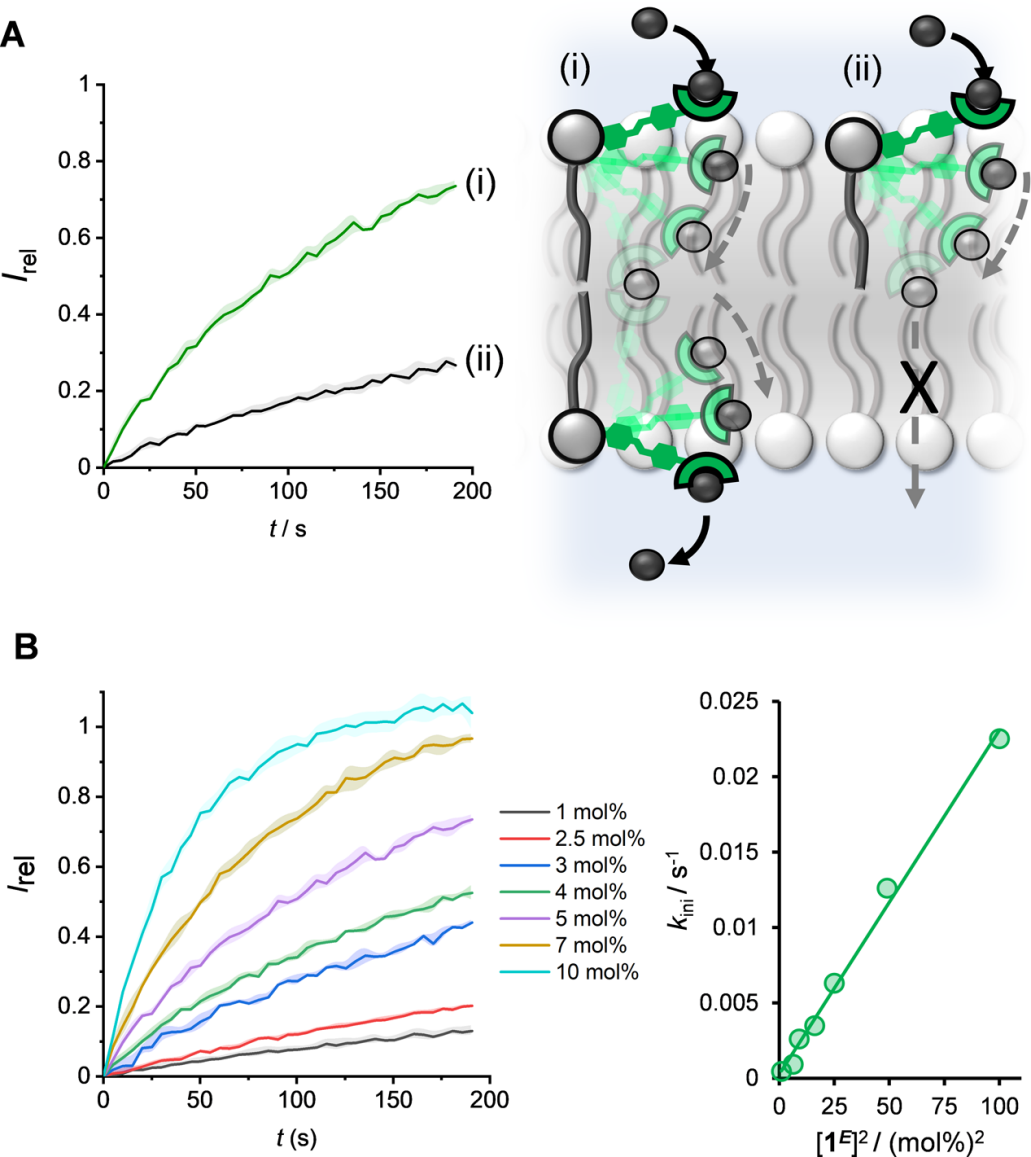
Relay anion transport. (A) Change in ratiometric emission, *I*_rel_ (λ_em_ = 510 nm; λ_ex1_ = 405 nm, λ_ex2_ = 460 nm), upon addition of a NaOH base pulse (5 mM) to POPC LUVs (31 μM) containing 1 mM HPTS, 100 mM internal and external NaCl, buffered with 10 mM HEPES at pH 7.0. (i) Data for **1**^***E***^ pre-incorporated during LUV preparation (5 mol % with respect to lipid) and (ii) following external addition of 5 mol % **1**^***E***^ in DMSO (∼90% membrane incorporation efficiency). (B) Concentration dependence of relay activity with pre-incorporated **1**^***E***^ (left) and linear relationship for the initial rate, *k*_ini_ with [**1**^***E***^]^2^ (right).

Relay transporter **1**^***E***^ proved to be an effective anion transporter (green data, Figure 3A). The analogous control compound **2**, which in contrast to **1**^***E***^ is incapable of binding anions (Figures S61–S63), was inactive, demonstrating the requirement for the thiourea binding group of the relay transporter. The transporters must also be of sufficient length to reach the center of the bilayer in order to pass ions between them: this is possible when **1**^***E***^ is pre-incorporated in the membrane during vesicle preparation but not when the transporters are incorporated exclusively in one leaflet. Addition of **1**^***E***^ to the outer leaflet of the bilayer of pre-formed LUVs could be achieved by injection of a DMSO solution of **1**^***E***^ into the vesicle suspension, with efficient membrane uptake (∼90%) confirmed by UV–vis absorption spectroscopy (Figure S55). However, this led to minimal anion transport activity (Figure 3A, black data), revealing that **1**^***E***^ cannot readily migrate to the inner leaflet, and confirming the necessity of the transporters being present in both leaflets of the membrane in order to facilitate the anion transport relay.

The dependence of the observed initial anion transport rate, *k*_ini_, on the concentration of pre-incorporated **1**^***E***^ was non-linear, indicative of multiple molecules of **1**^***E***^ in the rate-limiting step of the transport process. A linear relationship was obtained for *k*_ini_ versus [**1**^***E***^]^2^, suggesting that two molecules of **1**^***E***^ are required to mediate transport, consistent with the proposed relay mechanism (Figure 3B). Hill analysis^41^ of the dose response curve similarly confirmed that two relay molecules are involved in the rate-limiting step, with the Hill coefficient *n* ∼ 2 (Figure S65).

**Figure 4:**
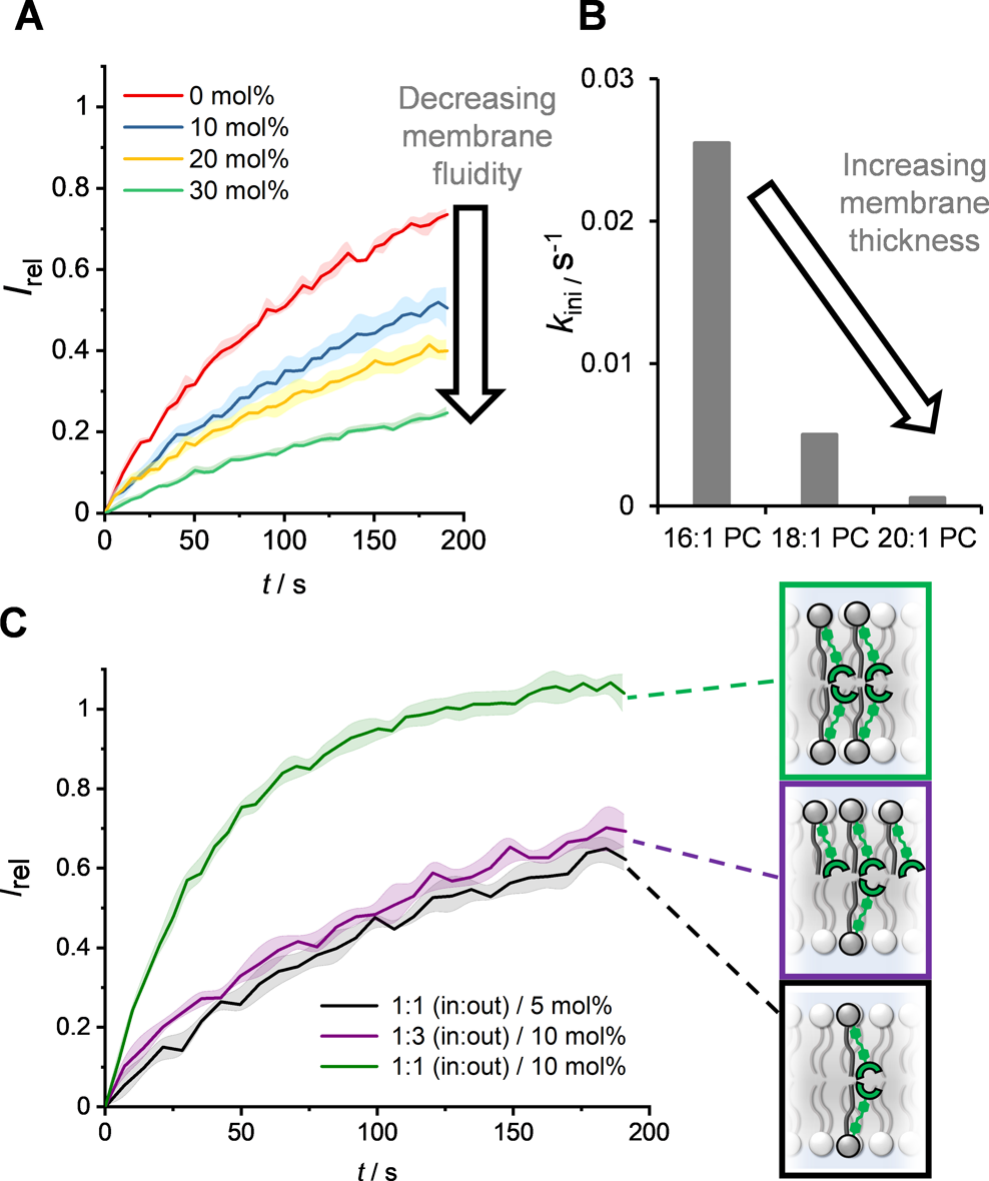
(A) Transport dependence of **1**^***E***^ on membrane cholesterol loading (5 mol % with respect to POPC lipid). (B) Dependence of transport initial rate, *k*_ini_, on lipid membrane thickness. (C) Asymmetric loading of **1**^***E***^ to the inner and outer leaflets of POPC LUVs. Symmetric loading was achieved by pre-incorporation of 5 or 10 mol % **1**^***E***^ during LUV preparation (black and green data, respectively). A 3:1 excess of **1**^***E***^ in the outer leaflet was achieved by addition of a further 5 mol % **1**^***E***^ in DMSO to pre-formed vesicles loaded with 5 mol % **1**^***E***^ (purple data, ∼90% incorporation efficiency). Assay conditions as in Figure 3.

The rate of transport via the **1**^***E***^ relay was unchanged upon exchanging zwitterionic phosphatidylcholine (POPC) lipids with the analogous anionic phosphatidylglycerol lipids, indicating that anion binding or release at the membrane interface is not rate limiting (Figure S68). Anion transport activity decreased with an increase in cholesterol loading, which acts to lower the fluidity of the membrane (Figure 4A). Activity was inhibited in the lipid gel phase of dipalmitoylphosphatidylcholine lipids at 25 °C but restored at 45 °C, above the gel–liquid phase transition temperature (*T*_m_ = 41 °C) (Figure S69). These results demonstrate that the arms of the relay components must be able to freely move between the interface and the center of the membrane, and the rate of this motion is suppressed in less fluid membranes. These experiments also rule out ion channel formation, for which the activity would be expected to be independent of membrane fluidity.

Inactivity of the system when the chloride anions in the buffer were exchanged for gluconate—a large hydrophilic anion too hydrophilic to be transported—confirmed that the observed transport in the presence of chloride is cation independent, occurring via Cl^–^/OH^–^ antiport (or the functionally equivalent Cl^–^/H^+^ symport) and not via a cation-dependent H^+^/Na^+^ antiport process (Figure S70).

To determine the relative rates of Cl^–^ versus H^+^/OH^–^ transport, the ion transport assay with **1**^***E***^ was repeated in the presence of carbonyl cyanide-*p*-trifluoromethoxyphenylhydrazone (FCCP). This protonophore decouples proton transport from the relay-mediated OH^–^/Cl^–^ antiport (or H^+^/Cl^–^ symport process), by mediating fast electrogenic H^+^ transport. Under these conditions, the assay reports on the rate-limiting relay-mediated chloride transport. We observed an increase in transport rate with **1**^***E***^ in the presence of FCCP, indicating that H^+^/OH^–^ transport is rate limiting in the relay mechanism (Figure S71). Repeating these experiments with various monovalent anions in the internal and external buffer revealed an overall selectivity profile for the relay-mediated transport of I^–^ > Br^–^ > Cl^–^ > OH^–^ (Figures S72–S74).

The anion transport activity of **1**^***E***^ in a homologous series of lipids with varying acyl chain lengths (16:1, 18:1, and 20:1 *cis*-phosphatidylcholine) decreased with increasing membrane thickness (Figure 4B). This is consistent with a relay mechanism: at a sufficient membrane thickness a gap between the binding sites of transporters on opposite sides of the membrane is established, such that the energetic barrier for anion relay becomes prohibitive. This behavior contrasts with that observed for ion channels, which display Gaussian-shaped dependence on lipid thickness, whereby maximum activity is observed when the thickness matches the length of the channel,^42^ and for mobile anion carriers, where diffusion across the tail region of the bilayer has been shown to be not rate-limiting.^43^

We explored the effect of an excess of **1**^***E***^ in the outer leaflet of the membrane, relative to that in the inner leaflet, by adding 5 mol % **1**^***E***^ in DMSO to LUVs containing 5 mol % pre-incorporated **1**^***E***^ (affording an asymmetric membrane loading with a ratio of 1:3 inner leaflet/outer leaflet). These LUVs were irradiated with 405 nm light to generate the **1**^***E***^-rich PSS, before the transport assay was initiated with the base pulse (purple data, Figure 4C). Efficient membrane uptake achieved by external addition was confirmed by UV–vis experiments (∼90%, Figure S57). Transport experiments in these vesicles with asymmetric transporter loading did not result in an appreciable change in activity compared to LUVs with a symmetric loading of 5 mol % **1**^***E***^ (black data, Figure 4C). The activity was also significantly lower than that of LUVs with the same total loading of 10 mol % **1**^***E***^ but now with the transporters symmetrically distributed across both leaflets (green data, Figure 4C). This indicates that the rate is dependent on the concentration of the transporter in the inner leaflet, which further supports the conclusion from the experiments with negatively charged lipids which revealed that uptake of the anion from the aqueous phase into the membrane is not rate limiting. Rather, the rate-limiting step must involve the exchange of the anion between the thiourea-binding sites on the relay transporters on opposite sides of the membrane, via a transmembrane 2:1 receptor-anion intermediate. Together with the observed [**1**^***E***^]^2^ dependence of the transport rate, this points to this anion exchange process between thiourea-binding sites being rate limiting. This is plausible given that the low dielectric^44,45^ in the hydrophobic center of the bilayer will result in strong transporter–anion complexation via hydrogen bonding,^46^ compared to that formed at the more polar interface region.

### Photo-switching of Relay Activity

**Figure 5:**
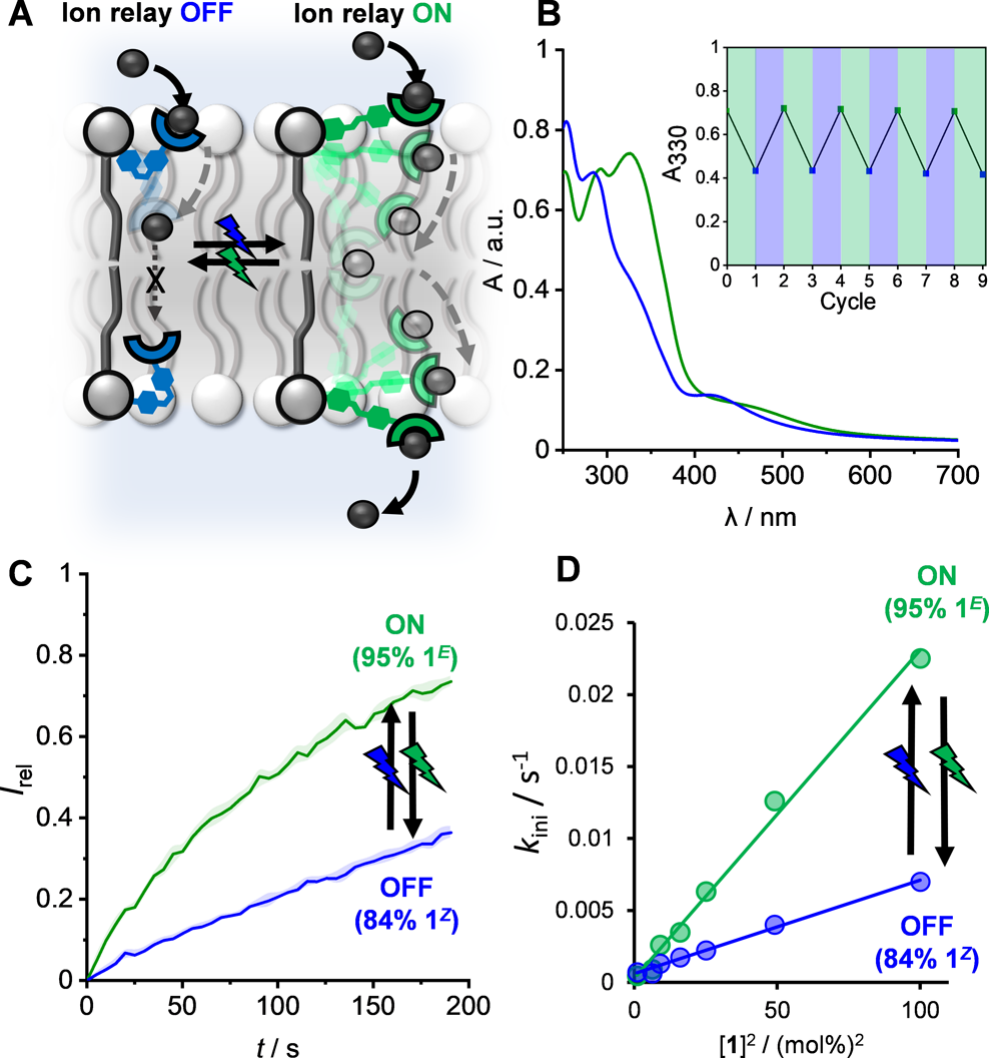
Reversible photo-switching of ion transport relay activity. (A) Schematic representation of the photo-switchable relay. (B) UV–vis spectra of 95% **1**^***E***^ (green) and 84% **1**^***Z***^ PSS (blue) in POPC LUVs; inset: absorption at 330 nm after successive cycles of irradiation with 530/405 nm light. (C) Ion transport by **1**^***E***^**-** and **1**^***Z***^-rich PSSs generated by in situ photo-isomerization of **1** in LUVs at 405 and 530 nm, respectively. (D) Linear dependence of *k*_ini_ on [**1**]^2^ for the **1**^***E***^**-** and **1**^***Z***^-rich PSS generated by in situ photo-isomerization.

Photo-isomerization from **1**^***E***^ to **1**^***Z***^ decreases the effective length of the transporter’s telescopic arm by approximately 0.8 nm. Previous studies have reported that a gap of 1.1 nm is the upper limit for cation hopping between crown ether derivatives in a membrane spanning channel, so we anticipated that the gap between thiourea-binding sites for two **1**^***Z***^ relays (∼1.6 nm) should be sufficient to inhibit transport.^47^ We therefore explored the effect of the photo-driven contraction and expansion process on the ion transport relay by repeating the transport assay with LUVs containing pre-incorporated **1**^***E***^ and following in situ photo-isomerization (Figure 5A).

Irradiation of LUVs containing pre-incorporated **1**^***E***^ with 530 nm light generated **1**^***Z***^ within the membrane, with a PSS distribution identical to that obtained in DMSO solution (84% **1**^***Z***^), as determined by UV–vis absorption spectroscopic analysis (Figure S59). As in the solution phase, switching of membrane-embedded **1**^***Z***^ in the reverse direction was achieved with 405 nm light, and this switching process was fully reversible and could be repeated multiple times without detectable fatigue (Figure 5B). No thermally promoted **1**^***Z***^ → **1**^***E***^ isomerization was observed in the membrane at 298 K. Heating LUVs containing the **1**^***Z***^-rich PSS distribution to 333 K allowed the determination of a thermal half-life of 24 h at this elevated temperature (Figure S60). Ion transport facilitated by the **1**^***Z***^-rich PSS distribution was significantly reduced relative to that of **1**^***E***^ (Figure 5C). This is attributed to the contracted state of the relay molecule in the *Z* isomer, which inhibits the exchange of the anion between thiourea-binding groups of transporters in opposite leaflets of the membrane. Switching of activity between the extended (ON) and contracted (OFF) states is fully reversible: the observed transport rates for each state were identical regardless of the order of photo-isomerization. Photo-irradiation of vesicles containing a simple model thiourea mobile carrier showed no alteration in transport activity (Figure S76). Furthermore, control experiments with in situ photo-switching of **2**^***E***^ (lacking the anion-binding site) did not lead to transport, ruling out photo-thermal effects (Figure S77). Together, these control experiments demonstrate that photo-isomerization does not lead to membrane disruption and non-specific ion leakage.

Figure 5D shows the concentration dependence of *k*_ini_ for both PSS distributions. As observed for **1**^***E***^, a linear relationship was obtained for *k*_ini_ versus [**1**^***Z***^]^2^, suggesting that residual transport activity in this state also requires two molecules to mediate transport. This is attributed primarily to the remaining 16% **1**^***E***^ present in the PSS distribution (via a combination of both **1**^***E***^–**1**^***E***^ and **1**^***E***^–**1**^***Z***^ relay). When added externally, both **1**^***E***^ and **1**^***Z***^ led to identical minimal background transport activity, resulting from their presence in only the outer leaflet of the bilayer and thus not forming the necessary transmembrane relay assembly (Figure S78).

**Figure 6:**
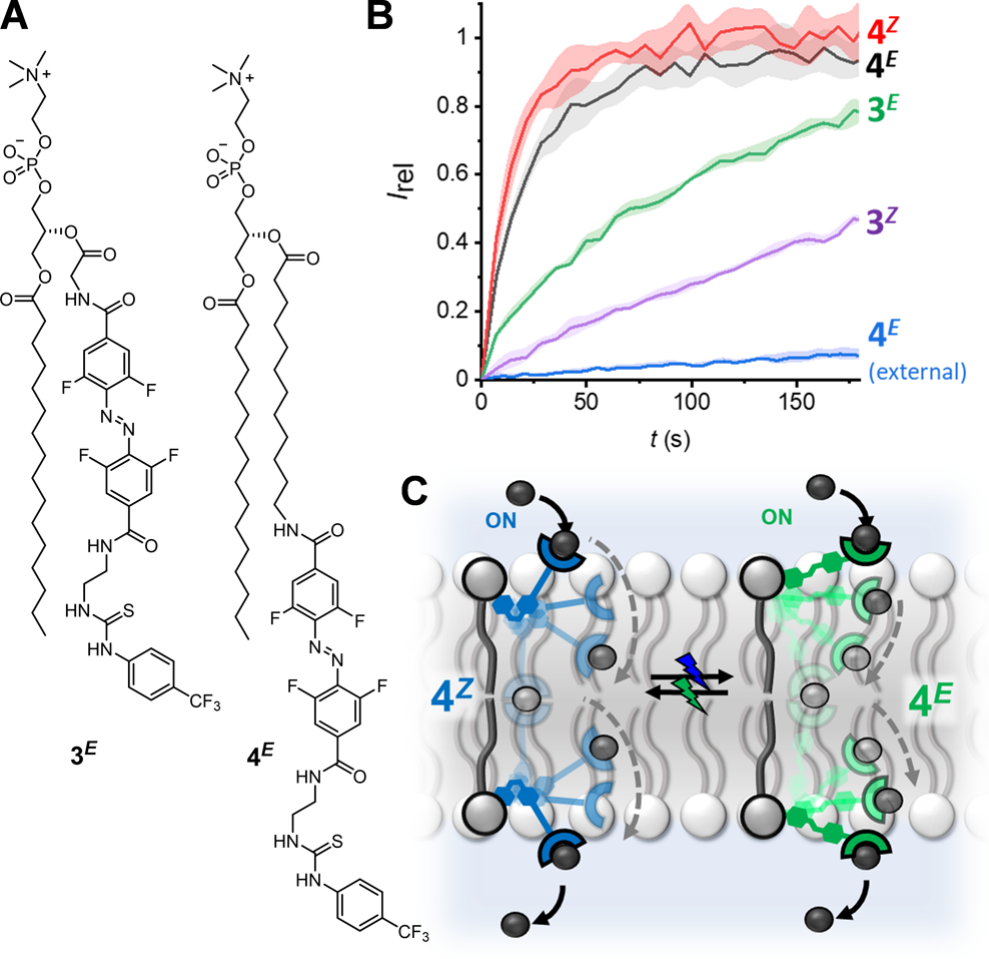
Effect of relay transporter length on photo-switchable transport. (A) Chemical structures of longer transporters **3** and **4**. (B) Ion transport by 5 mol % pre-incorporated **3** and **4** with in situ photo-switching and by 5 mol % **4**^***E***^ following external addition (blue). (C) Schematic representation of relay transport for both **4**^***Z***^ and **4**^***E***^. Conditions as in Figure 3.

Key to the control of this machine-like ion relay is the photo-driven extension and contraction of the telescopic arm, which switches between the ON and OFF states, respectively. To explore the effect of the length of the telescopic arm on the switchable relay transport, we prepared the longer derivatives **3** and **4** (Figure 6A), anticipating that for a system with a sufficiently long relay arm—such that the *Z* isomer is also able to act as a relay—there would be no observable switching of transport activity. Synthetic procedures and characterization for **3** and **4** are available in the Supporting Information (Section S2), along with data for all ion transport experiments (Figures S79–S88).

We first investigated compound **3**, in which the telescopic arm is longer than that of compound **1** by three atoms. Analogous anion-selective relay transport behavior to **1** was observed (Figures S79–S84) with similar length-dependent behavior in membranes of varying hydrophobic thicknesses (Figure S80), comparable photo-switching properties (PSS_530_ = 90% **3**^***Z***^; PSS_405_ = 96% **3**^***E***^), and in situ photo-switching of transport activity (Figure 6B, green and purple data). Notably, the transport rates achieved for in situ switching between **3**^***E***^ and **3**^***Z***^ were the same as those obtained by pre-incorporation of ex situ prepared **3**^***Z***^ (in which the photo-isomerization of **3**^***E***^ to **3**^***Z***^ was carried out in solution prior to pre-incorporation within the membrane during vesicle preparation, Figure S82). This further supports our observation that efficient in situ photo-isomerization of the relay transporters is readily achieved in the bilayer and again confirms that non-specific photo-thermal effects—that may in principle contribute to changes in membrane ion permeability—are not responsible for the observed activity.

For the longer relay **3**, we observed a decrease in the ratio of transport rates for each isomer (*k*_ini,3E_/*k*_ini,3Z_ = 2.2 ± 0.1), compared to *k*_ini,1E_/*k*_ini,1Z_ = 3.0 ± 0.1 for the shorter relay **1** at 5 mol % loading, consistent with the increase in length of the transporter, enhancing the relative activity of the *Z*-isomer. For relay **4**, in which the telescopic arm is an additional 10 atoms longer, we observed no significant change in activity upon in situ photo-switching between the two isomers (*k*_ini,4E_/*k*_ini,4Z_ = 0.8 ± 0.2 at 5 mol % loading), implying that both **4**^***E***^ and **4**^***Z***^ are effective transporters (Figure 6B, black and red data) and that **4**^***Z***^ is sufficiently long to mediate relay transport across the bilayer (Figure 6C). As with **1**^***E***^, transporter **4**^***E***^ exhibited minimal transport activity upon external addition into the outer leaflet of the bilayer (Figure 6B, blue data), signifying that the transporter is incapable of facilitating transport when solely located in the outer leaflet and therefore must be acting as a relay when incorporated in both leaflets of the membrane. Both isomers of **4** are more active than either **1**^***E***^ or **3**^***E***^, suggesting that increased length and flexibility may be advantageous for relay transport.

Together, these results demonstrate the critical role of the telescopic arm length for controlling the relay transport process: the gap between the thiourea anion-binding sites of transporters on opposite sides of the membrane must be sufficiently small to mediate transport for the *E* isomer but also sufficiently large for the *Z* isomer in order to suppress transport in the OFF state. Incorporating a mechanism for controlling the length of the relay components—as demonstrated here by using a photo-switchable telescopic arm—thus provides a novel mechanism by which to regulate transmembrane ion transport. This mechanism requires the unprecedented control of multiple molecular machine-like components positioned on opposite sides of a membrane, which work together in a cooperative manner to facilitate transport.

## Conclusions

We have demonstrated that a photo-switchable relay mechanism controls anion transport across lipid bilayer membranes, by modulating the length of the relay components that are positioned on opposite sides of the membrane. The molecular machine-like telescopic arms are reversibly lengthened and contracted by photo-isomerization using visible light. This provides a mechanism for regulating the ion transport process, because efficient transport occurs only in the extended state, in which the length of the telescopic arms is sufficient to pass the anion between them and across the bilayer. Crucially, two relay components are required to work cooperatively in order to mediate the transport process. This work suggests that confining ensembles of artificial molecular machines within membranes provides a powerful method to control their relative positions and orientations, and thus access molecular machine-like systems in which multiple components work in concert. This approach is likely to be a powerful method to translate the nano-mechanical motion of molecular machines into useful functions in a biological context.

## Supporting Information Available

Experimental procedures, characterization of new compounds, UV–vis studies, and anion transport studies (PDF)
