# Supplementary material for: A Photo-responsive Transmembrane Anion Transporter Relay
Source: J Am Chem Soc. 2022 Jun 2;144(23):10455–61. doi: 10.1021/jacs.2c02612 (PMC9204766; doi:10.1021/jacs.2c02612)
Supplement: Supplementary file 1 — ja2c02612_si_001.pdf [file ja2c02612_si_001.pdf]

Supporting Information for

A photo-responsive transmembrane anion transport relay

Toby G. Johnson[a], Amir Sadeghi-Kelishadi[a] and Matthew J. Langton[a]*

[a]Chemistry Research Laboratory, University of Oxford, Mansfield Road, Oxford, OX1 3TA, UK

Contents

1 Materials and Methods 2
2 Synthesis and Characterization 3
3 Photo-isomerisation Experiments 40
4 UV-Visible Absorption Analysis 42
5 ^{1}H NMR Titration Experiments 52
6 Anion Transport Experiments 54
7 References 67

1 Materials and Methods

All reagents and solvents were purchased from commercial sources and used without further purification. Lipids were purchased from Avanti polar lipids and used without further purification. Where necessary, solvents were dried by passing through an MBraun MPSP-800 column and degassed with nitrogen. Triethylamine was distilled from and stored over potassium hydroxide. Normal phase silica gel flash column chromatography was performed either manually using Merck® silica gel 60 under a positive pressure of nitrogen or on a Buchi Pure C-815 Flash automated column chromatography system using FlashPure EcoFlex silica cartridges. Where mixtures of solvents were used, ratios are reported by volume. NMR spectra were recorded on a Bruker AVIII 400, Bruker AVII 500 (with cryoprobe) and Bruker AVIII 500 spectrometers. Chemical shifts are reported as δ values in ppm. Mass spectra were carried out on a Waters Micromass LCT and Bruker microTOF spectrometers. Fluorescence spectroscopic data were recorded using a Horiba Duetta fluorescence spectrophotometer, equipped with a Peltier temperature controller and stirrer. UV-Vis spectra were recorded on a V-770 UV-Visible/NIR Spectrophotometer equipped with a Peltier temperature controller and stirrer, using quartz cuvettes of 1 cm path length. Experiments were conducted at 25 °C unless otherwise stated. Vesicles were prepared as described below using Avestin "LiposoFast" extruder apparatus, equipped with polycarbonate membranes with 200 nm pores. GPC purification of vesicles was carried out using GE Healthcare PD-10 desalting columns prepacked with Sephadex G 25 medium.

Abbreviations

16:0 Lyso PC: 1-Palmitoyl-2-hydroxy-sn-glycero-3-phosphocholine; **16:1 PC**: 1,2-Dipalmitoleoyl-sn-glycero-3-phosphocholine; **18:1 PC**: 1,2-Dioleoyl-sn-glycero-3-phosphocholine (DOPC); **20:1 PC**: 1,2-Dieicosenoyl-sn-glycero-3-phosphocholine; **Boc**: *tert*-Butyloxycarbonyl; **DBU**: 1,8-Diazabicyclo[5.4.0]undec-7-ene; **DIC**: *N*,*N*-Diisopropylcarbodiimide; **DIPEA**: *N,N*-Diisopropylethylamine; **DMAP**: 4-Dimethylaminopyridine; **DPPC**: 1,2-Dipalmitoyl-sn-glycero-3-phosphocholine; **EYPG**: Egg-yolk phosphatidylglycerol; **FCCP**: Carbonyl cyanide-p-trifluoromethoxyphenylhydrazone; **Fmoc**: 9-Fluorenylmethoxycarbonyl; **HATU**: 1-[Bis(dimethylamino)methylene]-1H-1,2,3-triazolo[4,5-b]pyridinium 3-oxide hexafluorophosphate; **HEPES**: *N*-(2-Hydroxyethyl)piperazine-*N*'-(2-ethanesulfonic acid); **HPTS**: 8-Hydroxy-1,3,6-pyrenetrisulfonate; **HRMS**: High resolution mass spectrometry; **LUVs**: Large unilamellar vesicles; **Mtt**: 4-Methyltrityl; **NCS**: *N*-Chlorosuccinimide; **NHS**: *N*-Hydroxysuccinimide; **POPC**: 1-Palmitoyl-2-oleoyl-sn-glycero-3-phosphocholine; **POPG**: 1-Palmitoyl-2-oleoyl-sn-glycero-3-phospho-(1'-rac-glycerol) sodium salt; **rt**: Room temperature; **µw**: Microwave irradiation.

2 Synthesis and Characterization

2.1 General comments.

Compounds **6**[1]; **7**[2], **14**[2], **15**[2], **18**[2], **11**[3], **16**[4], **17**[5], **22**[6], **23**[7], **28**[8] and **29**[9] were prepared according to literature procedures.

Peaks for the azobenzene *E* isomer major product are reported in cases where a minor proportion of Z isomer was also formed.

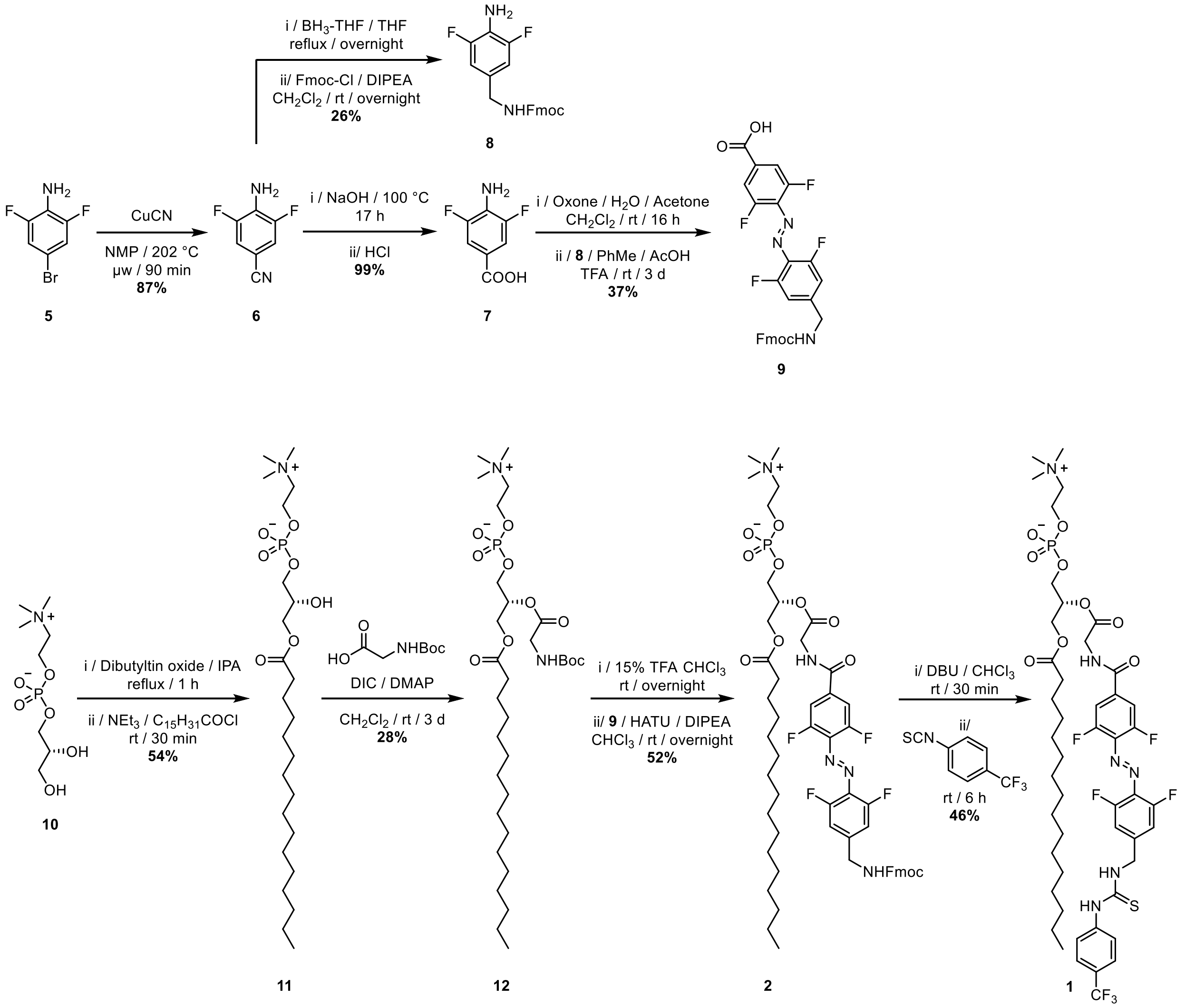

Scheme S1. *Synthesis of relay transporter **1***

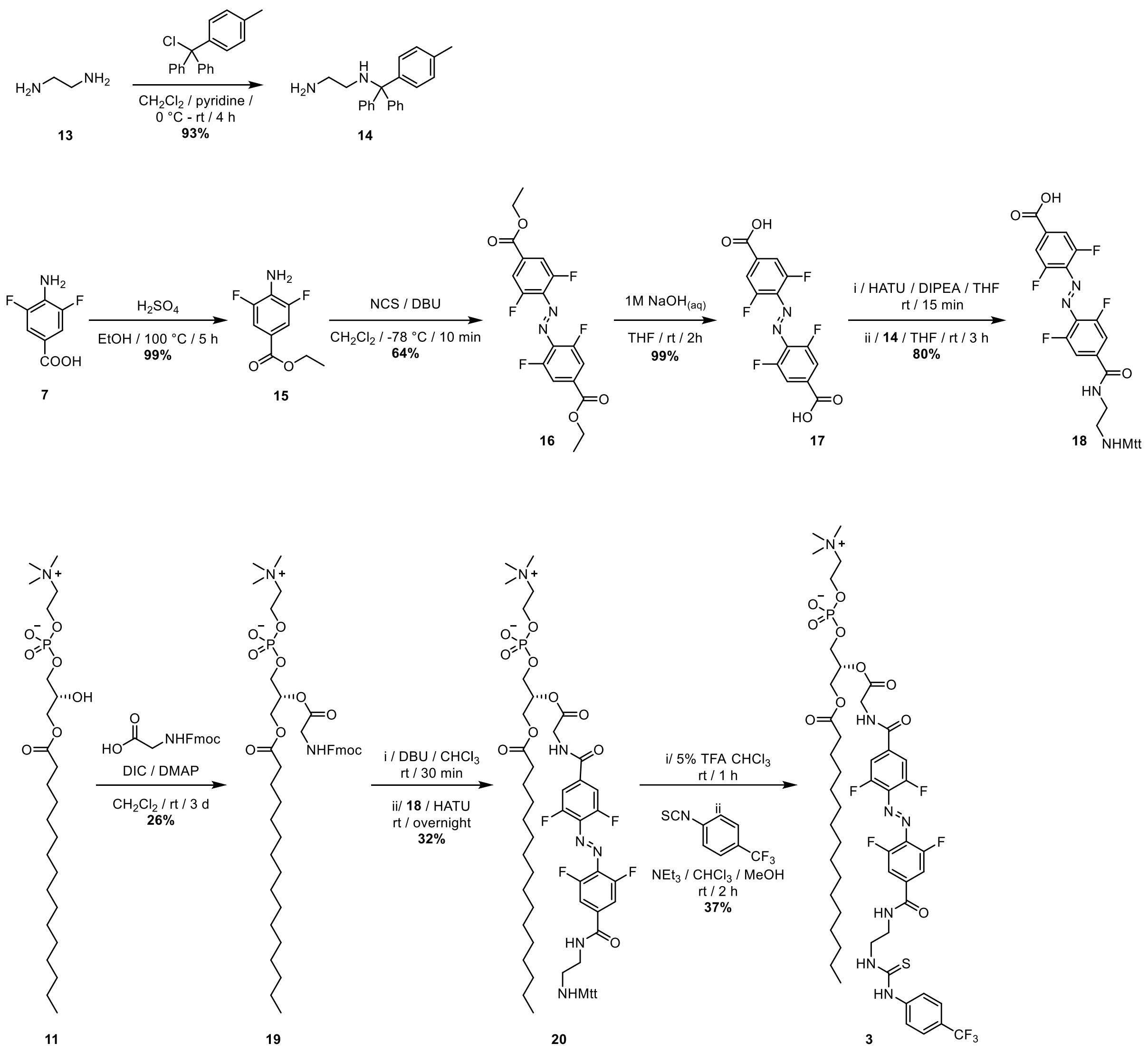

Scheme S2. *Synthesis of medium-length anion relay transporter **3**.*

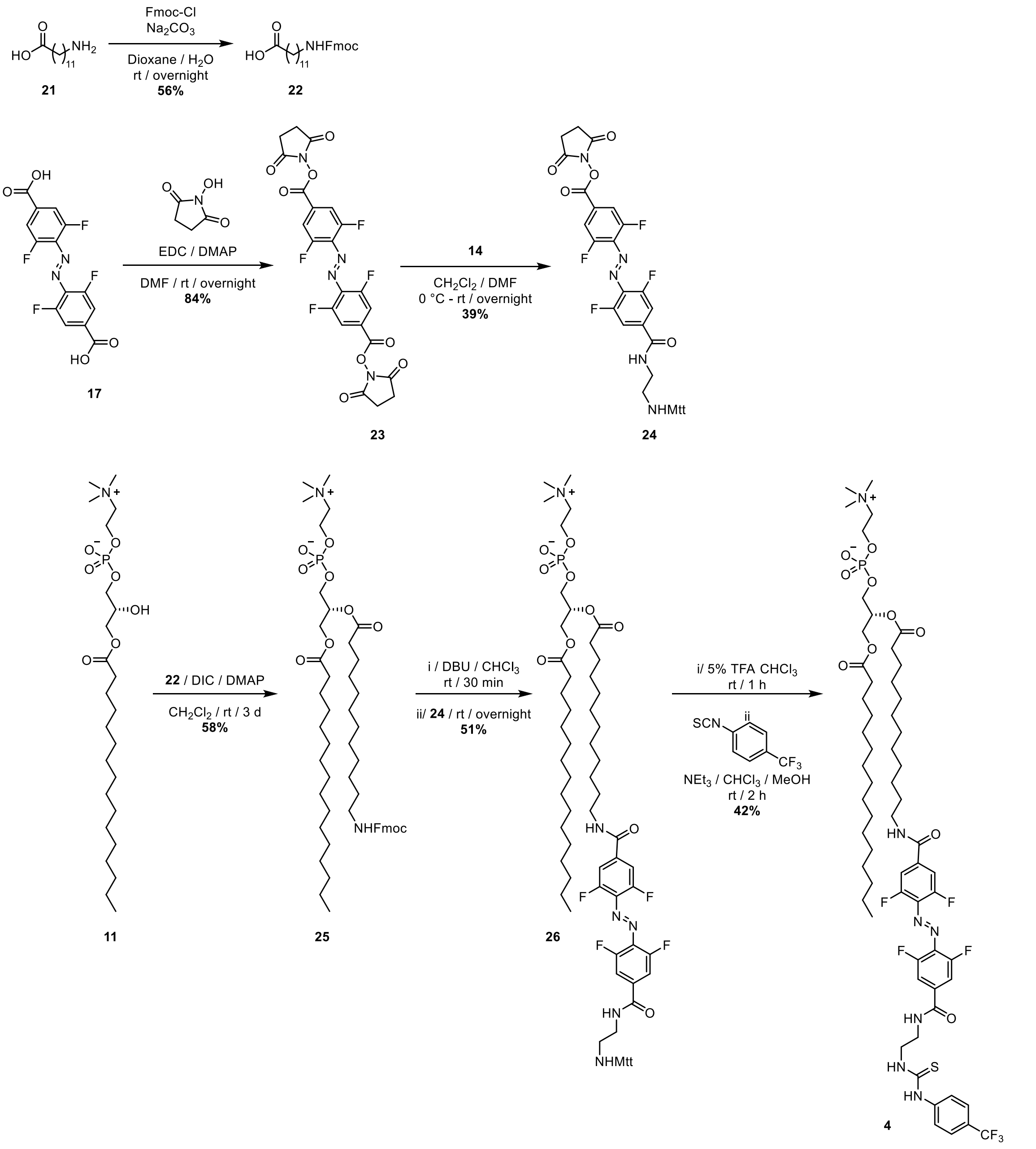

Scheme S3**. Synthesis of longer anion relay transporter **4.

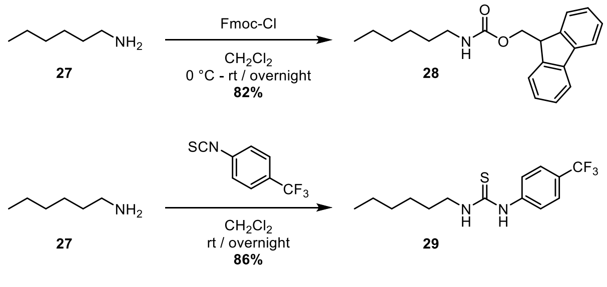

***Scheme S4.** Synthesis of mobile carrier control compounds **28** and **29**.*

2.2 9-Fluorenylmethyl (4-amino-3,5-difluorobenzyl)carbamate 8.

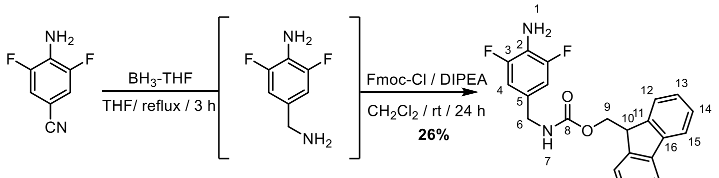

To a solution of 4-amino-3,5-difluorobenzonitrile **6** (1.45 g, 9.41 mmol, 1 equiv.) in anhydrous degassed THF (20 mL) at 0°C was added dropwise 1M BH_3-THF solution (38.4 mL, 38.4 mmol, 4 equiv.). The reaction was stirred at reflux for 3 hours. MeOH (5mL) was added dropwise at this temperature until effervescence stopped, and the resulting mixture was refluxed for 30 min. The solvent was removed *in vacuo*, redissolved in EtOAc (100 mL) and washed with water (3 × 50 mL). The off-white solid was redissolved in anhydrous CH_2Cl_2 (30 mL) and DIPEA (1.6 mL, 9.41 mmol, 1 equiv.) was added. Fmoc-Cl (2.43 g, 9.41 mmol, 1 equiv.) in anhydrous CH_2Cl_2 (5 mL) was added dropwise and the reaction was stirred overnight at rt under N_2. The solution was concentrated *in vacuo* and the residue was purified by column chromatography (Buchi Pure C-815 Flash, 24 g SiO_2, hexane:acetone 0 – 20%) to give the title compound as a white solid (0.93 g, 2.4 mmol, 26%).

^{1}H NMR (400 MHz, $CDCl_3$) δ 7.76 (d, *J* = 7.6 Hz, 2H, H_{15}), 7.59 (d, *J* = 7.5 Hz, 2H, H_{12}), 7.40 (t, *J* = 7.5 Hz, 2H, H_{13}), 7.31 (t, *J* = 7.5 Hz, 2H, H_{14}), 6.75 (d, *J* = 7.4 Hz, 2H, H_4), 5.02 (s, 1H, H_7), 4.47 (d, *J* = 6.8 Hz, 2H, H_9), 4.30 – 4.01 (m, 3H, H_6 & H_{10}), 3.70 (s, 2H, H_1).

^{13}C NMR (126 MHz, $CDCl_3$) δ 156.5 (C_8), 152.1 (dd, *J* = 241.2, 8.2 Hz) (C_3), 144.0, 141.5, 127.9 (C_{13}), 127.8 (C_5), 127.2 (C_{14}), 125.1 (C_{12}), 123.2 (t, *J* = 16.2 Hz) (C_2), 120.1 (C_{15}), 110.3 (dd, *J* = 15.7, 6.7 Hz) (C_4), 66.9 (C_9), 47.4, 44.3.

^{19}F NMR (377 MHz, $CDCl_3$) δ -131.69 (d, *J* = 7.4 Hz) (F_3).

HRMS-ESI (m/z) Calculated for $C_{22}H_{18}F_2N_2O_2$ $[M+H]^+$, 381.1409; found 381.1408.

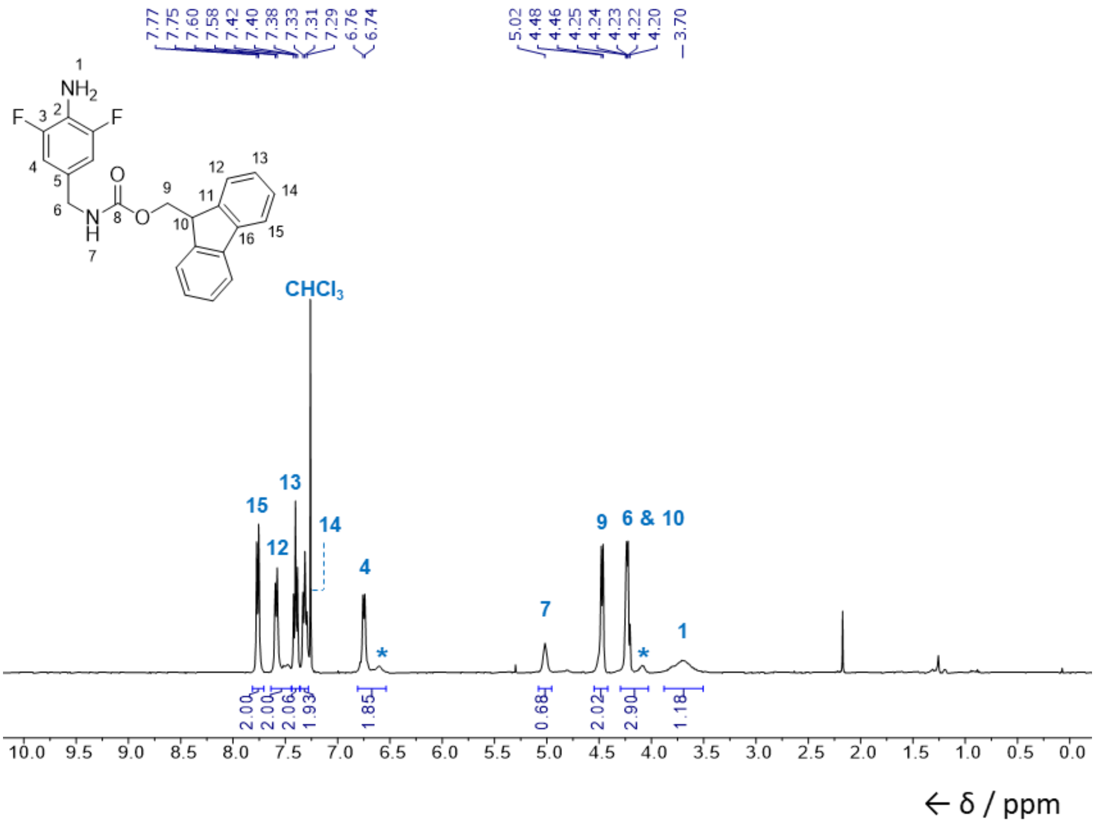

Figure S1. *^{1}H NMR spectrum of compound **8** ($CDCl_3$, 400 MHz, 298 K). Rotameric signals are labelled with a blue asterisk (*)*

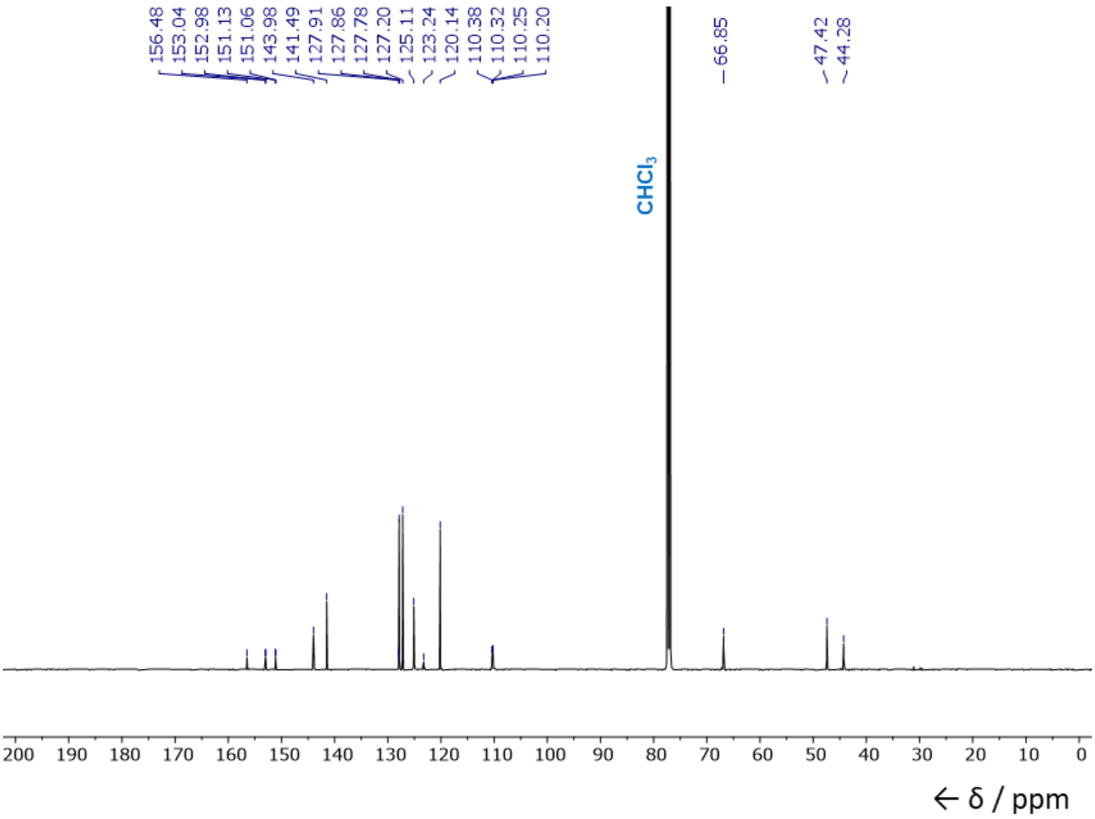

Figure S2. *^{13}C NMR spectrum of compound **8** ($CDCl_3$, 126 MHz, 298 K).*

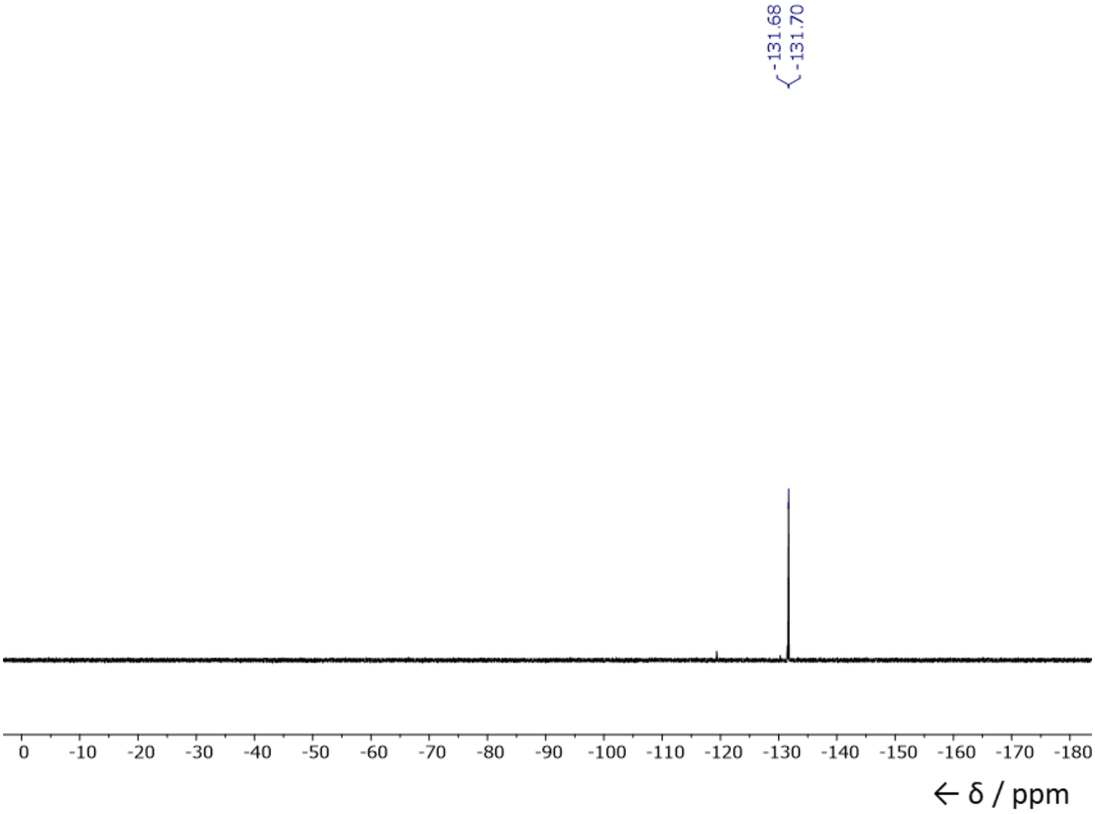

Figure S3. *^{19}F NMR spectrum of compound **8** ($CDCl_3$, 377 MHz, 298 K).*

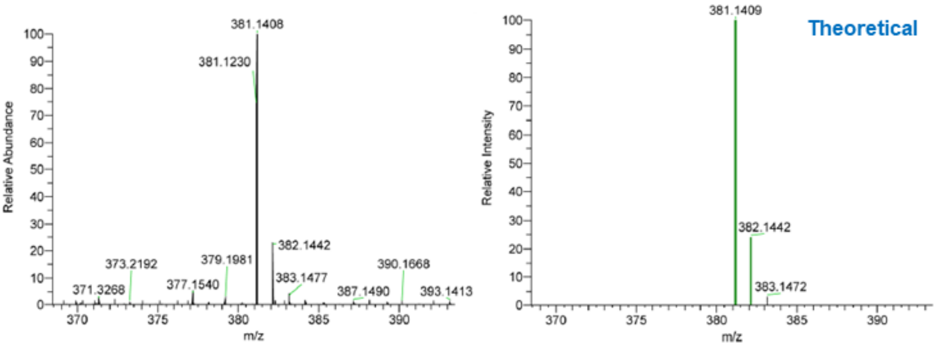

Figure S4. *HRMS spectrum of compound **8**. HRMS-ESI (m/z) calculated for $C_{22}H_{18}F_2N_2O_2$ $[M+H]^+$, 381.1409; found 381.1408.*

2.3 (*E*)-4-((4-(((((9H-fluoren-9-yl)methoxy)carbonyl)amino)methyl)-2,6-difluorophenyl)diazenyl)-3,5-difluorobenzoic acid 9.

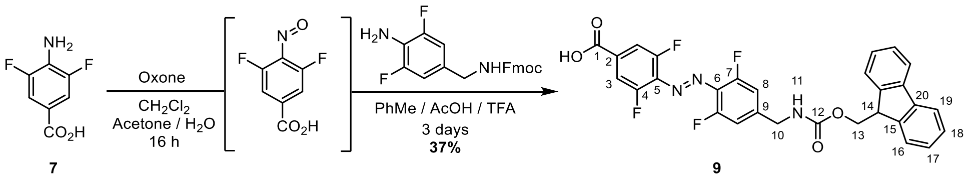

4-Amino-3,5-difluorobenzoic acid **7** (510 mg, 3.0 mmol, 1.7 equiv.) was dissolved in CH_2Cl_2:acetone ((5:1), 12 mL). Oxone (3 g, 9.8 mmol, 5.5 eq) in water (6 mL) was added and the biphasic solution was stirred overnight, after which the organic layer turned green. The reaction mixture was diluted with water (50 mL) and extracted with EtOAc (3 × 50 mL), the organic layers were combined and concentrated. The residue was redissolved in AcOH:toluene:TFA ((6:6:1), 18 mL) and 9-fluorenylmethyl (4-amino-3,5-difluorobenzyl)carbamate **8** (675 mg, 1.8 mmol, 1 equiv.) was added to the stirred reaction mixture which was left stirring at rt under N_2 for 3 days. The mixture was concentrated and the residue was purified by silica gel flash chromatography (1 – 8% MeOH / CH_2Cl_2) to give the title compound as an orange solid (358 mg, 0.65 mmol, 37%).

^{1}H NMR (600 MHz, acetone-d$_6$) δ 7.86 (d, *J* = 7.5 Hz, 2H, H_{19}), 7.80 (d, *J* = 9.4 Hz, 2H, H_3), 7.71 (d, *J* = 7.4 Hz, 2H, H_{16}), 7.41 (t, *J* = 7.4 Hz, 2H, H_{18}), 7.33 (t, *J* = 7.4 Hz, 2H, H_{17}), 7.21 (d, *J* = 10.8 Hz, 2H, H_8), 4.49 – 4.45 (m, 4H, $H_{10\,\&\,13}$), 4.27 (t, *J* = 6.7 Hz, 1H, H_{14}).

^{13}C NMR (151 MHz, acetone-d$_6$) δ 165.0 (C_{12}), 157.5 (d, *J* = 7.8 Hz) (C_1), 156.9 (dd, J = 135.0, 4.0 Hz), 155.2 (dd, J = 132.6, 4.1 Hz), 148.5 (t, *J* = 9.1 Hz) (C_2), 145.2, 142.2, 135.1 (t, *J* = 10.8 Hz), 134.7 (t, *J* = 8.6 Hz), 130.7 (t, *J* = 9.9 Hz) (C_9), 128.6 (C_{18}), 127.9 (C_{17}), 126.0 (C_{16}), 120.8 (C_{19}), 114.8 (dd, *J* = 21.4, 3.9 Hz) (C_3), 112.2 (dd, *J* = 21.4, 1.8 Hz) (C_8), 67.0 (C_{14}), 48.2 (C_{13}), 44.5 (d, *J* = 18.0 Hz) (C_{10}).

^{19}F NMR (377 MHz, acetone-d$_6$) δ -120.76 ($F_{4\text{ or }7}$), -122.02 ($F_{4\text{ or }7}$).

HRMS-ESI (m/z) Calculated for $C_{29}H_{20}N_3O_4F_4$ $[M+H]^+$, 550.1384; found 550.1381.

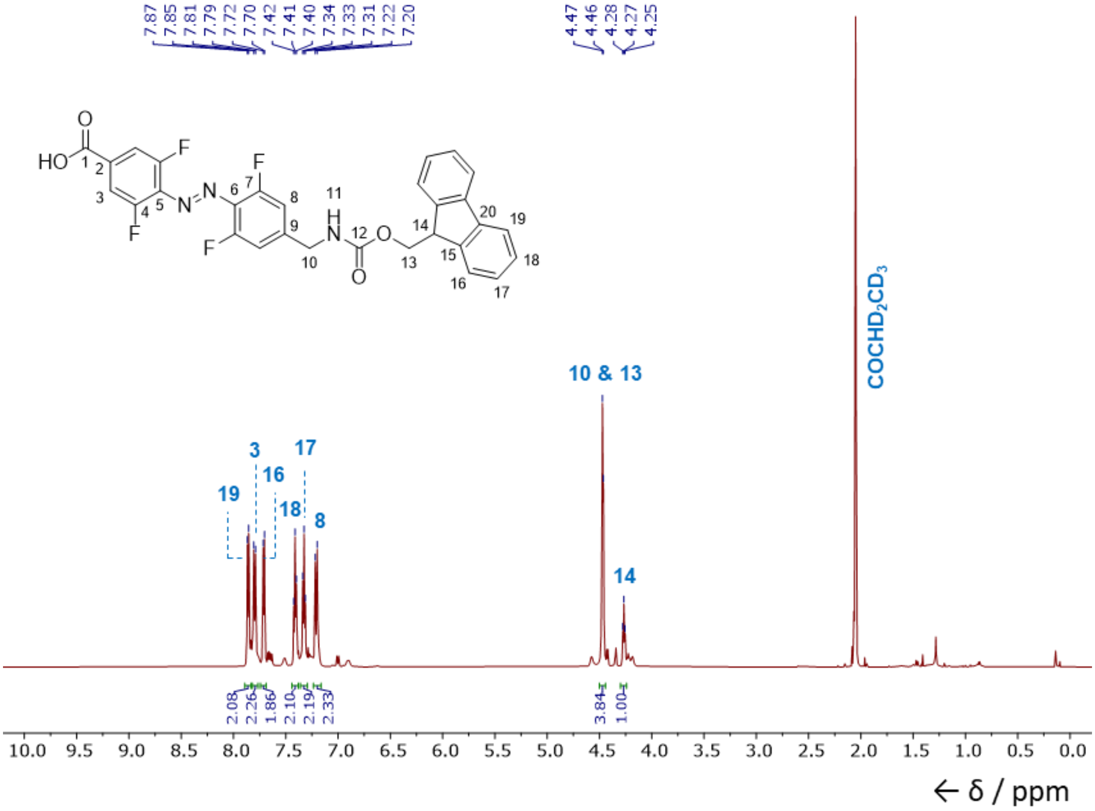

Figure S5. *^{1}H NMR spectrum of compound **9** (acetone-d6, 600 MHz, 298 K).*

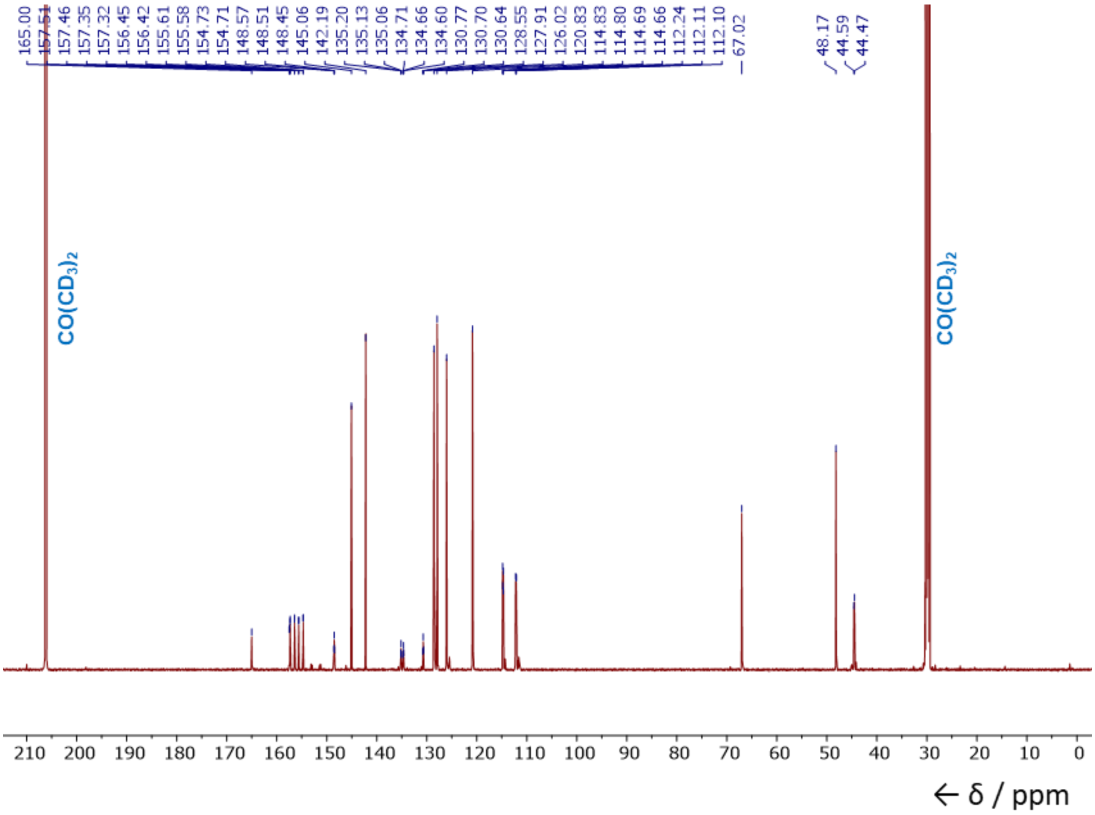

Figure S6. *^{13}C NMR spectrum of compound **9** (acetone-d6, 151 MHz, 298 K).*

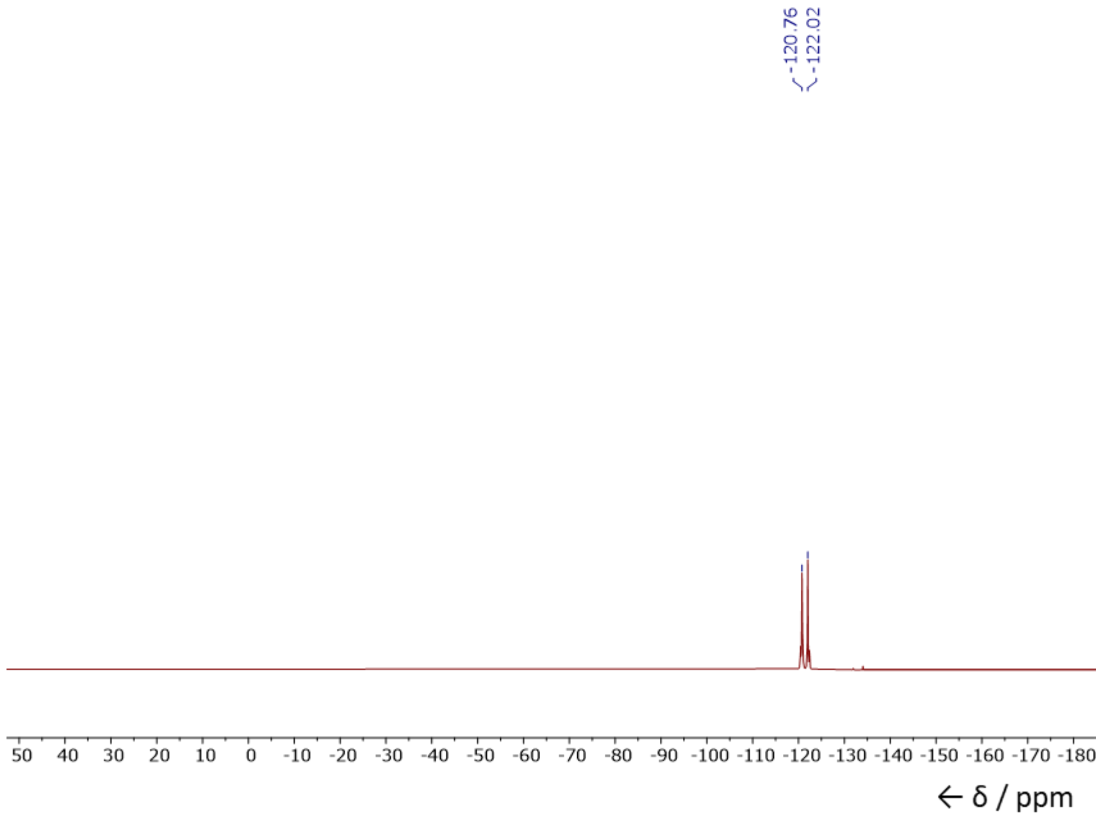

Figure S7. *^{19}F NMR spectrum of compound **9** (acetone-d6, 377 MHz, 298 K).*

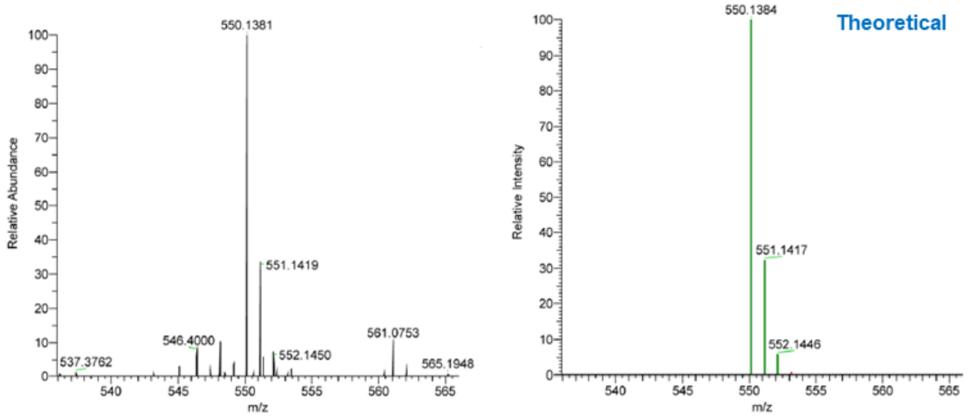

Figure S8. *HRMS spectrum of compound **9**. HRMS-ESI (m/z) Calculated for $C_{29}H_{20}N_3O_4F_4$ [M+H]$^+$, 550.1384; found 550.1381.*

2.4 (*R*)-1-palmitoyl-2-(*N*-boc-glycyl)-*sn*-glycero-3-phosphocholine 12.

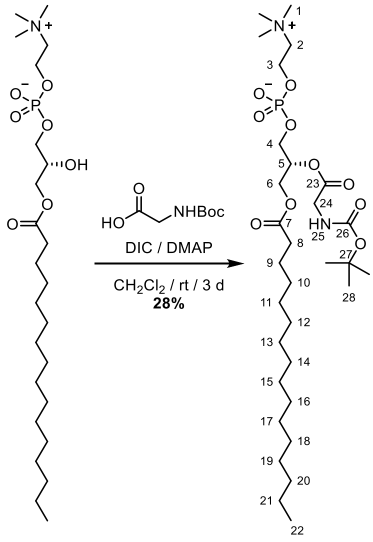

11 **12**

This known compound[10] was prepared *via* a novel procedure: Boc-glycine (1.24 g, 7.1 mmol, 5 Equiv.), DIC (1.8 mL, 11.5 mmol, 8 Equiv.) and DMAP (250 mg, 2.1 mmol, 1.5 Equiv.) were added to a solution of 16:0 lyso PC **11** (700 mg, 1.4 mmol, 1 Equiv.) in anhydrous CH_2Cl_2 (100 mL) under N_2. After stirring the reaction mixture for 3 days, DOWEX 50 WX2-400 ion exchange resin was added to remove DMAP. The solution was allowed to stir for 45 min. The reaction mixture was filtered, washed with $CHCl_3$:MeOH (1:1, 100 mL) and concentrated. The crude material was purified by silica gel flash chromatography (0 - 4% H_2O / 25% MeOH / $CHCl_3$) to give the title compound as a white solid (260 mg, 0.40 mmol, 28%). The characterisation matches that reported in the literature.[10]

^{1}H NMR (400 MHz, $CDCl_3$:CD_3OD (2:1)) δ 5.30 – 5.17 (m, 1H, H_5), 4.37 – 4.31 (m, 1H, H_6), 4.26 – 4.19 (m, 2H, H_3), 4.15 (dd, *J* = 12.1, 6.6 Hz, 1H, H_6), 4.07 – 3.92 (m, 2H, H_4), 3.84 (s, 2H, H_{24}), 3.62 – 3.55 (m, 2H, H_2), 3.19 (s, 9H, H_1), 2.29 (t, *J* = 7.6 Hz, 2H, H_8), 1.60 – 1.52 (m, 2H, H_9), 1.42 (s, 9H, H_{28}), 1.29 – 1.19 (m, 24H, H_{10-21}), 0.84 (t, *J* = 6.8 Hz, 3H, H_{22}).

^{13}C NMR (101 MHz, $CDCl_3$:CD_3OD (2:1)) δ 174.4, 170.7, 157.1, 80.2, 72.1 (d, *J* = 7.2 Hz), 66.9 – 66.7 (m), 64.1 (d, *J* = 5.2 Hz), 62.7, 59.5 (d, *J* = 5.0 Hz), 54.5 (t, *J* = 3.5 Hz), 42.5, 34.3, 32.3, 30.0, 30.0, 30.0, 30.0, 30.0, 29.8, 29.8, 29.7, 29.6, 29.5, 28.5, 25.2, 23.0, 14.3.

LRMS-ESI (m/z) Calculated for $C_{31}H_{62}N_2O_{10}P$ $[M+H]^+$, 653.41; found 653.34.

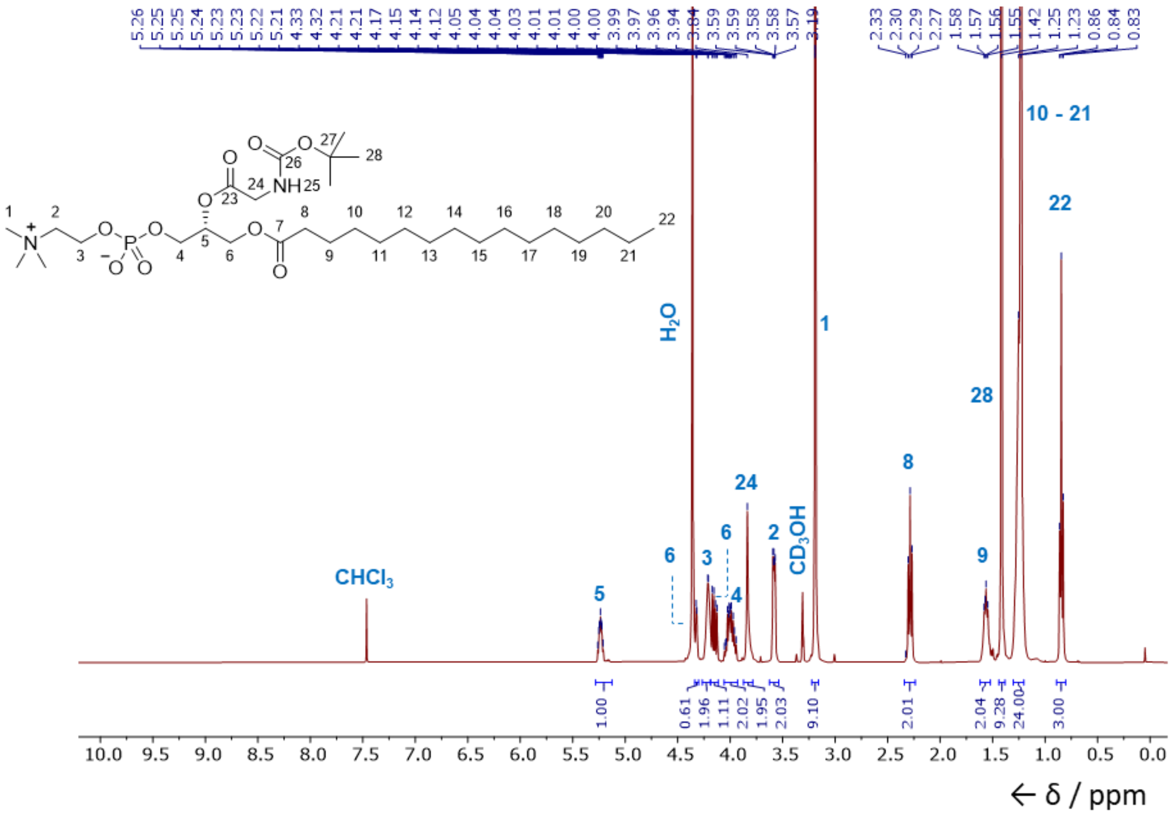

Figure S9. *^{1}H NMR spectrum of compound **12** (CDCl3:CD3OD (2:1), 400 MHz, 298 K).*

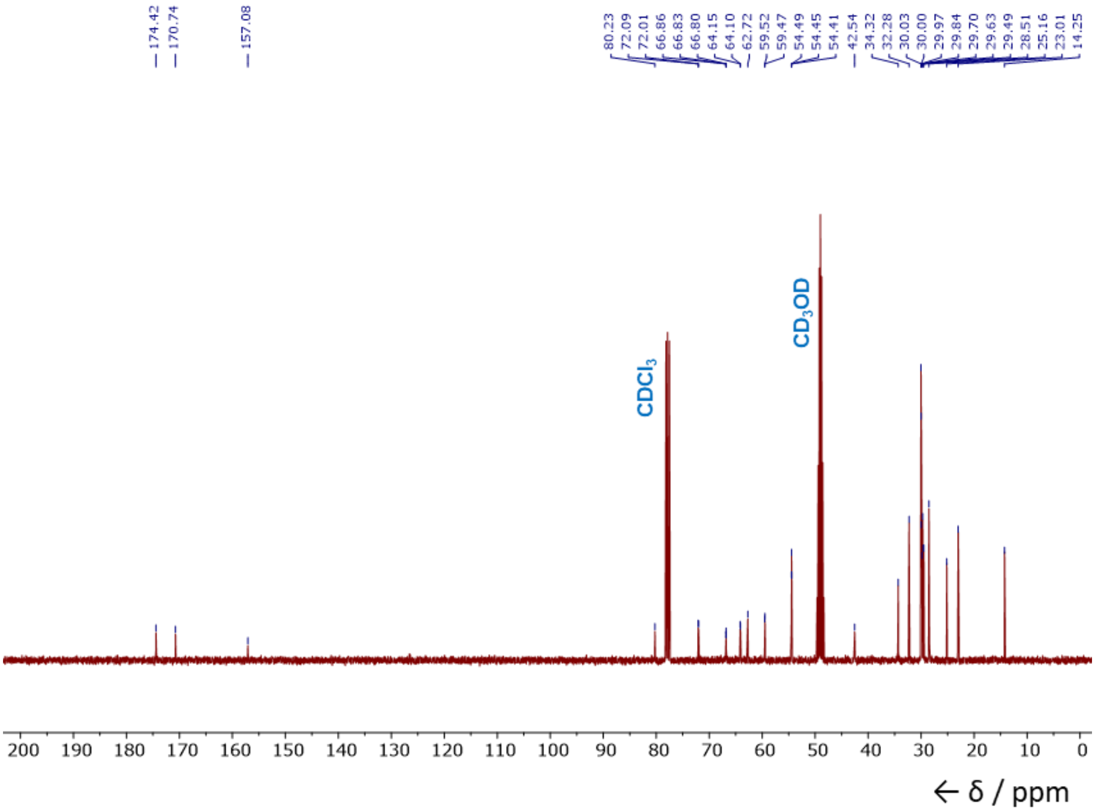

Figure S10. *^{13}C NMR spectrum of compound **12** (CDCl3:CD3OD (2:1), 101 MHz, 298 K).*

2.5 Fmoc-protected azobenzene lipid 2.

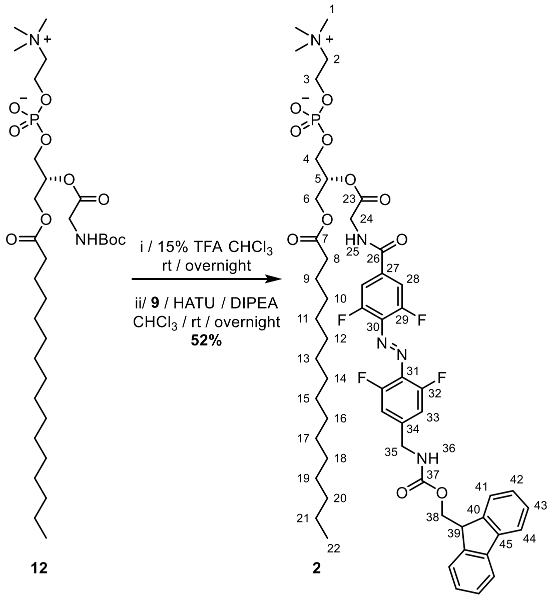

TFA (450 µL) was added to a stirred solution of (*R*)-1-palmitoyl-2-(*N*-boc-glycyl)-*sn*-glycero-3-phosphocholine **12** (100 mg, 0.15 mmol, 1 Equiv.) in anhydrous $CHCl_3$ (3 mL) under N_2 at rt. ^1H NMR confirmed complete deprotection after leaving the reaction overnight. The reaction mixture was concentrated and redissolved in anhydrous $CHCl_3$ (1 mL) and DIPEA (25 uL, 0.2 mmol, 1.3 Equiv.). Azobenzene **9** (126 mg, 0.23 mmol, 1.5 Equiv.), HATU (117 mg, 0.31 mmol, 2 Equiv.) and DIPEA (50 uL, 0.4 mmol, 2.6 Equiv.) were dissolved in anhydrous $CHCl_3$ (2 mL) and left to stir under N_2 for 10 min before being added to the lipid solution. The reaction mixture was then left to stir overnight. A drop of H_2O was added to the reaction mixture to quench the reaction and then the solvent was removed *in vacuo.* This crude material was purified by silica gel flash chromatography (0 - 2% H_2O / 25% MeOH / $CHCl_3$) to give the title compound as a red solid (87 mg, 0.8 mmol, 52%).

^{1}H NMR (500 MHz, $CDCl_3$:CD_3OD (2:1)) δ 7.74 (d, *J* = 7.3 Hz, 2H, H_{44}), 7.70 – 7.55 (m, 4H, $H_{41\,\&\,28}$), 7.37 (t, *J* = 7.3 Hz, 2H, H_{43}), 7.29 (t, *J* = 7.2 Hz, 2H, H_{42}), 6.98 (d, *J* = 10.3 Hz, 2H, H_{33}), 5.30 – 5.24 (m, 1H, H_5), 4.45 (d, *J* = 6.6 Hz, 2H, H_{38}), 4.42 – 4.33 (m, 2H, H_6), 4.31 (s, 2H, H_{35}), 4.23 (s, 2H, H_3), 4.22 – 4.15 (m, 3H, $H_{24\,\&\,39}$), 4.13 – 3.97 (m, 2H, H_4), 3.59 (s, 2H, H_2), 3.19 (s, 9H, H_1), 2.28 (q, *J* = 9.2 Hz, 2H, H_8), 1.61 – 1.51 (m, 2H, H_9), 1.34 – 1.17 (m, 24H, H_{10-21}), 0.84 (t, *J* = 6.9 Hz, 3H, H_{22}).

^{13}C {^{19}F} NMR (126 MHz, $CDCl_3$:CD_3OD (2:1)) δ 174.5 (C_7), 169.8 (C_{23}), 165.8 (C_{26}), 157.9 (C_{37}), 156.3, 155.5, 146.5 (C_{34}), 144.2, 141.8, 136.8 (C_{27}), 134.0, 130.6, 128.2 (C_{43}), 127.5 (C_{42}), 125.4 (C_{41}), 120.3 (C_{44}), 112.4 (C_{28}), 111.5 (C_{33}), 72.36 (d, *J* = 6.4 Hz) (C_5), 67.1 (C_{38}), 66.9 – 66.8 (m) (C_2), 64.2 (d, *J* = 4.6 Hz) (C_4), 62.6 (d, *J* = 5.8 Hz) (C_6), 59. 6 (d, *J* = 4.6 Hz) (C_3), 54.6 – 54.2 (m) (C_1), 47.7 (C_{39}), 44.2 (C_{35}), 42.1 (C_{24}), 34.3 (C_8), 32.3, 30.1, 30.0, 30.0, 30.0, 30.0, 29.7, 29.7, 29.7, 29.7, 29.5, 25.2, 23.0, 14.3.

^{19}F NMR (471 MHz, $CDCl_3$:CD_3OD (2:1)) δ -116.05 ($F_{29\text{ or }32}$), -116.73 ($F_{29\text{ or }32}$).

HRMS-ESI (m/z) Calculated for $C_{55}H_{71}N_5O_{11}F_4$ $[M+H]^+$, 1084.4818; found 1084.4816.

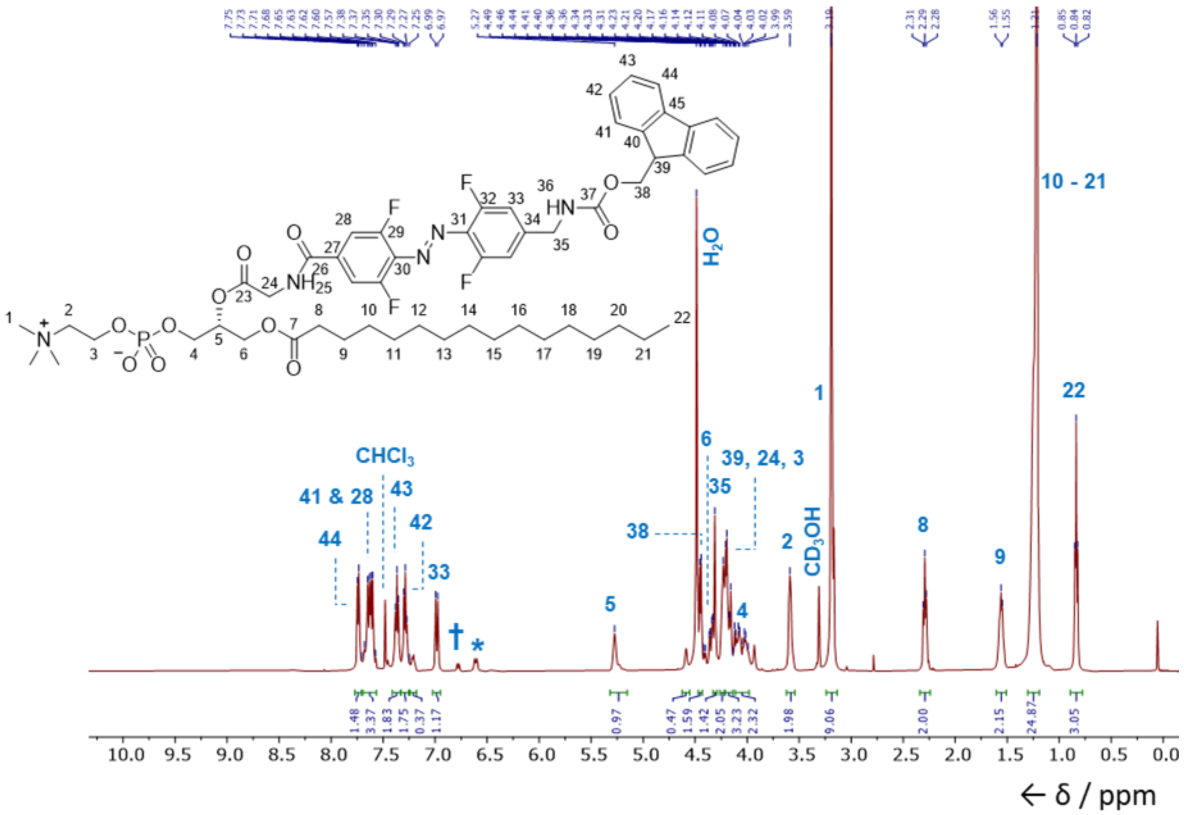

Figure S11. *^{1}H NMR spectrum of compound **2** ($CDCl_3$:CD_3OD (2:1), 500 MHz, 298 K). Rotameric signals are labelled with a blue asterisk (*) and signals from the minor Z-isomer are labelled with a blue cross (†).*

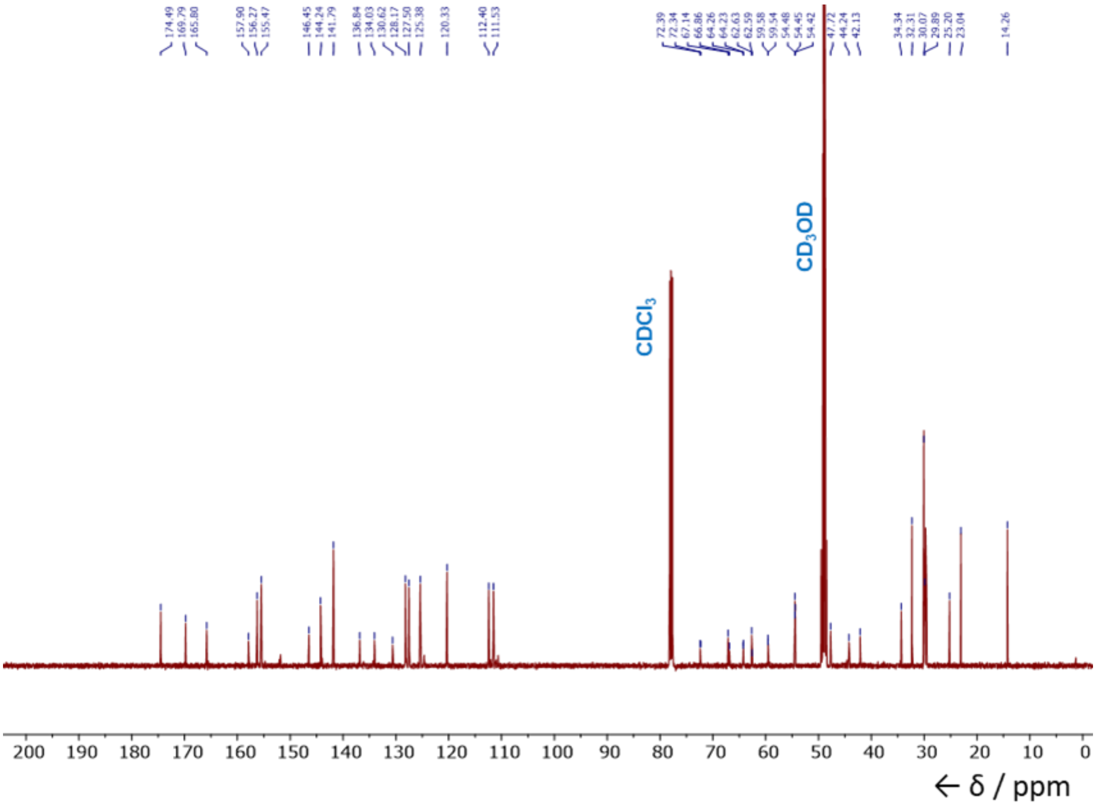

Figure S12. *^{13}C NMR spectrum of compound **2** ($CDCl_3$:CD_3OD (2:1), 126 MHz, 298 K).*

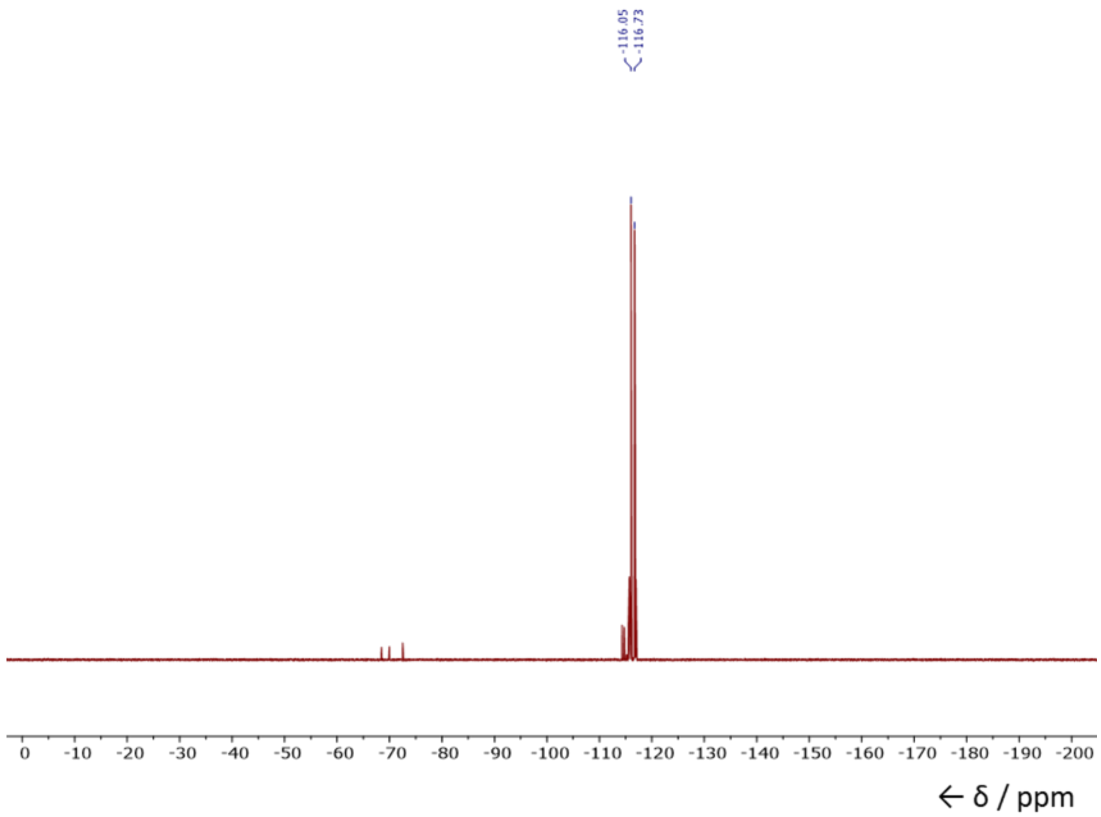

Figure S13. *^{19}F NMR spectrum of compound **2** ($CDCl_3$:CD_3OD (2:1), 377 MHz, 298 K).*

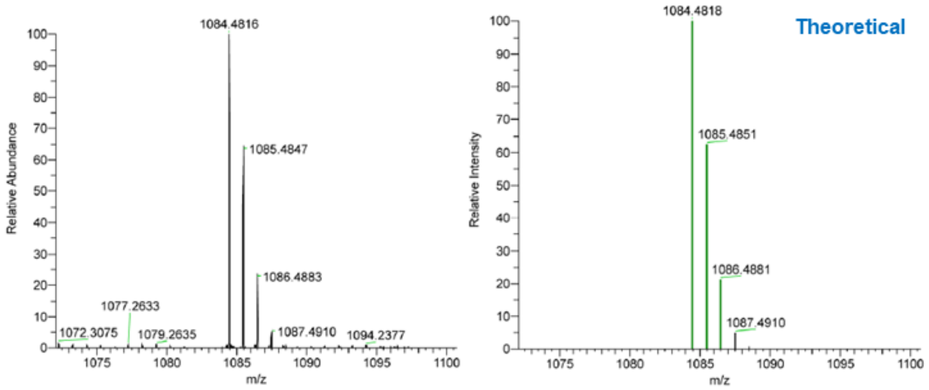

Figure S14. *HRMS spectrum of compound **2**. HRMS-ESI (m/z) calculated for $C_{55}H_{71}N_5O_{11}F_4$ $[M+H]^+$, 1084.4818; found 1084.4816.*

2.6 Short relay transporter 1.

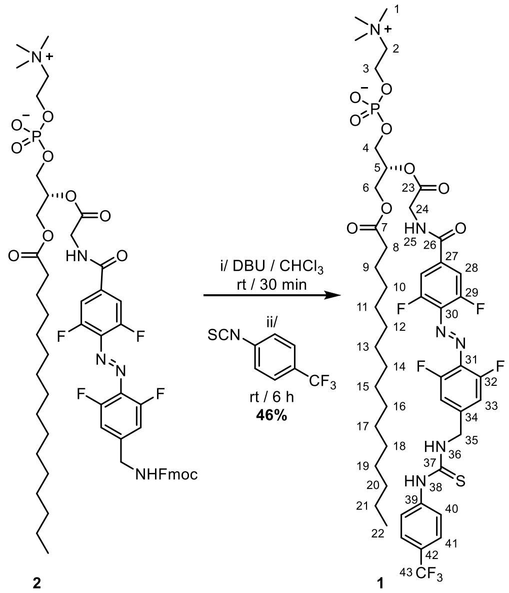

2 **1**

DBU (5.5 µL, 0.037 mmol, 2 Equiv.) was added to a stirred solution of Fmoc-protected azobenzene lipid **2** (20 mg, 0.019 mmol, 1 Equiv.) in anhydrous $CHCl_3$ (2 mL) and left to stir for 1 h under N_2. After complete deprotection of the Fmoc group, 4-(trifluoromethyl)phenyl isothiocyanate (5 mg, 0.025 mmol, 1.3 Equiv.) was added and left to stir for 6 h. The reaction mixture was concentrated and purified by silica gel flash chromatography (0 - 2% H_2O / 25% MeOH / $CHCl_3$) to give the title compound as a red solid (9 mg, 0.008 mmol, 46%).

^{1}H NMR (400 MHz, $CDCl_3$:CD_3OD (2:1)) δ 7.66 (d, *J* = 8.5 Hz, 2H, $H_{40\text{ or }41}$), 7.62 (d, *J* = 9.1 Hz, 2H, H_{28}), 7.57 (d, *J* = 8.5 Hz, 2H, $H_{40\text{ or }41}$), 7.10 (d, *J* = 10.2 Hz, 2H, H_{33}), 5.32 – 5.27 (m, 1H, H_5), 4.90 (s, 2H, H_{35}), 4.40 – 4.30 (m, 4H, $H_{3\,\&\,6}$), 4.26 – 4.07 (m, 4H, $H_{4\,\&\,24}$), 3.67 – 3.64 (m, 2H, H_2), 3.20 (s, 9H, H_1), 2.30 (t, *J* = 7.6 Hz, 2H, H_8), 1.59 – 1.53 (m, 2H, H_9), 1.32 – 1.17 (m, 24H, H_{10-21}), 0.88 – 0.80 (t, *J* = 6.7 Hz, 3H, H_{22}).

^{13}C NMR (151 MHz, $CDCl_3$:CD_3OD (2:1)) δ 182.7 (C_{37}), 174.5 (C_7), 169.8 (C_{23}), 166.0 (C_{26}), 157.5 – 156.3 (m), 155.1 (dd, J = 122.6, 4.5 Hz), 146.0 (C_{34}), 142.6, 136.8 (C_{27}), 134.2, 130.7, 127.1 (d, *J* = 32.9 Hz) (C_{43}), 126.4, 125.6, 112.4 (C_{28}), 111.9 (d, *J* = 21.2 Hz) (C_{33}), 72.0 (d, *J* = 6.6 Hz) (C_5), 66.7 – 66.5 (m) (C_2), 65.1 (d, *J* = 4.8 Hz) (C_4), 62.4 (C_6), 60.4 (d, *J* = 5.4 Hz) (C_3), 54.5 (C_1), 47.5 (C_{35}), 42.2 (C_{24}), 34.5 (C_8), 32.4, 30.1, 30.0, 30.0, 29.9, 29.9, 29.8, 29.7, 29.7, 29.6, 29.5, 25.2 (C_9), 23.1, 14.3 (C_{22}).

^{19}F NMR (377 MHz, $CDCl_3$:CD_3OD (2:1)) δ -62.75 (F_{43}), -120.01 ($F_{29\text{ or }32}$), -120.63 ($F_{29\text{ or }32}$).

HRMS-ESI (m/z) Calculated for $C_{48}H_{63}N_6O_9F_7PS$ $[M-H]^-$, 1063.4009; found 1063.3982.

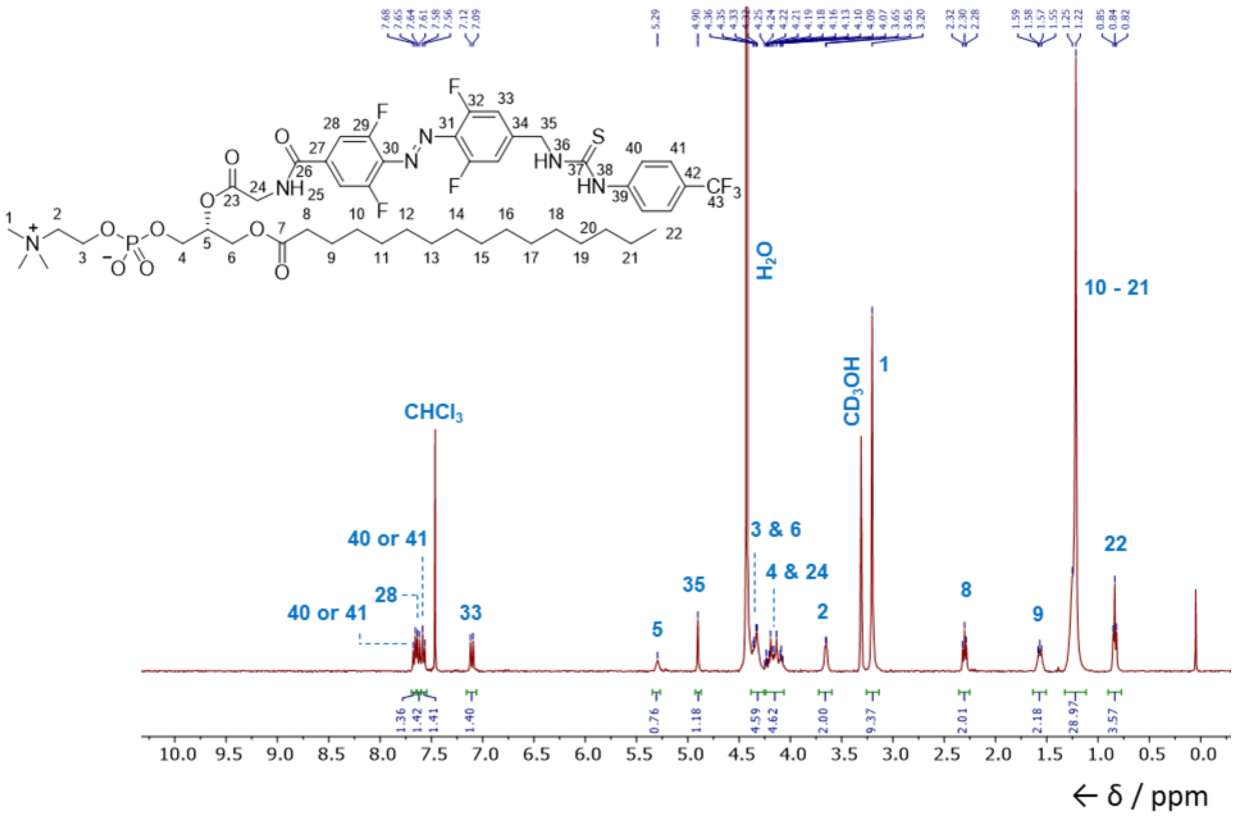

Figure S15. *^{1}H NMR spectrum of compound **1** (CDCl₃:CD₃OD (2:1), 400 MHz, 298 K).*

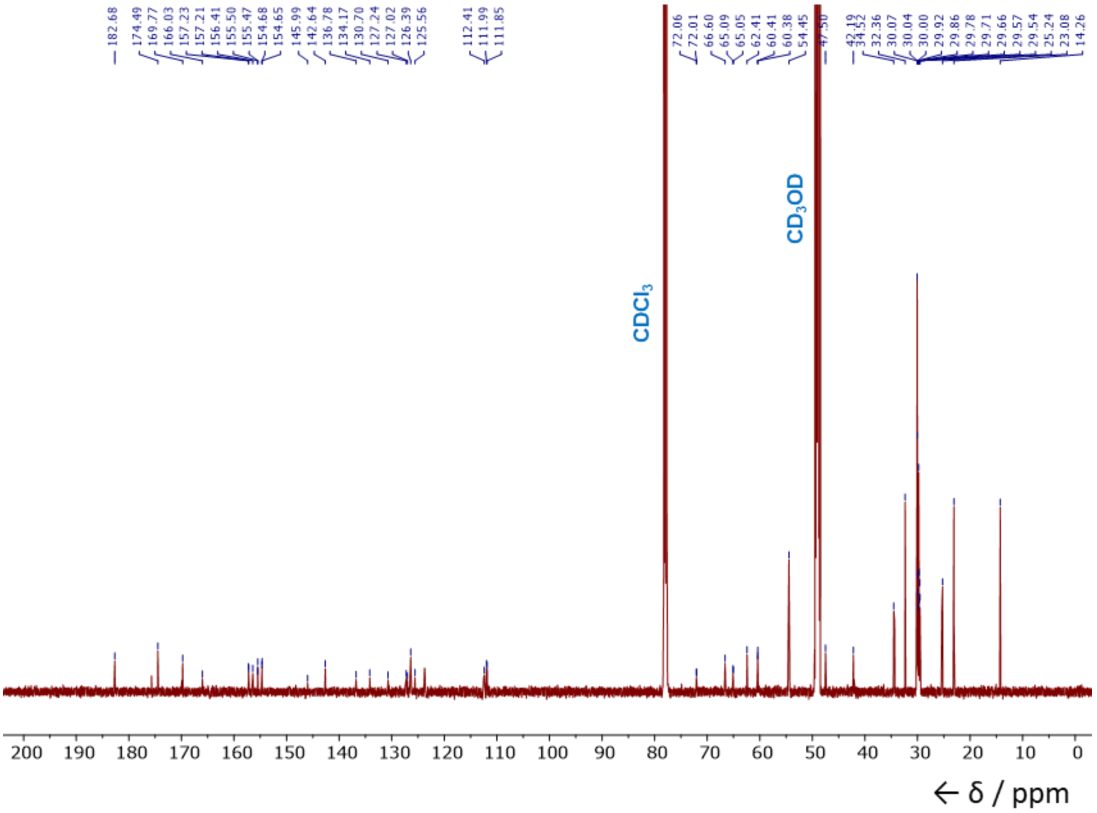

Figure S16. *^{13}C NMR spectrum of compound **1** (CDCl₃:CD₃OD (2:1), 151 MHz, 298 K).*

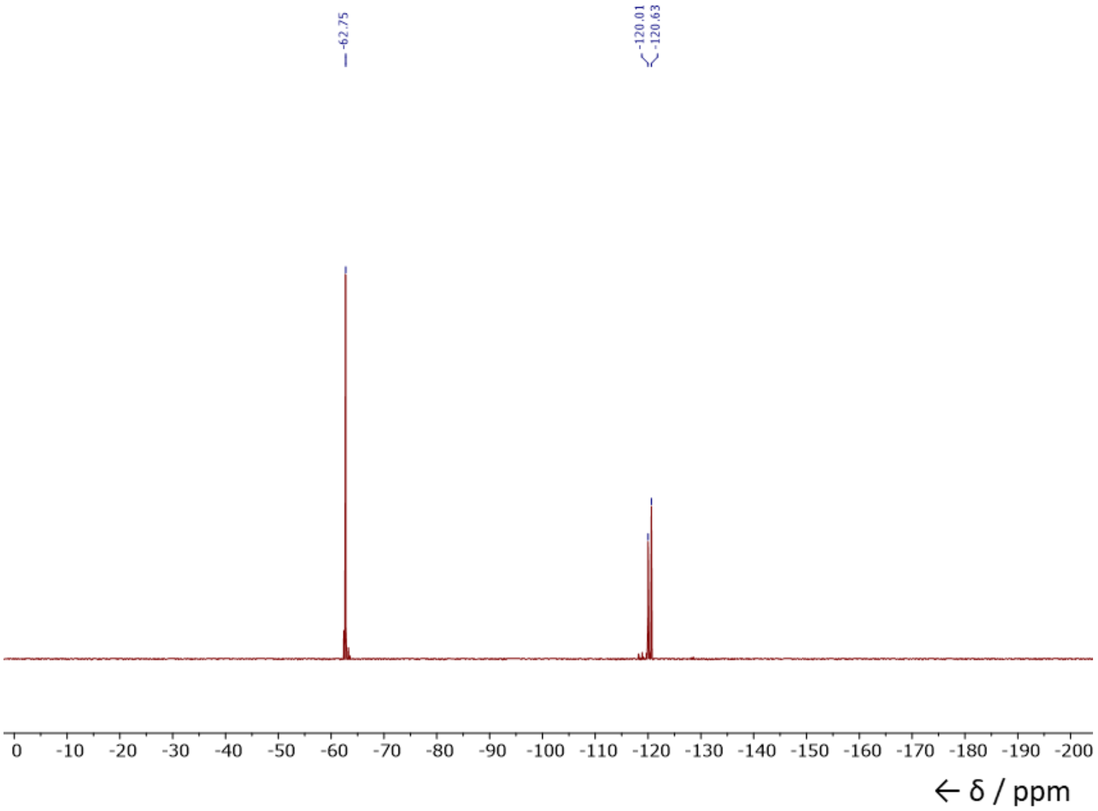

Figure S17. *^{19}F NMR spectrum of compound* ***1*** *($CDCl_3$:CD_3OD (2:1), 377 MHz, 298 K).*

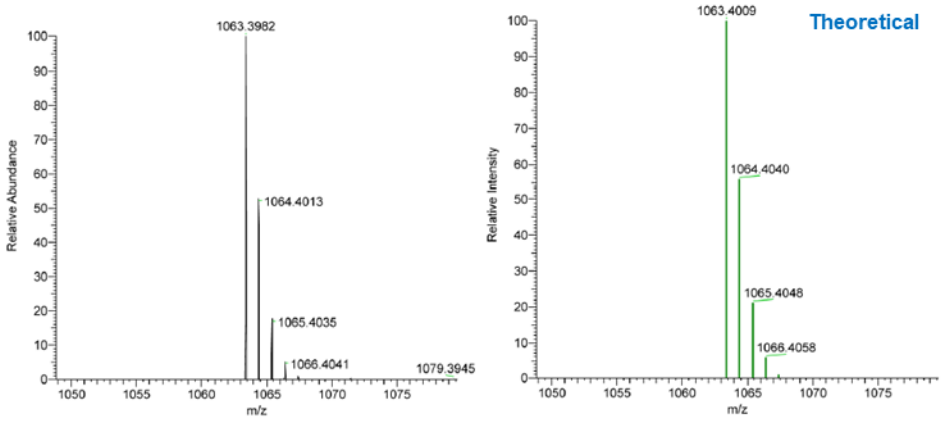

Figure S18. *HRMS spectrum of compound* ***1****. HRMS-ESI (m/z) calculated for $C_{48}H_{63}N_6O_9F_7PS$ [M-H]$^-$, 1063.4009; found 1063.3982.*

2.7 (*R*)-1-palmitoyl-2-(*N*-fmoc-glycyl)-*sn*-glycero-3-phosphocholine 19.

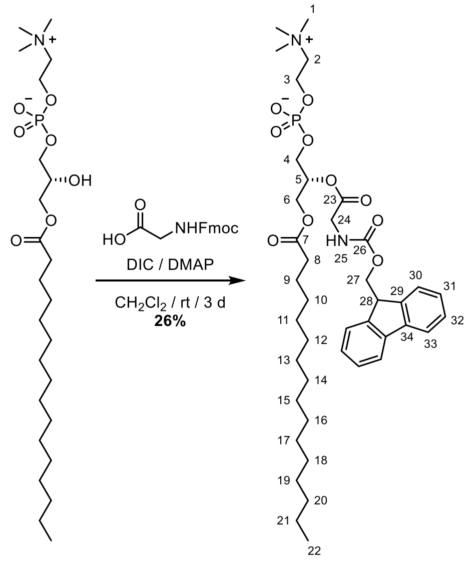

11 19

Fmoc-glycine (2.1 g, 7.1 mmol, 5 Equiv.), DIC (1.8 mL, 11.5 mmol, 8 Equiv.) and DMAP (250 mg, 2.1 mmol, 1.5 Equiv.) were added to a solution of 16:0 lyso PC **11** (700 mg, 1.4 mmol, 1 Equiv.) in anhydrous CH_2Cl_2 (100 mL) under N_2. After stirring the reaction mixture for 3 days, DOWEX 50 WX2-400 was added to remove DMAP. The solution was allowed to stir for 45 minutes. The reaction mixture was filtered, washed with $CHCl_3$:MeOH (1:1, 100 mL) and concentrated. The crude material was purified by silica gel flash chromatography (0 - 2% H_2O / 25% MeOH / $CHCl_3$) to afford the title compound as a white solid (240 mg, 0.37 mmol, 26%).

^{1}H NMR (400 MHz, $CDCl_3$:CD_3OD (2:1)) δ 7.74 (d, *J* = 7.5 Hz, 2H, H_{33}), 7.61 (t, *J* = 6.6 Hz, 2H, H_{30}), 7.37 (t, *J* = 7.4 Hz, 2H, H_{32}), 7.28 (t, *J* = 7.3 Hz, 2H, H_{31}), 5.28 – 5.22 (m, 1H, H_5), 4.33 – 4.30 (m, 3H, $H_{6\,\&\,27}$), 4.23 – 4.12 (m, 4H, $H_{3,\,6\,\&\,28}$), 4.05 – 3.96 (m, 2H, H_4), 3.93 (d, *J* = 3.7 Hz, 2H, H_{24}), 3.57 – 3.49 (m, 2H, H_2), 3.13 (s, 9H, H_1), 2.27 (t, *J* = 7.6 Hz, 2H, H_8), 1.59 – 1.49 (m, 2H, H_9), 1.29 – 1.17 (m, 24H, $H_{10\text{-}21}$), 0.84 (t, *J* = 6.8 Hz, 3H, H_{22}).

^{13}C NMR (101 MHz, $CDCl_3$:CD_3OD (2:1)) δ 174.5 (C_7), 170.5 (C_{23}), 157.8 (C_{26}), 144.2 (d, *J* = 10.5 Hz), 141.7, 128.1 (C_{32}), 127.5 (C_{31}), 125.5 (d, *J* = 5.9 Hz) (C_{30}), 120.3 (C_{33}), 72.2 (d, *J* = 7.0 Hz) (C_5), 67.5 (C_{27}), 66.9 – 66.6 (m) (C_2), 64.2 (d, *J* = 4.8 Hz) (C_4), 62.7 (C_6), 59.5 (d, *J* = 5.1 Hz) (C_3), 54.5 – 54.3 (m) (C_1), 47.5 (C_{28}), 42.8 (C_{24}), 34.3 (C_8), 32.3, 30.0, 30.0, 30.0, 30.0, 30.0, 30.0, 29.8, 29.7, 29.6, 29.5, 25.1 (C_9), 23.0, 14.3 (C_{22}).

HRMS-ESI (m/z) Calculated for $C_{41}H_{64}O_{10}N_2P$ $[M+H]^+$, 775.4293; found 775.4291.

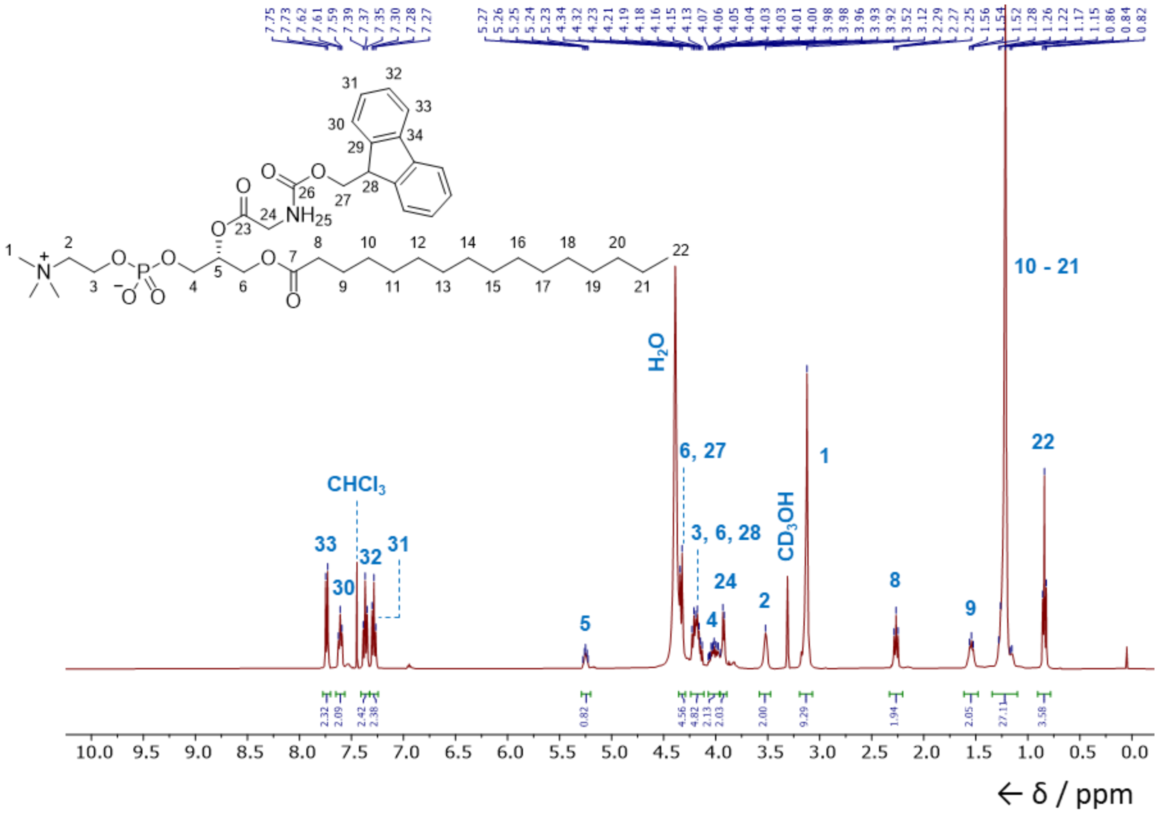

Figure S19. *^{1}H NMR spectrum of compound* ***19*** *($CDCl_3$:CD_3OD (2:1), 400 MHz, 298 K).*

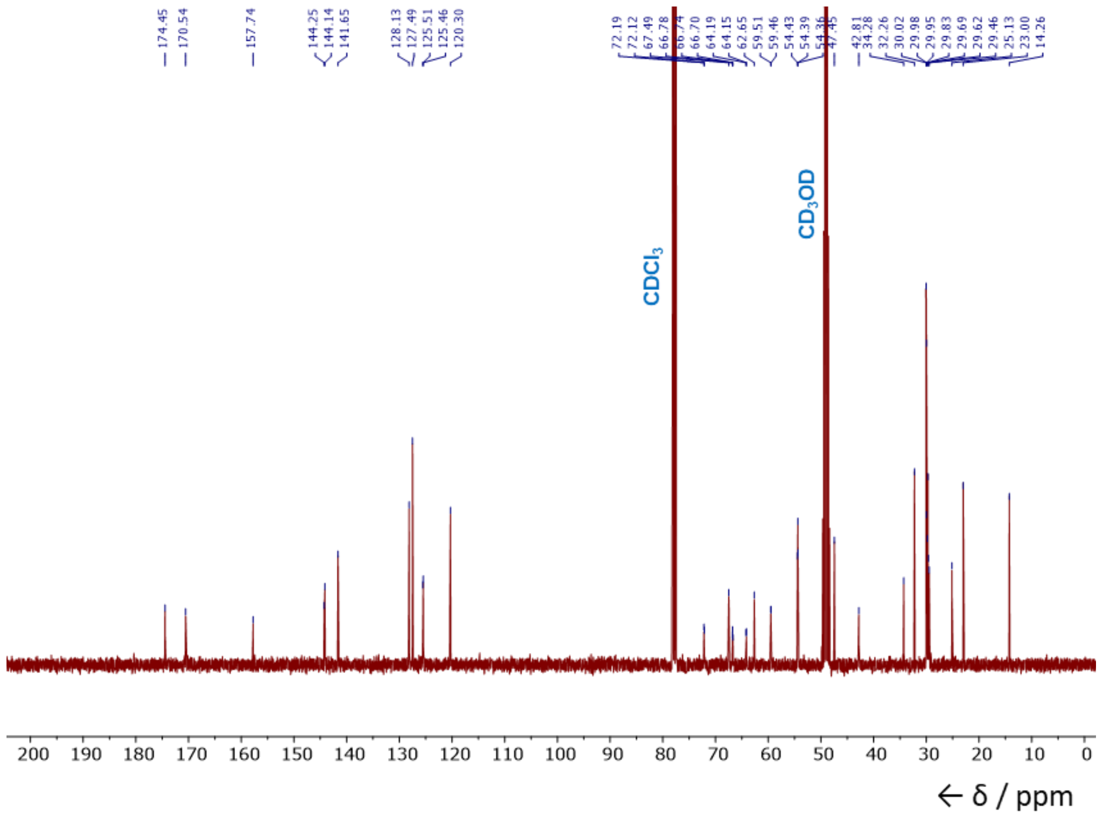

Figure S20. *^{13}C NMR spectrum of compound **19** (CDCl₃:CD₃OD (2:1)), 101 MHz, 298 K).*

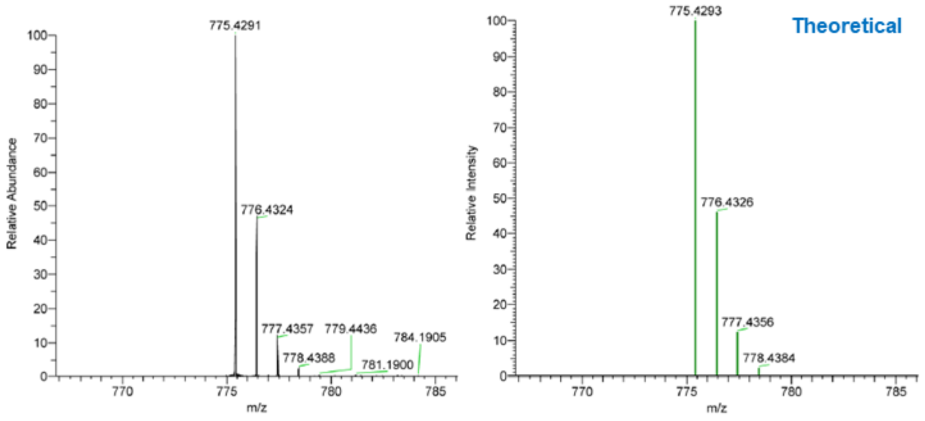

Figure S21. *HRMS spectrum of compound **19**. HRMS-ESI (m/z) calculated for $C_{41}H_{64}O_{10}N_2P$ $[M+H]^+$, 775.4293; found 775.4291.*

2.8 Mtt-protected medium azobenzene lipid 20.

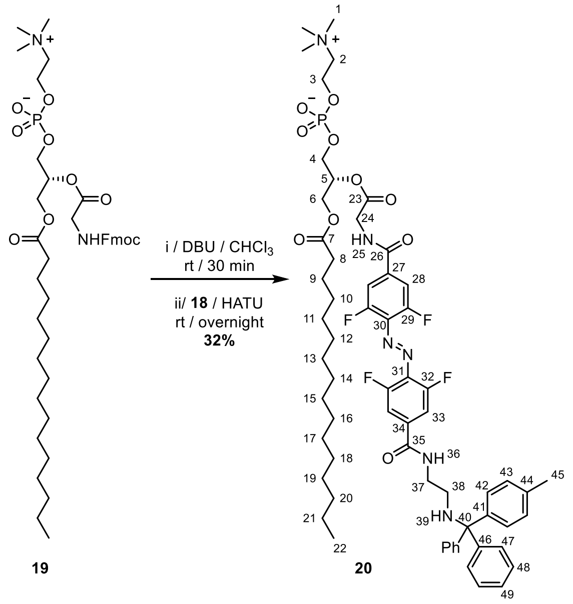

DBU (25 µL, 0.17 mmol, 2 Equiv.) was added to a stirred solution of **19** (65 mg, 0.84 mmol, 1 Equiv.) in anhydrous $CHCl_3$ (1 mL) and left to stir for 1 h under N_2. Compound **18** (100 mg, 0.13 mmol, 1.6 Equiv.) and HATU (64 mg, 0.17 mmol, 2 Equiv.) were dissolved in anhydrous $CHCl_3$ (1 mL) and left to stir for 10 min before being added to the reaction mixture, which was left to stir overnight under N_2. The reaction mixture was concentrated and purified by silica gel flash chromatography (0 - 2% H_2O / 25% MeOH / $CHCl_3$) to afford the title compound as a red solid (32 mg, 0.027 mmol, 32%).

^{1}H NMR (400 MHz, $CDCl_3$:CD_3OD (2:1)) δ 7.66 (d, J = 9.2 Hz, 2H, $H_{28\text{ or }33}$), 7.58 (d, J = 9.2 Hz, 2H $H_{28\text{ or }33}$), 7.44 – 7.36 (m, 4H, H_{47}), 7.29 (d, *J* = 8.3 Hz, 2H, H_{42}), 7.27 – 7.20 (m, 4H, H_{48}), 7.16 (q, *J* = 6.2 Hz, 2H, H_{49}), 7.05 (d, *J* = 8.1 Hz, 2H, H_{43}), 5.34 – 5.19 (m, 1H, H_5), 4.37 – 4.28 (m, 1H, H_6), 4.26 – 4.15 (m, 4H, $H_{3,\,6\,\&\,24}$), 4.14 – 3.96 (m, 3H, $H_{3\,\&\,4}$), 3.58 (s, 2H, H_2), 3.51 (t, *J* = 6.0 Hz, 2H, H_{37}), 3.18 (s, 9H, H_1), 2.45 (t, *J* = 5.8 Hz, 2H, H_{38}), 2.33 – 2.27 (m, 2H, H_8), 2.27 (s, 3H, H_{45}), 1.59 – 1.52 (m, 2H, H_9), 1.29 – 1.78 (m, 24H, H_{10-21}), 0.83 (t, *J* = 6.8 Hz, 3H, H_{22}).

^{13}C {^{19}F} NMR (126 MHz, $CDCl_3$:CD_3OD (2:1)) δ 174.4 (C_7), 169.7 (C_{23}), 165.5 (C_{26}), 165.5 (C_{35}), 155.6, 155. 6, 151.8, 151.7, 138.3, 137.6, 136.7, 133.6, 133.4, 129.0, 128.9, 128.3, 128.1, 127.0, 112.4 ($C_{28\text{ or }33}$), 112.2 ($C_{28\text{ or }33}$), 72.3(C_{40}), 72.3 (d, *J* = 6.5 Hz) (C_5), 66.9 – 66.7 (m) (C_2), 64.2 (d, *J* = 4.6 Hz) (C_4), 62.5 (C_6), 59.5 (d, *J* = 4.5 Hz) (C_3), 54.5 – 54.4 (m) (C_1), 44.0 (C_{38}), 42.1 (C_{24}), 40.9 (C_{37}), 34.3 (C_8), 32.2, 30.0, 30.0, 30.0, 30.0, 29.8, 29.8, 29.8, 29.7, 29.6, 29.5, 25.1 (C_9), 23.0, 21.0 (C_{45}), 14.2 (C_{22}).

^{19}F NMR (471 MHz, $CDCl_3$:CD_3OD (2:1)) δ -115.73 ($F_{29\text{ or }32}$), -115.81 ($F_{29\text{ or }32}$).

HRMS-ESI (m/z) Calculated for $C_{62}H_{80}O_{10}N_6F_4P$ $[M+H]^+$, 1175.5604; found 1175.5562.

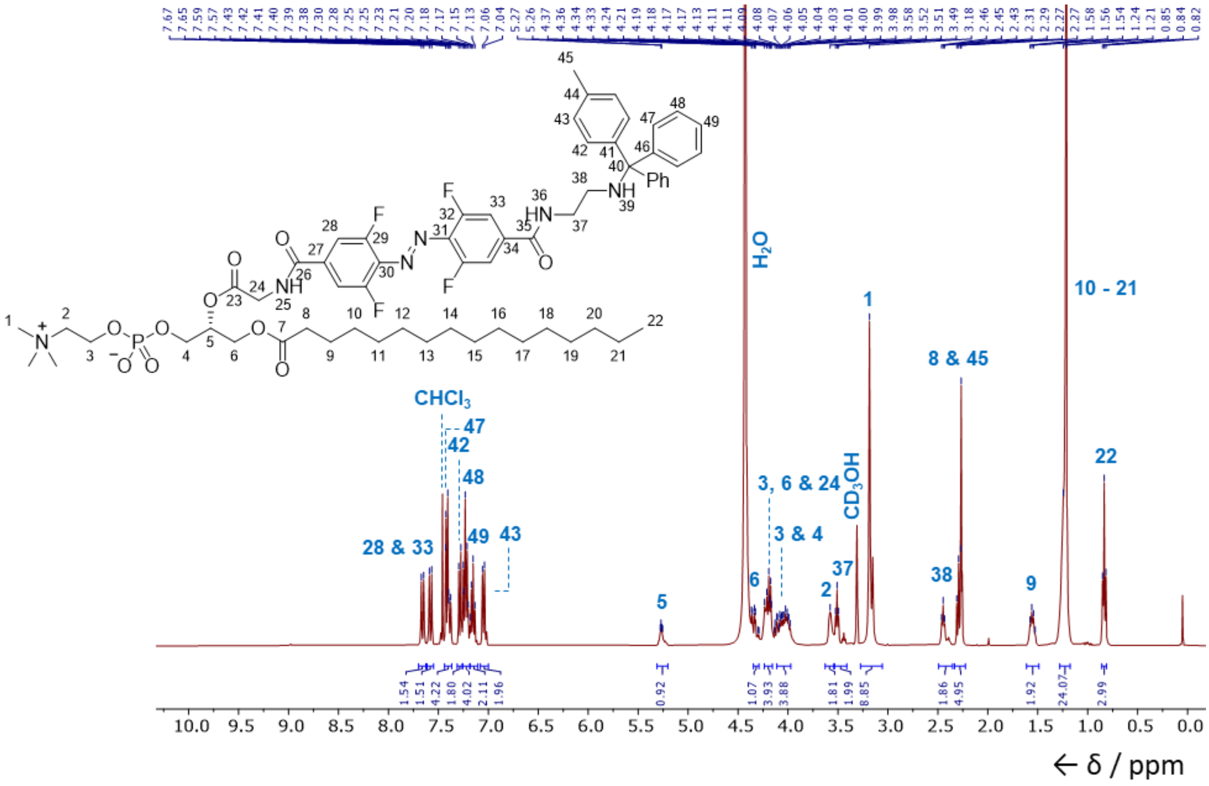

Figure S22. *^{1}H NMR spectrum of compound **20** (CDCl3:CD3OD (2:1), 400 MHz, 298 K).*

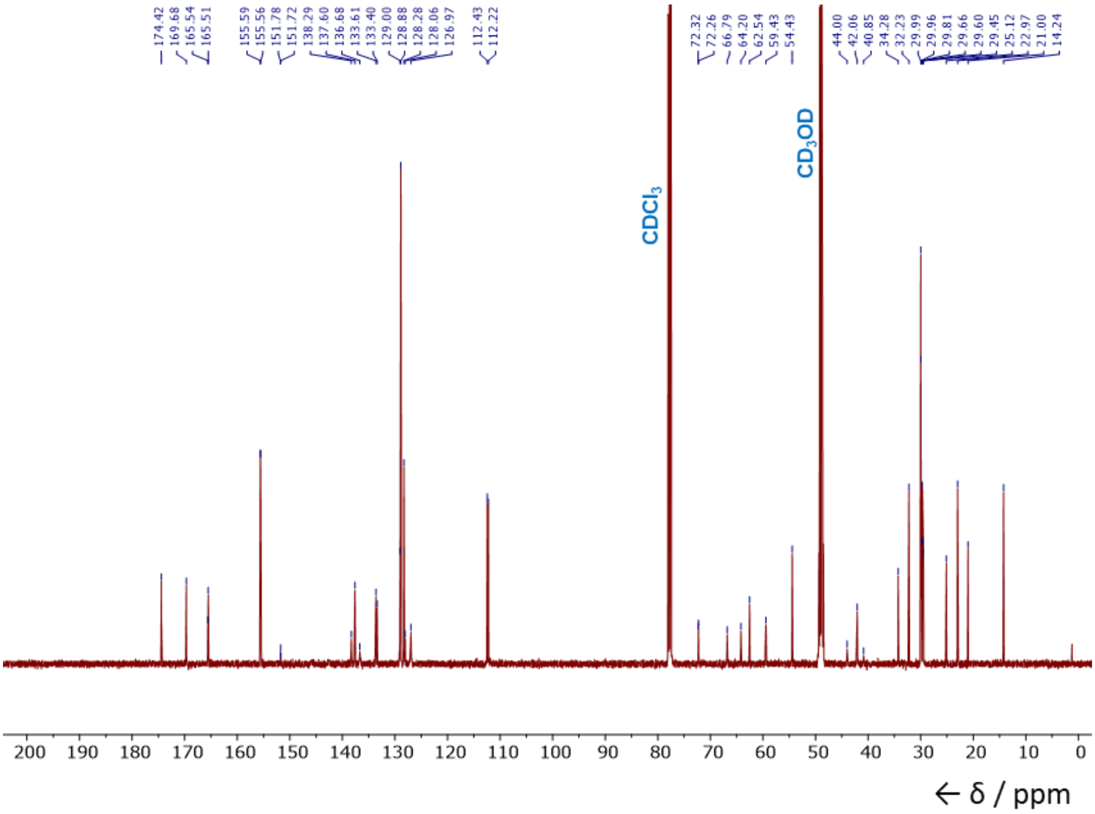

Figure S23. *^{13}C {^{19}F} NMR spectrum of compound **20** (CDCl3:CD3OD (2:1)), 126 MHz, 298 K).*

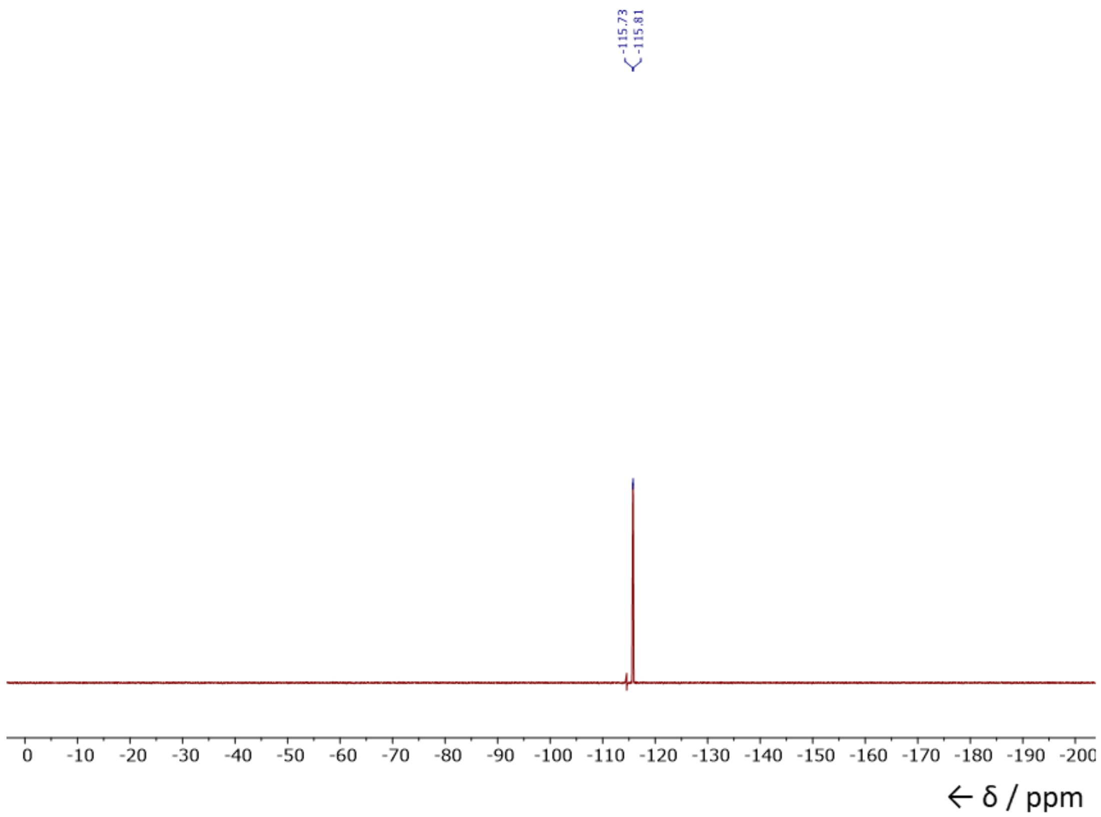

Figure S24. *^{19}F NMR spectrum of compound **20** ($CDCl_3$:CD_3OD (2:1), 471 MHz, 298 K).*

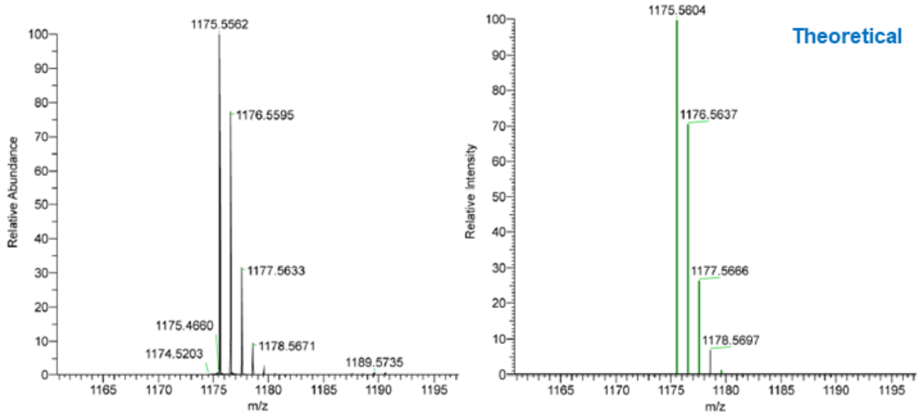

Figure S25. *HRMS spectrum of compound **20**. HRMS-ESI (m/z) calculated for $C_{62}H_{80}O_{10}N_6F_4P$ [M+H]$^+$, 1175.5604; found 1175.5562.*

2.9 Medium relay transporter 3.

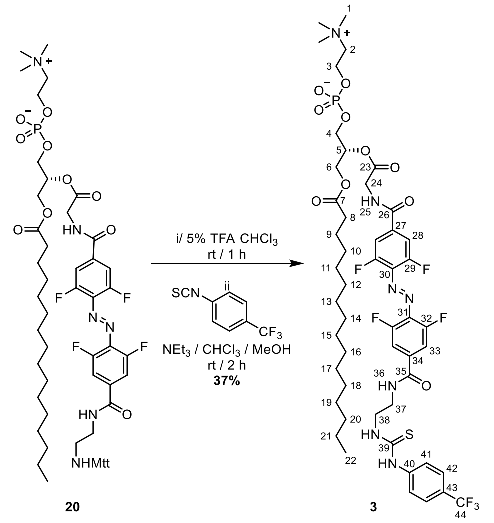

Mtt-protected medium azobenzene lipid **20** (17 mg, 0.015 mmol, 1 Equiv.) was dissolved in anhydrous $CHCl_3$ (1 mL) and TFA (50 µL, 5% v/v) and left to stir at rt for 1 h under N_2. After complete deprotection, MeOH (1 mL) was added and the reaction mixture was concentrated. The residue was dissolved in anhydrous $CHCl_3$:MeOH (1:1, 2 mL) and NEt_3 (50 µL). 4-(Trifluoromethyl)phenyl isothiocyanate (6 mg, 0.03 mmol, 2 Equiv.) was added to the reaction mixture and left to stir at rt for 4 h under N_2. The reaction mixture was then concentrated and purified by silica gel flash chromatography (0 - 3% H_2O / 25% MeOH / $CHCl_3$) to afford the title compound as a red solid (6 mg, 0.005 mmol, 37%).

^{1}H NMR (400 MHz, $CDCl_3$:CD_3OD (2:1)) δ 7.68 – 7.60 (m, 4H, $H_{28\ \&\ 33}$), 7.56 (s, 4H, $H_{41\ \&\ 42}$), 5.32 – 5.28 (m, 1H, H_5), 4.40 – 4.27 (m, 4H, $H_{3\ \&\ 6}$), 4.26 – 4.08 (m, 4H, $H_{4\ \&\ 24}$), 3.91 – 3.86 (m, 2H, H_{38}), 3.70 – 3.58 (m, 4H, $H_{2\ \&\ 37}$), 3.21 (s, 9H, H_1), 2.31 (t, *J* = 7.4 Hz, 2H, H_8), 1.62 – 1.53 (m, 2H, H_9), 1.30 – 1.84 (m, 24H, H_{10-21}), 0.91 – 0.76 (m, 3H, H_{22}).

^{13}C NMR (151 MHz, $CDCl_3$:CD_3OD (2:1)) δ 175.7, 174.5 (C_7), 170.9, 169.8, 166.1 (C_{39}), 156.6, 154.9, 142.3, 138.2, 137.6, 133.9, 133.6, 127.2, 126.5 ($C_{41\ or\ 42}$), 125.5, 124.0 ($C_{41\ or\ 42}$), 112.5, 112.4, 72.0 (C_5), 66.6 (C_2), 65.2 (C_4), 62.4 (C_6), 60.6 (C_3), 54.5 (C_1), 44.2 (C_{38}), 42.2 (C_{24}), 40.9 (C_{37}), 34.5 (C_8), 32.4, 30.1, 30.1, 30.0, 29.9, 29.9, 29.8, 29.7, 29.7, 29.6, 29.6, 25.4 (C_9), 23.1, 14.3 (C_{22}).

^{19}F NMR (377 MHz, $CDCl_3$:CD_3OD (2:1)) δ -62.79 (C_{44}), -119.73 ($C_{28\ or\ 33}$), -119.78 ($C_{28\ or\ 33}$).

HRMS-ESI (m/z) Calculated for $C_{50}H_{68}O_{10}N_7F_7PS$ $[M+H]^+$, 1122.4369; found 1122.4349.

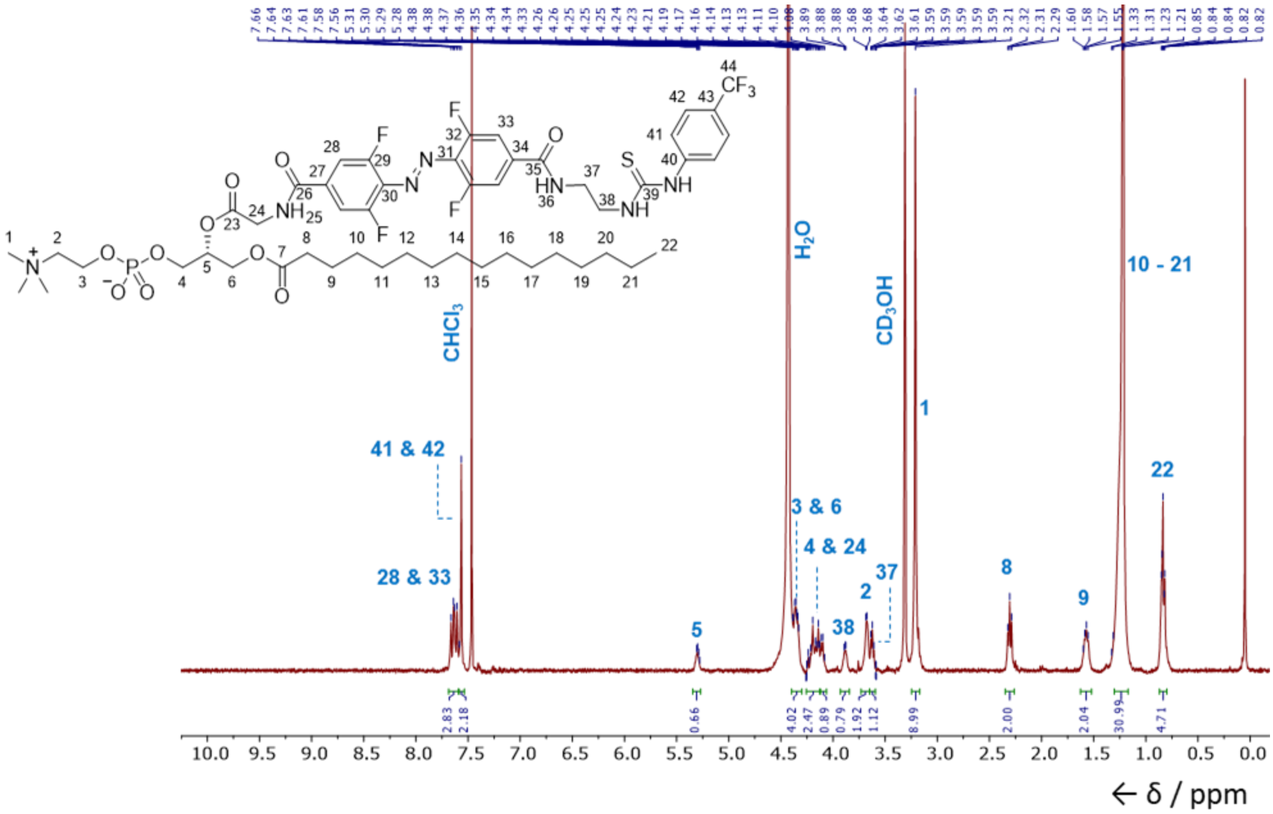

Figure S26. *^{1}H NMR spectrum of compound **3** ($CDCl_3$:CD_3OD (2:1), 400 MHz, 298 K).*

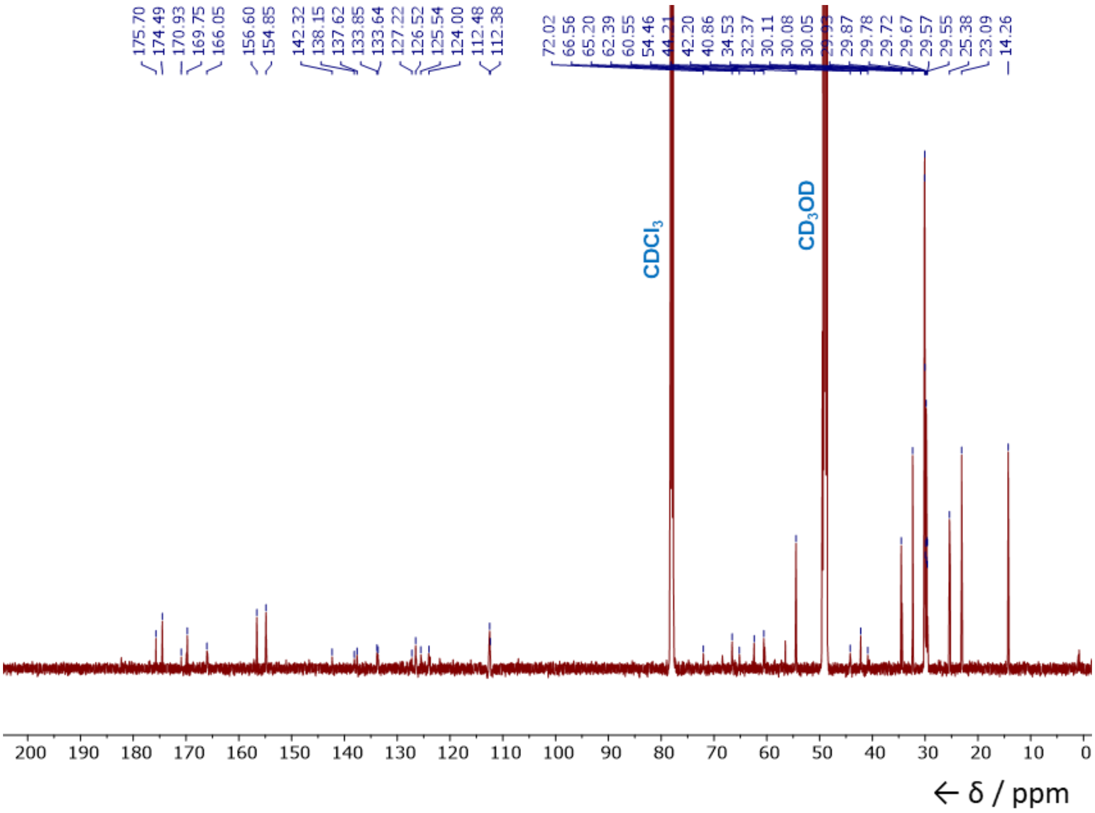

Figure S27. *^{13}C NMR spectrum of compound **3** ($CDCl_3$:CD_3OD (2:1)), 151 MHz, 298 K).*

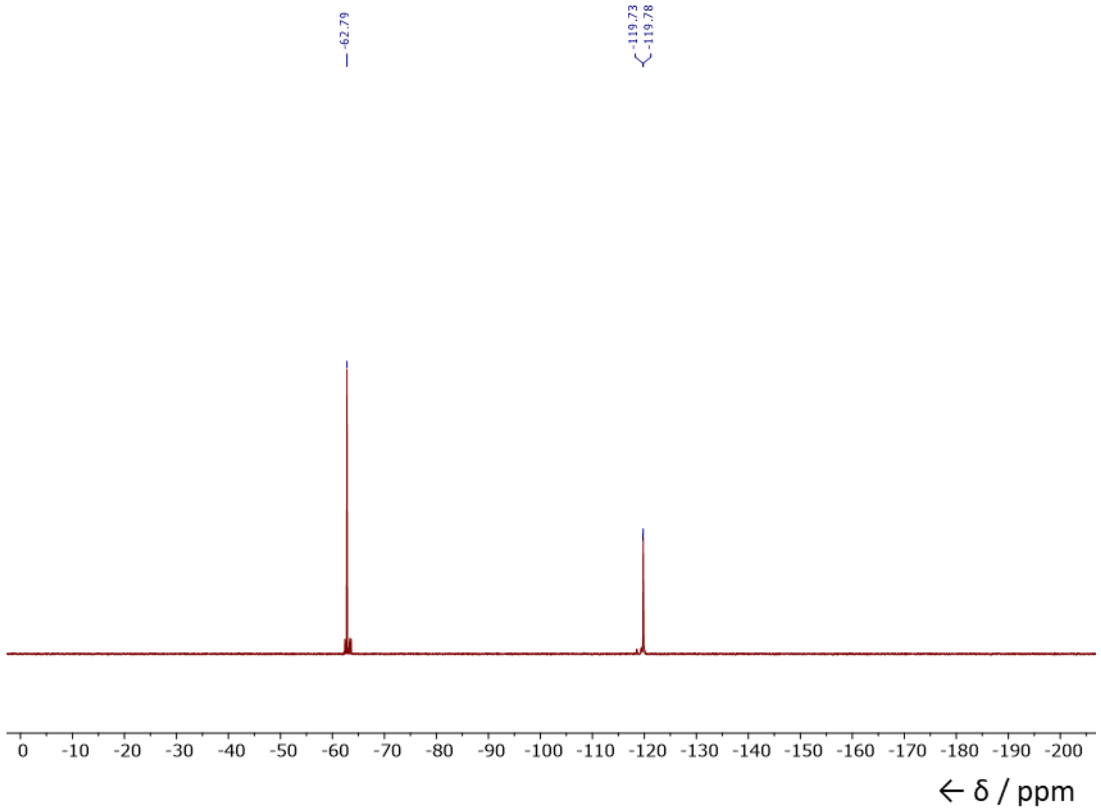

Figure S28. *^{19}F NMR spectrum of compound* ***3*** *($CDCl_3$:CD_3OD (2:1), 377 MHz, 298 K).*

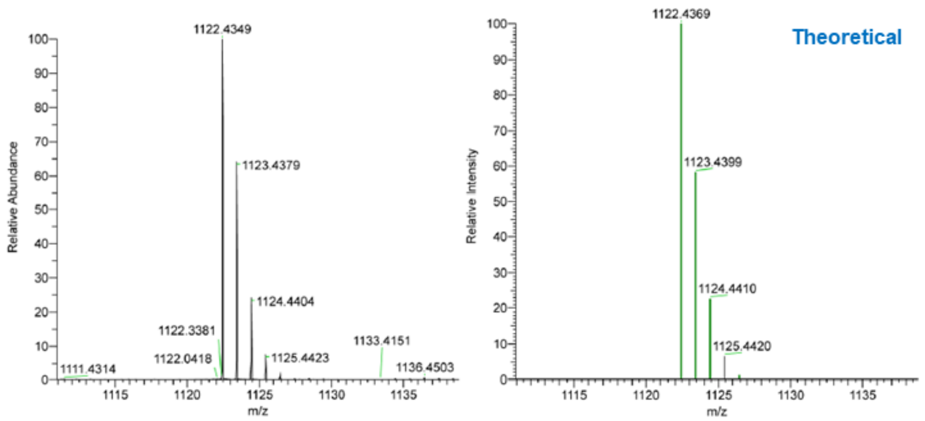

Figure S29. *HRMS spectrum of compound* ***3****. HRMS-ESI (m/z) calculated for $C_{50}H_{68}O_{10}N_7F_7PS$ $[M+H]^+$, 1122.4369; found 1122.4349.*

2.10 NHS ester azobenzene 24:

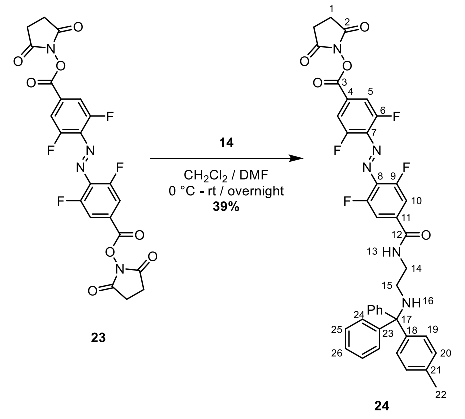

Amine **14** (30 mg, 0.095 mmols, 1.02 Equiv.) in anhydrous CH_2Cl_2 (2 mL) was added to a stirred solution of **23** (50 mg, 0.093 mmols, 1 Equiv.) in anhydrous DMF (10 mL) at 0 °C *via* syringe pump over 2h, and then allowed to react overnight at rt. After completion, the mixture was diluted with H_2O (30 mL) and extracted with EtOAc (3 × 30 mL). The organic layers were combined washed with H_2O (5 × 20 mL), brine (1 × 20 mL) and dried ($MgSO_4$). The solvent was removed *in vacuo* to give a crude mixture which was purified by silica gel flash chromatography (EtOAc/hexane, 1:1) to give the title compound as a red solid. (26.5 mg, 0.036 mmol, 39%.)

^{1}H NMR (600 MHz, $CDCl_3$) δ 7.85 (d, *J* = 8.1 Hz, 2H, $H_{5\text{ or }10}$), 7.59 – 7.51 (m, 2H, $H_{5\text{ or }10}$), 7.47 (d, *J* = 7.7 Hz, 4H, H_{24}), 7.35 (d, *J* = 8.0 Hz, 2H, H_{19}), 7.28 (t, *J* = 7.6 Hz, 4H, H_{25}), 7.22 (t, *J* = 7.3 Hz, 2H, H_{26}), 7.10 (d, *J* = 8.2 Hz, 2H, H_{20}), 6.98 (s, 1H, H_{13}), 3.61 (brs, 2H, H_{14}), 2.93 (s, 4H, H_1), 2.59 (brs, 2H, H_{15}), 2.30 (s, 3H, H_{22}), 2.27 (s, 1H, H_{16}).

^{13}C NMR (151 MHz, $CDCl_3$) δ 168.8 (C_2), 164.3 ($C_{3\text{ or }12}$), 159.6 ($C_{3\text{ or }12}$), 155.7 (dd, *J* = 264.8, 3.5 Hz), 155.1 (dd, *J* = 263.6, 3.8 Hz), 151.7 (C_{18}), 147.2 (C_{23}), 135.9 (C_{21}), 135.9 (t, *J* = 10.5 Hz), 133.12 (t, *J* = 9.9 Hz), 129.0 (C_{20}), 128.8 (t, *J* = 7.3 Hz), 128.7 (C_{24}), 128.3 (C_{25}), 127.7 (t, *J* = 9.6 Hz), 127.1 (C_{26}), 125.7 (C_{19}), 115.2 (dd, *J* = 21.9, 4.6 Hz), 111.9 (d, *J* = 19.9 Hz), 34.4 (C_{14}), 30.5 (C_{15}), 29.8 (C_{17}), 25.8 (C_1), 21.1 (C_{22}).

^{19}F NMR (565 MHz, $CDCl_3$) δ -117.73 ($F_{6\text{ or }9}$), -118.30 ($F_{6\text{ or }9}$).

HRMS-ESI (m/z) Calculated for $C_{40}H_{31}O_5N_5F_4Na$ $[M+Na]^+$, 760.2178; found 760.2181.

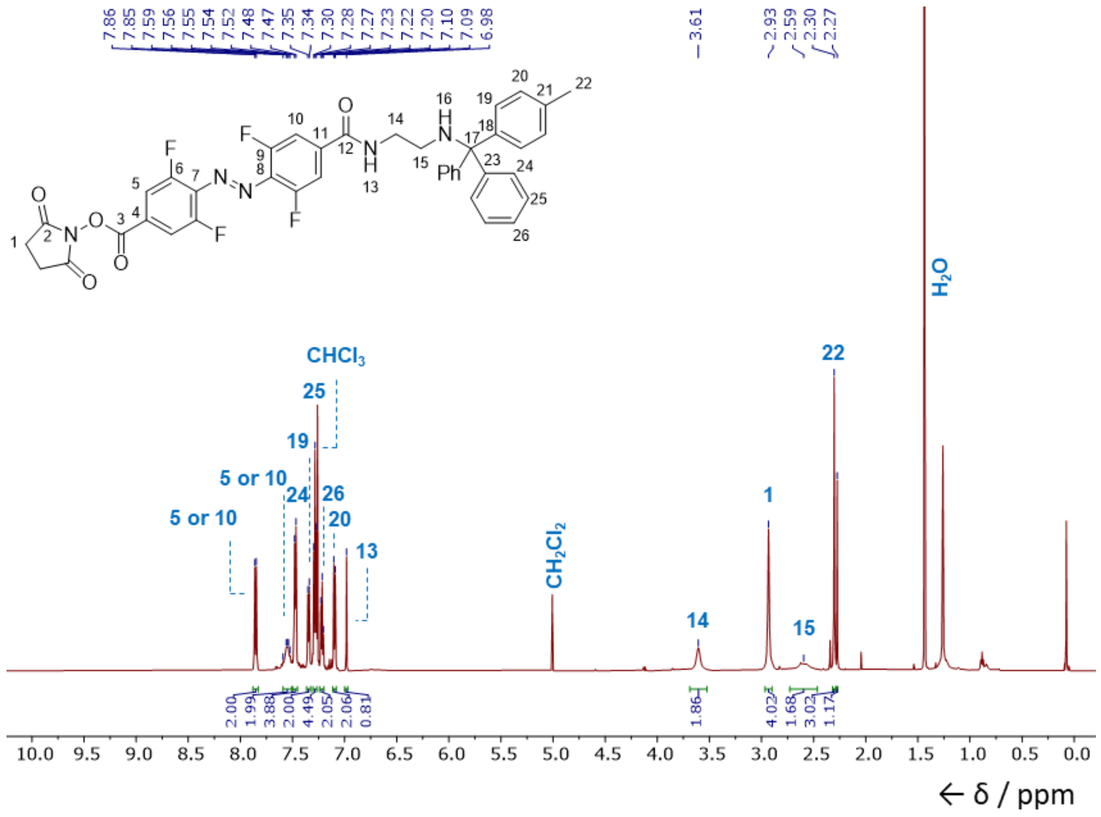

Figure S30. *^{1}H NMR spectrum of compound **24** ($CDCl_3$, 600 MHz, 298 K).*

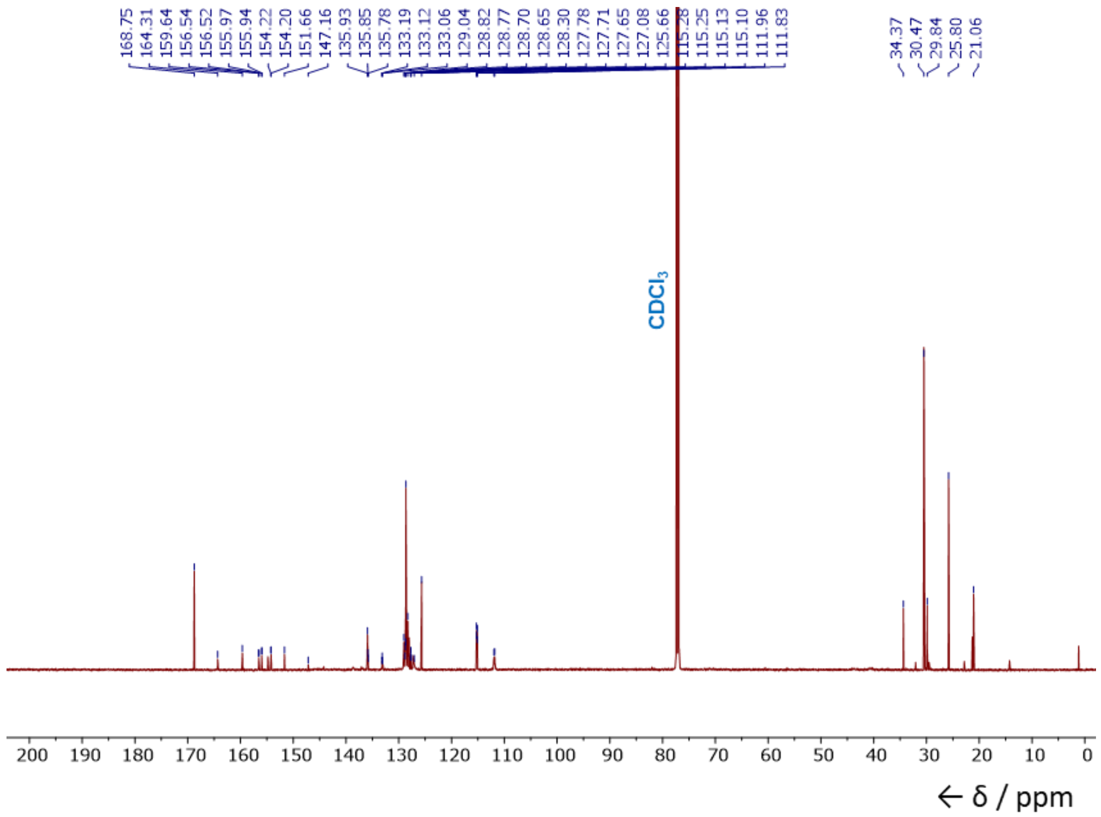

Figure S31. *^{13}C NMR spectrum of compound **24** ($CDCl_3$, 151 MHz, 298 K).*

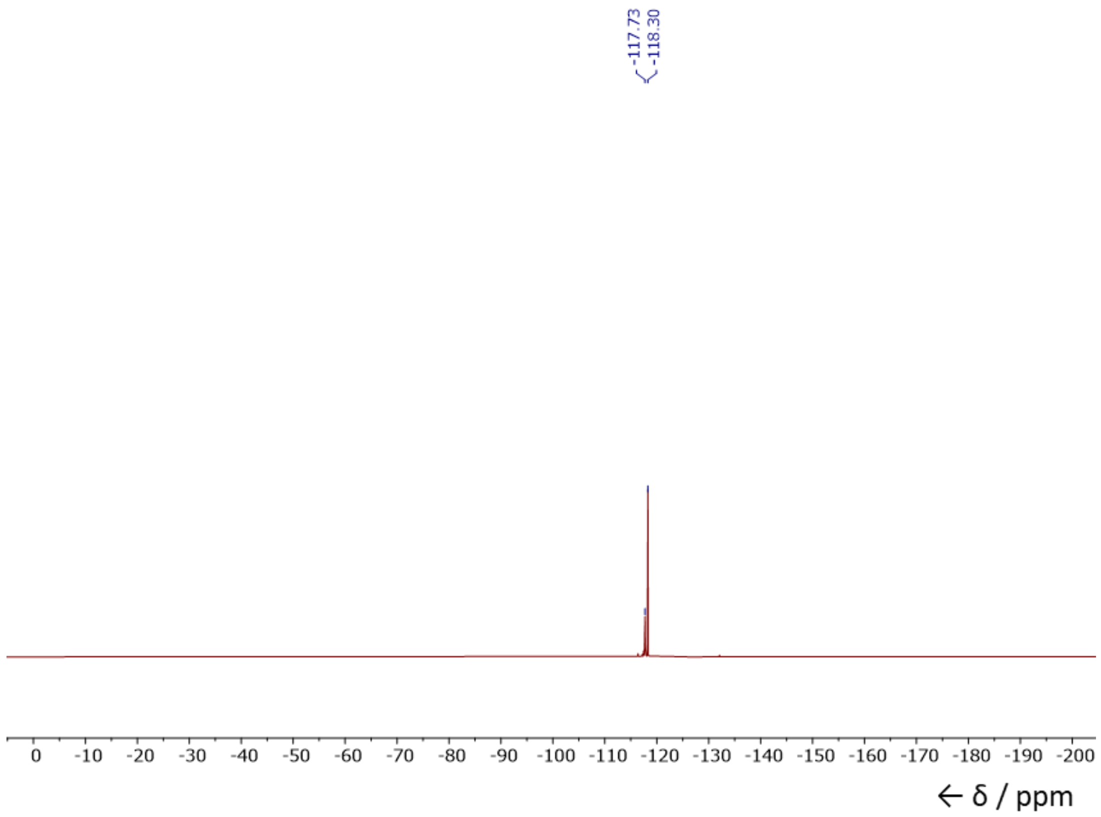

Figure S32. *^{19}F NMR spectrum of compound **24** ($CDCl_3$, 565 MHz, 298 K).*

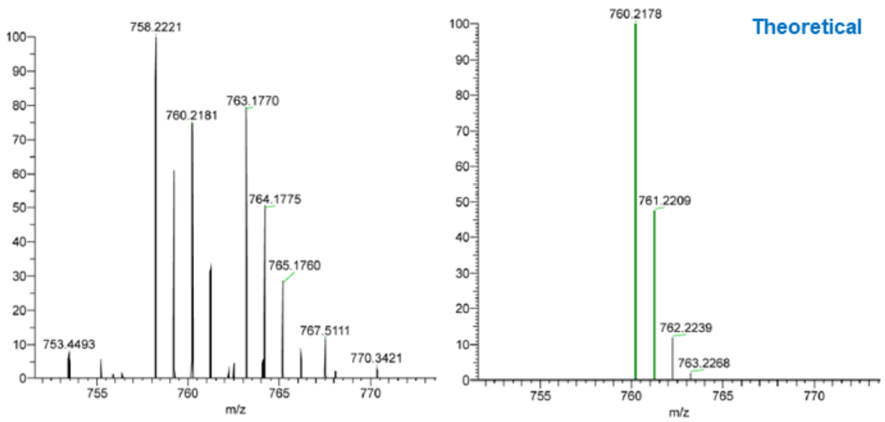

Figure S33. *HRMS spectrum of compound **24**. HRMS-ESI (m/z) calculated for $C_{40}H_{31}O_5N_5F_4Na$ $[M+Na]^+$, 760.2178; found 760.2181.*

2.11 (R)-1-palmitoyl-2-(*N*-fmoc-dodecyl)-*sn*-glycero-3-phosphocholine 25.

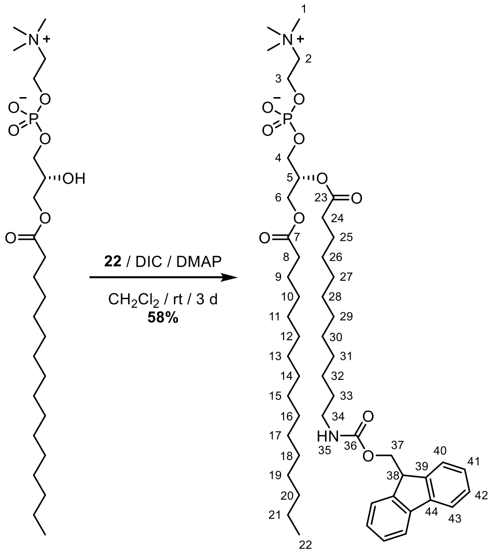

This known compound[11] was prepared *via* a novel procedure: 12-(Fmoc-amino)dodecanoic acid (2.2 g, 4.9 mmol, 5 Equiv.), DIC (1.2 mL, 7.9 mmol, 8 Equiv.) and DMAP (180 mg, 1.5 mmol, 1.5 Equiv.) were added to a solution of 16:0 lyso PC **11** (490 mg, 1.0 mmol, 1 Equiv.) in anhydrous CH_2Cl_2 (72 mL) under N_2. After stirring the reaction mixture for 3 days, DOWEX 50 WX2-400 was added to remove DMAP. The solution was allowed to stir for 45 minutes. The reaction mixture was filtered, washed with $CHCl_3$:MeOH (1:1, 100 mL) and concentrated. The crude material was purified by silica gel flash chromatography (0 - 2% H_2O / 25% MeOH / $CHCl_3$) to afford the title compound as a white solid (524 mg, 0.57 mmol, 58%). The characterisation matches that reported in the literature.[11]

^{1}H NMR (400 MHz, $CDCl_3$:CD_3OD (2:1)) δ 7.74 (d, *J* = 7.5 Hz, 2H, H_{43}), 7.59 (d, *J* = 7.4 Hz, 2H, H_{40}), 7.37 (t, *J* = 7.4 Hz, 2H, H_{42}), 7.29 (td, *J* = 7.4, 1.0 Hz, 2H, H_{41}), 5.84 (t, *J* = 5.4 Hz, 1H, H_{35}), 5.23 – 5.18 (m, 1H, H_5), 4.42 – 4.33 (m, 3H, $H_{6\,\&\,37}$), 4.28 – 4.15 (m, 3H, $H_{3\,\&\,38}$), 4.14 (dd, *J* = 12.1, 7.2 Hz, 1H, H_6), 3.96 (t, *J* = 6.0 Hz, 2H, H_4), 3.64 – 3.54 (m, 2H, H_2), 3.18 (s, 9H, H_1), 3.10 (t, *J* = 6.5 Hz, 2H, H_{34}), 2.33 – 2.26 (m, 4H, $H_{8\,\&\,24}$), 1.65 – 1.51 (m, 4H, $H_{9\,\&\,25}$), 1.50 – 1.42 (m, 2H, H_{33}), 1.34 – 1.18 (m, 38H, $H_{10\text{-}21\,\&\,26\text{-}32}$), 0.85 (t, *J* = 6.8 Hz, 3H, H_{22}).

LRMS-ESI (m/z) Calculated for $C_{51}H_{84}O_{10}N_2P$ $[M+H]^+$, 915.59; found 915.483.

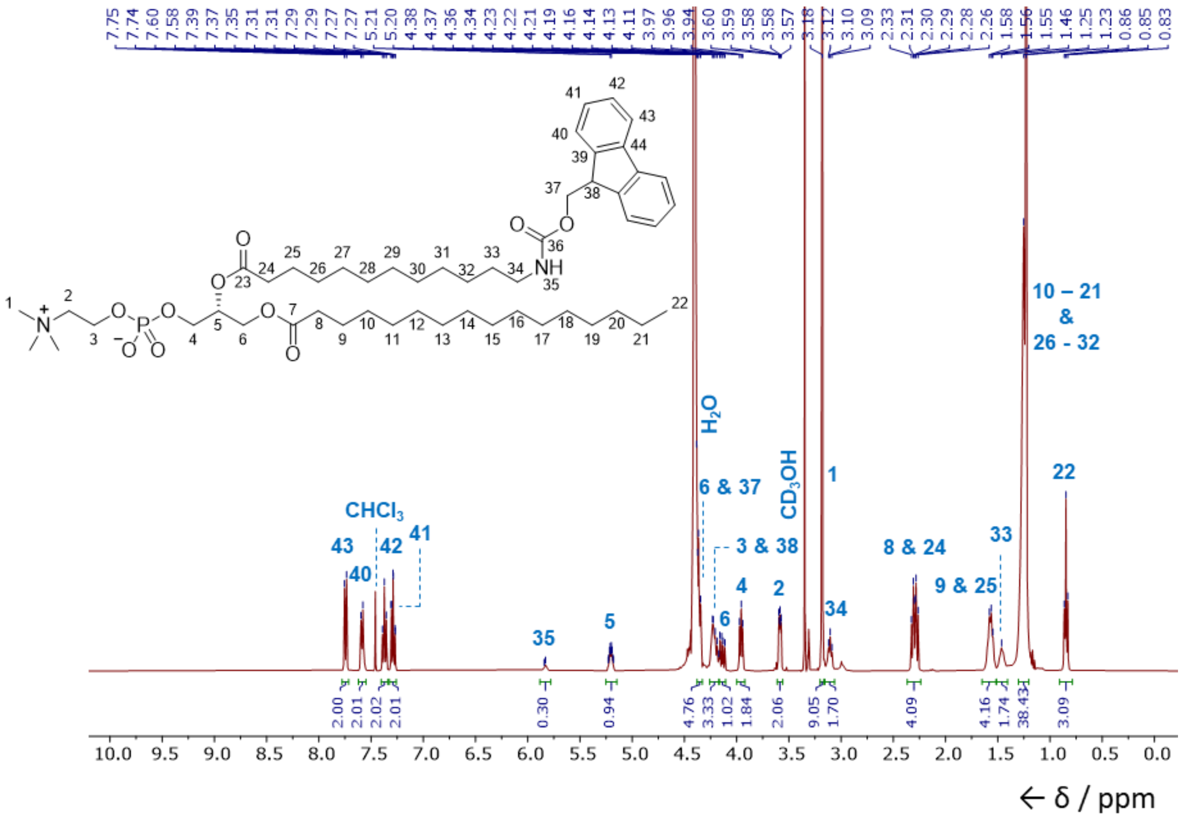

Figure S34. *^{1}H NMR spectrum of compound **25** (CDCl$_3$:CD$_3$OD (2:1), 400 MHz, 298 K).*

2.12 Mtt-protected long azobenzene lipid 26.

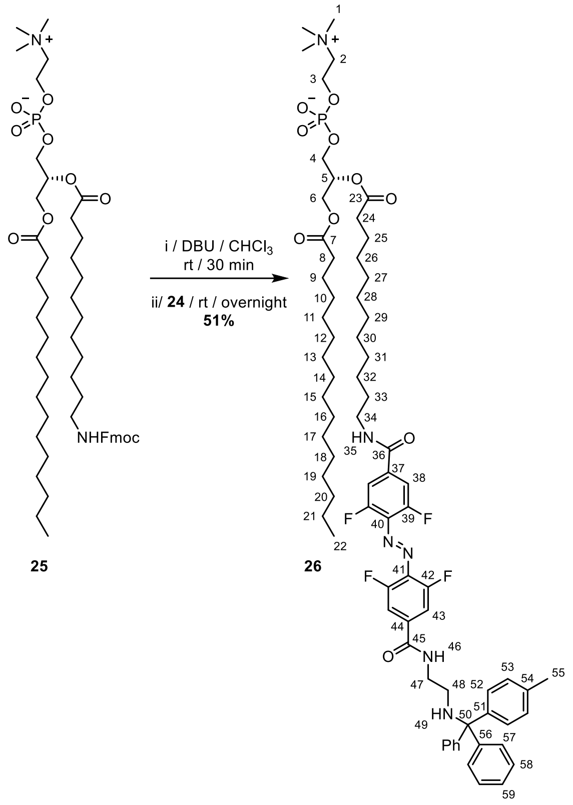

DBU (40 µL, 0.27 mmol, 2 Equiv.) was added to a stirred solution of **25** (120 mg, 0.13 mmol, 1 Equiv.) in anhydrous $CHCl_3$ (2 mL) and left to stir for 1 h under N_2. Compound **24** (116 mg, 0.16 mmol, 1.2 Equiv.) in anhydrous $CHCl_3$ (1 mL) was added to the reaction mixture and left to stir overnight under N_2. The reaction mixture was concentrated and purified by silica gel flash chromatography (0 - 2% H_2O / 25% MeOH / $CHCl_3$) to afford the title compound as a red solid (88.1 mg, 0.07 mmol, 51%).

^{1}H NMR (400 MHz, $CDCl_3$:CD_3OD (2:1)) δ 7.61 – 7.56 (m, 4H, $H_{38\,\&\,43}$), 7.42 (d, *J* = 7.2 Hz, 4H, H_{57}), 7.29 (d, *J* = 8.3 Hz, 2H, H_{52}), 7.25 – 7.20 (m, 4H, H_{58}), 7.15 (d, *J* = 7.3 Hz, 2H, H_{59}), 7.04 (d, *J* = 8.1 Hz, 2H, H_{53}), 5.22 – 5.17 (m, 1H, H_5), 4.38 (dd, *J* = 12.0, 3.1 Hz, 1H, H_6), 4.25 – 4.19 (m, 2H, H_3), 4.12 (dd, *J* = 12.0, 6.9 Hz, 1H, H_6), 3.96 (t, *J* = 6.1 Hz, 2H, H_4), 3.64 – 3.55 (m, 2H, H_2), 3.50 (t, *J* = 6.0 Hz, 2H, H_{47}), 3.36 (t, *J* = 7.3 Hz, 2H, H_{34}), 3.19 (s, 9H, H_1), 2.68 (s, 1H, H_{49}), 2.42 (t, *J* = 6.0 Hz, 2H, H_{48}), 2.32 – 2.26 (m, 4H, $H_{8\,\&\,24}$), 2.26 (s, 3H, H_{55}), 1.63 – 1.53 (m, 6H, $H_{9,\,25\,\&\,33}$), 1.35 – 1.19 (m, 38H, $H_{10-21\,\&\,26-32}$), 0.84 (t, *J* = 6.8 Hz, 3H, H_{22}).

^{13}C NMR (151 MHz, $CDCl_3$:CD_3OD (2:1)) δ 174.8 (C_7), 174.4 (C_{23}), 165.9 (C_{36}), 165.9 (C_{45}), 157.0 (dd, *J* = 7.1, 3.9 Hz), 155.3 (dd, *J* = 7.0, 3.9 Hz), 145.3, 141.7, 139.4 (t, *J* = 8.4 Hz), 138.6, 137.5, 134.1 (t, *J* = 10.1 Hz), 133.9 (t, *J* = 10.3 Hz), 129.6, 129.5, 128.9, 128.5, 127.7, 113.0 – 112.7 (m) ($C_{38\text{ or }43}$), 112.8 – 112.5 (m) ($C_{38\text{ or }43}$), 73.2 (C_{50}), 71.4 (d, *J* = 8.0 Hz) (C_5), 67.2 (C_2), 64.6 (d, *J* = 4.9 Hz) (C_4), 63.5 (C_6), 60.1 (d, *J* = 4.9 Hz) (C_3), 54.6 (C_1), 45.1 (C_{48}), 41.2 (C_{34}), 37.6 (C_{47}), 34.9(C_8), 34.8 (C_{24}), 32.8, 30.5, 30.5, 30.5, 30.5, 30.5, 30.4, 30.4, 30.4, 30.4, 30.3, 30.3, 30.3, 30.2, 30.2, 30.0, 29.9, 29.9, 27.8, 25.8, 25.7, 23.5, 21.0 (C_{55}), 14.4 (C_{22}).

^{19}F NMR (565 MHz, $CDCl_3$:CD_3OD (2:1)) δ -120.64 ($F_{39\text{ or }42}$), -120.70 ($F_{39\text{ or }42}$).

HRMS-ESI (m/z) Calculated for $C_{72}H_{99}O_{10}N_6F_4P$ $[M+H]^+$, 1315.7169; found 1315.7120.

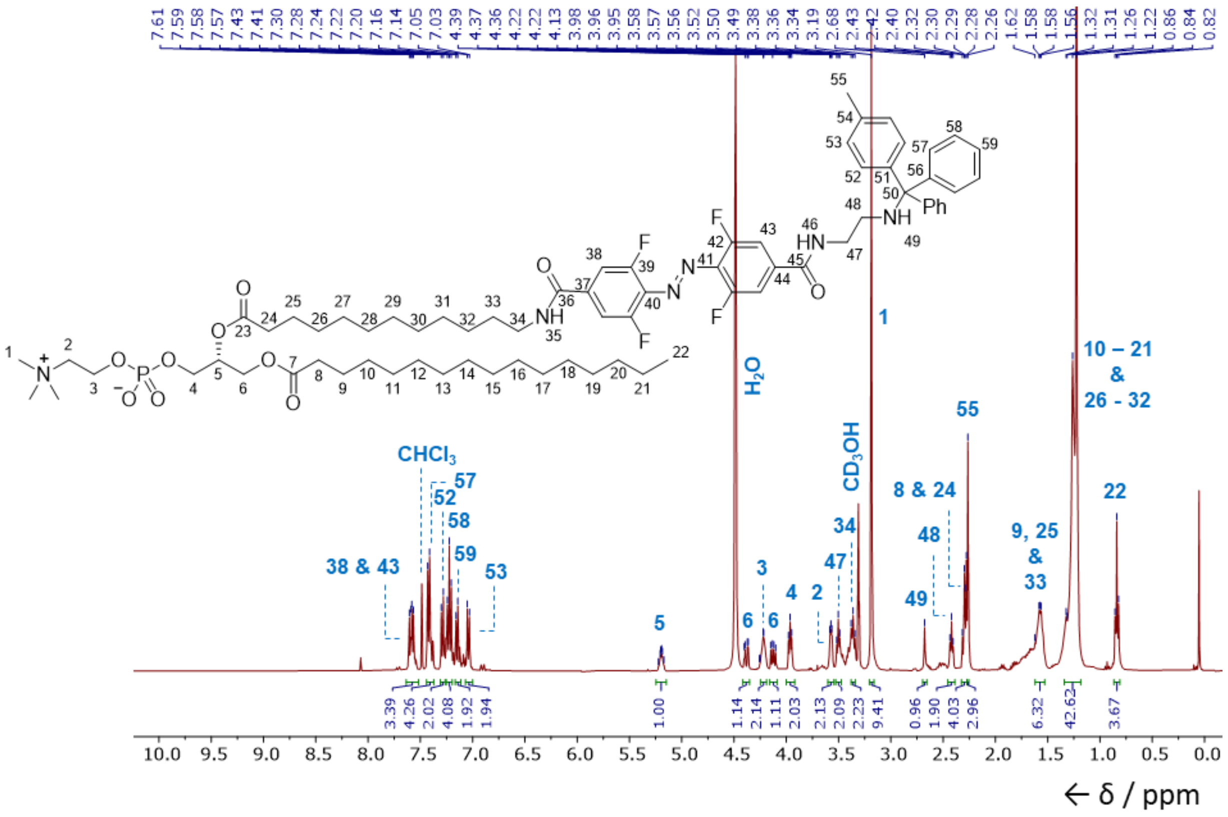

Figure S35. ^{1}H NMR spectrum of compound **26** ($CDCl_3$:CD_3OD (2:1), 400 MHz, 298 K).

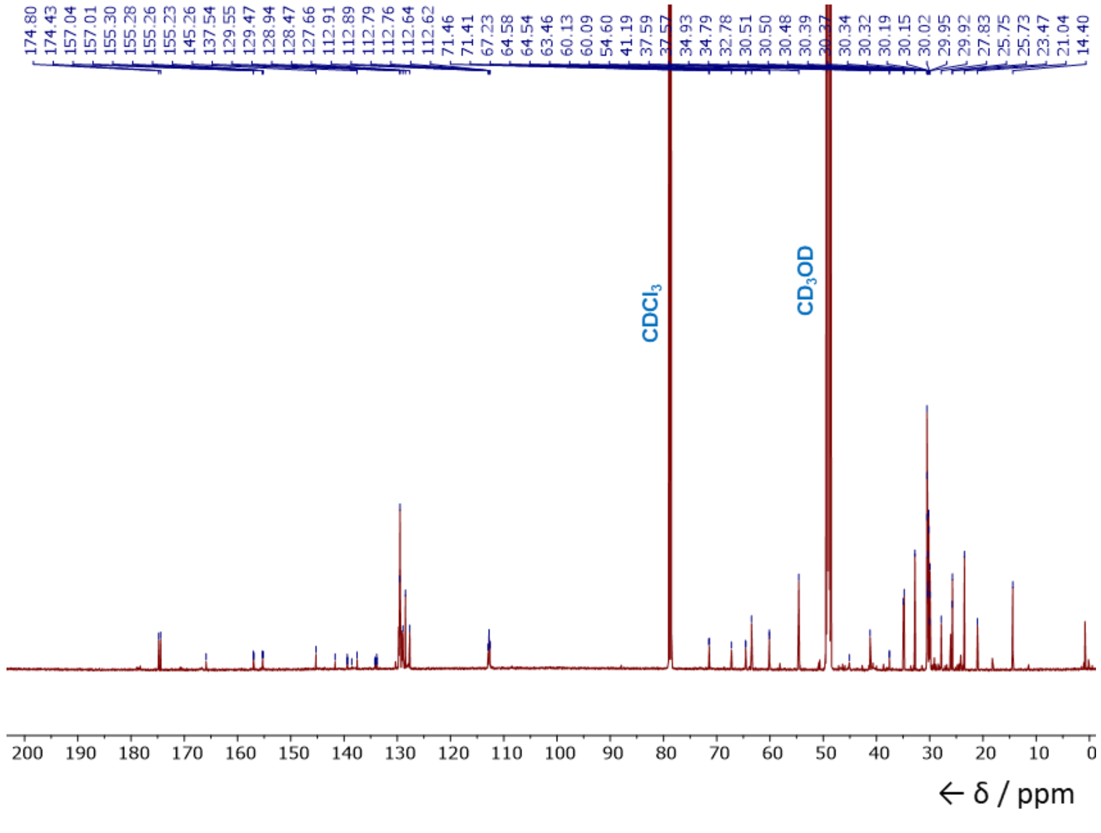

Figure S36. ^{13}C NMR spectrum of compound **26** ($CDCl_3$:CD_3OD (2:1), 151 MHz, 298 K).

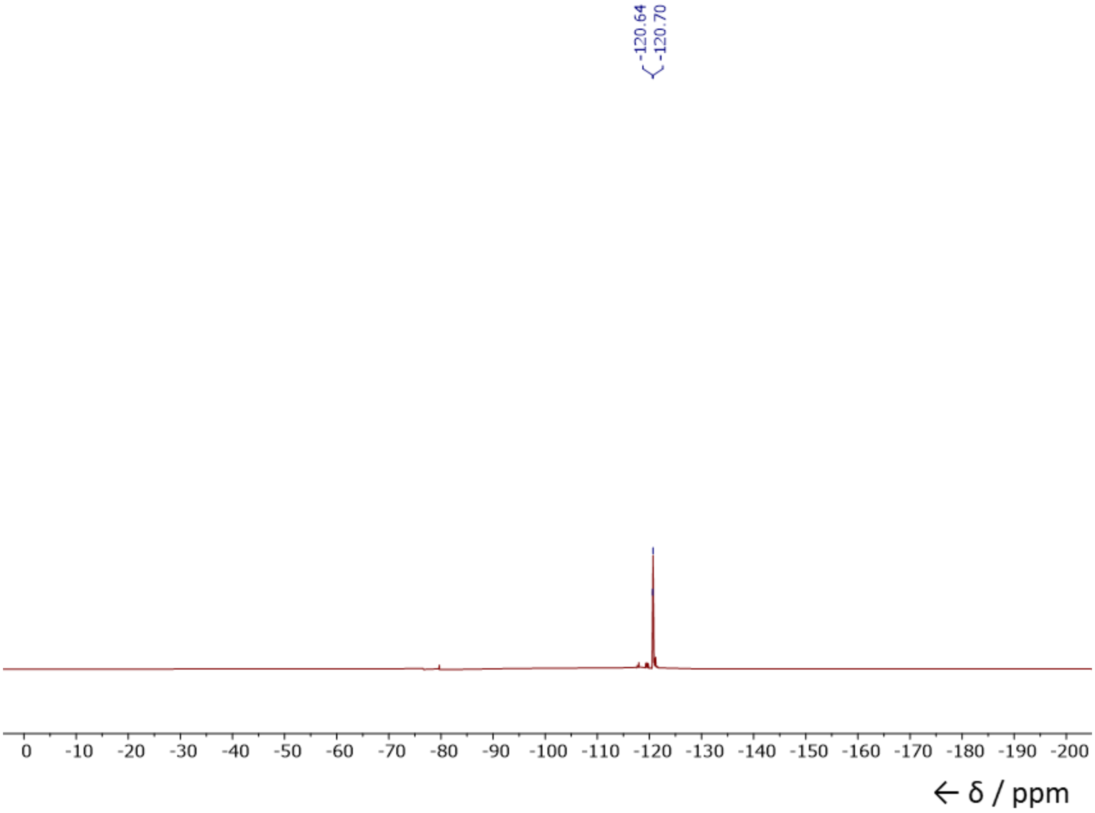

Figure S37. *^{19}F NMR spectrum of compound* ***26*** *($CDCl_3$:CD_3OD (2:1), 565 MHz, 298 K).*

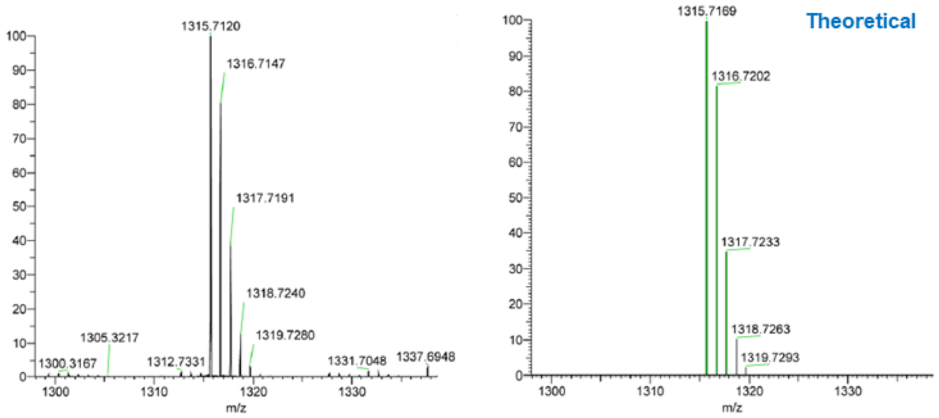

Figure S38. *HRMS spectrum of compound* ***26****. HRMS-ESI (m/z) calculated for $C_{72}H_{99}O_{10}N_6F_4P$ $[M+H]^+$, 1315.7169; found 1315.7120.*

2.13 Long relay transporter 4.

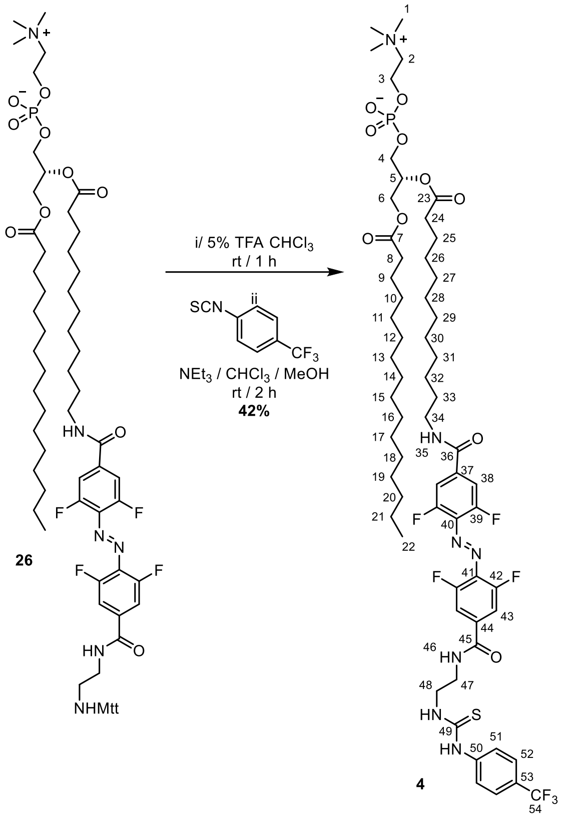

Mtt-protected long azobenzene lipid **26** (20 mg, 0.015 mmol, 1 Equiv.) was dissolved in anhydrous $CHCl_3$ (1 mL) and TFA (50 µL, 5% v/v) and left to stir at rt for 1 h under N_2. After complete deprotection, MeOH (1 mL) was added and the reaction mixture was concentrated. The residue was dissolved in anhydrous $CHCl_3$:MeOH (1:1, 2 mL) and NEt_3 (50 µL). 4-(Trifluoromethyl)phenyl isothiocyanate (6.2 mg, 0.03 mmol, 2 Equiv.) was added to the reaction mixture and left to stir at rt for 5 h under N_2. The reaction mixture was then concentrated and purified by silica gel flash chromatography (0 - 3% H_2O / 25% MeOH / $CHCl_3$) to afford the title compound as a red solid (8 mg, 0.006 mmol, 42%).

^{1}H NMR (400 MHz, $CDCl_3$:CD_3OD (2:1)) δ 7.65 – 7.58 (m, 4H, $H_{38\,\&\,43}$), 7.57 (s, 4H, $H_{51\,\&\,52}$), 5.26 – 5.14 (m, 1H, H_5), 4.42 – 4.35 (m, 1H, H_6), 4.25 – 4.20 (m, 2H, H_3), 4.12 (dd, *J* = 12.0, 6.8 Hz, 1H, H_6), 4.00 – 3.94 (t, *J* = 5.7 Hz, 2H, H_{48}), 3.91 – 3.85 (m, 2H, H_4), 3.62 (t, *J* = 5.7 Hz, 2H, H_{47}), 3.60 – 3.55 (m, 2H, H_2), 3.37 (t, *J* = 7.1 Hz, 2H, H_{34}), 3.18 (s, 9H, H_1), 2.31 – 2.25 (m, 4H, $H_{8\,\&\,24}$), 1.64 – 1.51 (m, 6H, $H_{9,\,25\,\&\,38}$), 1.36 – 1.18 (m, 38H, $H_{10-21\,\&\,26-32}$), 0.84 (t, *J* = 6.8 Hz, 3H, H_{22}).

^{13}C NMR (151 MHz, $CDCl_3$:CD_3OD (2:1)) δ 182.1, 174.4 (C_7), 173.9(C_{23}), 165.9 (C_{45}), 165.2 (C_{36}), 156.5 – 156.3 (m), 154.8 – 154.6 (m), 142.1, 138.7 (t, *J* = 8.5 Hz), 137.8 (t, *J* = 8.3 Hz), 133.6 (t, *J* = 10.8 Hz), 133.3 (t, *J* = 10.5 Hz), 127.1, 126.4 ($C_{51\text{ or }52}$), 125.4, 123.8 ($C_{51\text{ or }52}$), 112.5 – 112.2 (m), 112.3 – 112.0 (m), 70.6 (C_5), 66.7 (C_2), 64.3 (C_4), 62.9 (C_6), 59.6 (C_3), 54.4 (C_1), 44.1 (C_{48}), 40.7 (C_{47}), 40.7 (C_{34}), 34.5 ($C_{8\text{ or }24}$), 34.4 ($C_{8\text{ or }24}$), 32.2, 30.0, 30.0, 30.0, 29.9, 29.9, 29.9, 29.8, 29.8, 29.8, 29.7, 29.7, 29.6, 29.6, 29.5, 29.4, 29.4, 27.3, 25.2($C_{9\text{ or }25}$), 25.2 ($C_{9\text{ or }25}$), 23.0, 18.03, 14.2 (C_{22}).

^{19}F NMR (565 MHz, $CDCl_3$:CD_3OD (2:1)) δ -62.71 (F_{54}), -119.84 ($F_{39\text{ or }42}$), -119.92 ($F_{39\text{ or }42}$).

HRMS-ESI (m/z) Calculated for $C_{60}H_{87}O_{10}N_7F_7PS$ $[M+H]^+$, 1262.5934; found 1262.5902.

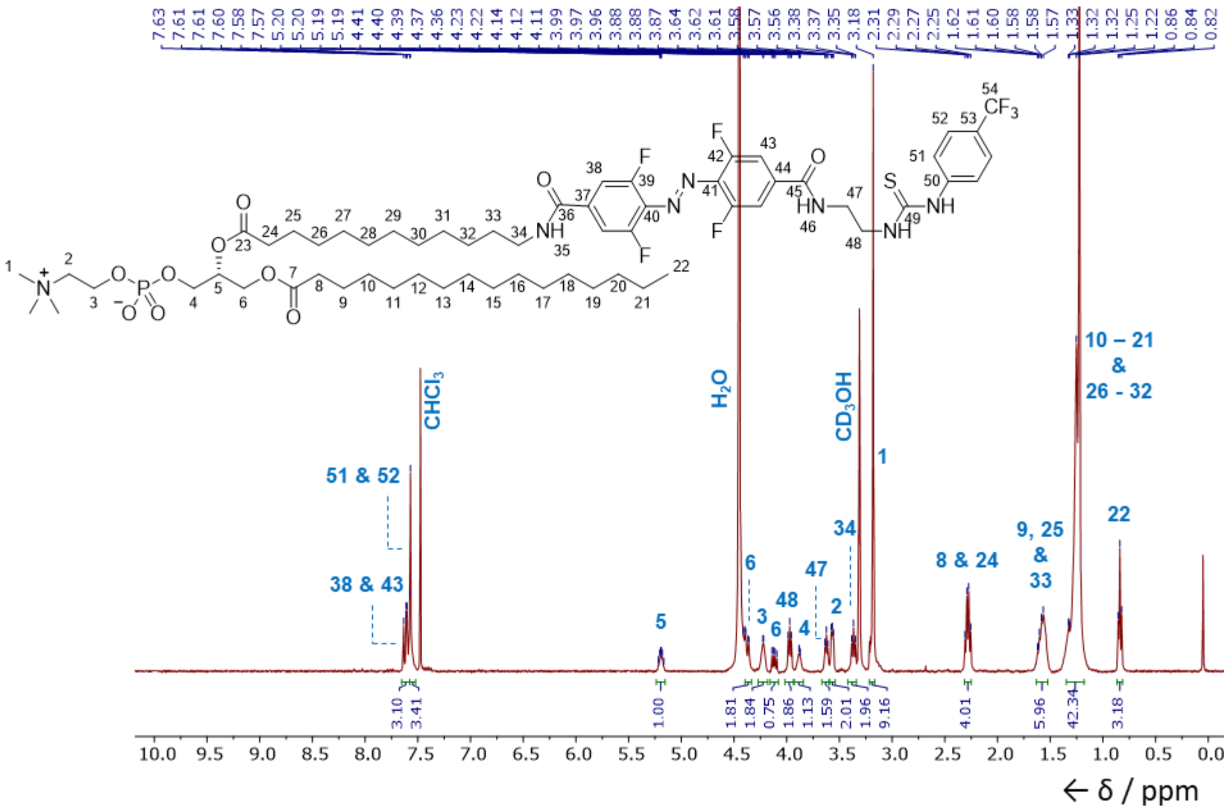

Figure S39. *^{1}H NMR spectrum of compound **4** (CDCl₃:CD₃OD (2:1), 400 MHz, 298 K).*

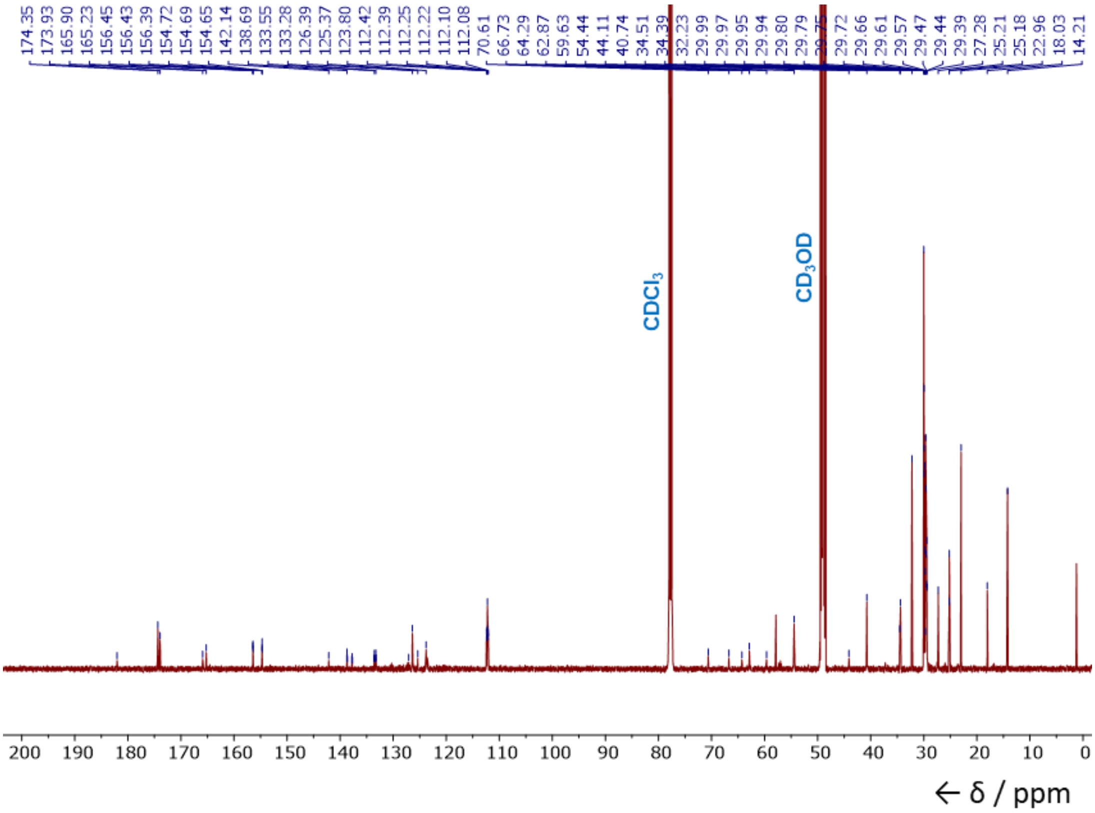

Figure S40. *^{13}C NMR spectrum of compound **4** (CDCl₃:CD₃OD (2:1)), 151 MHz, 298 K).*

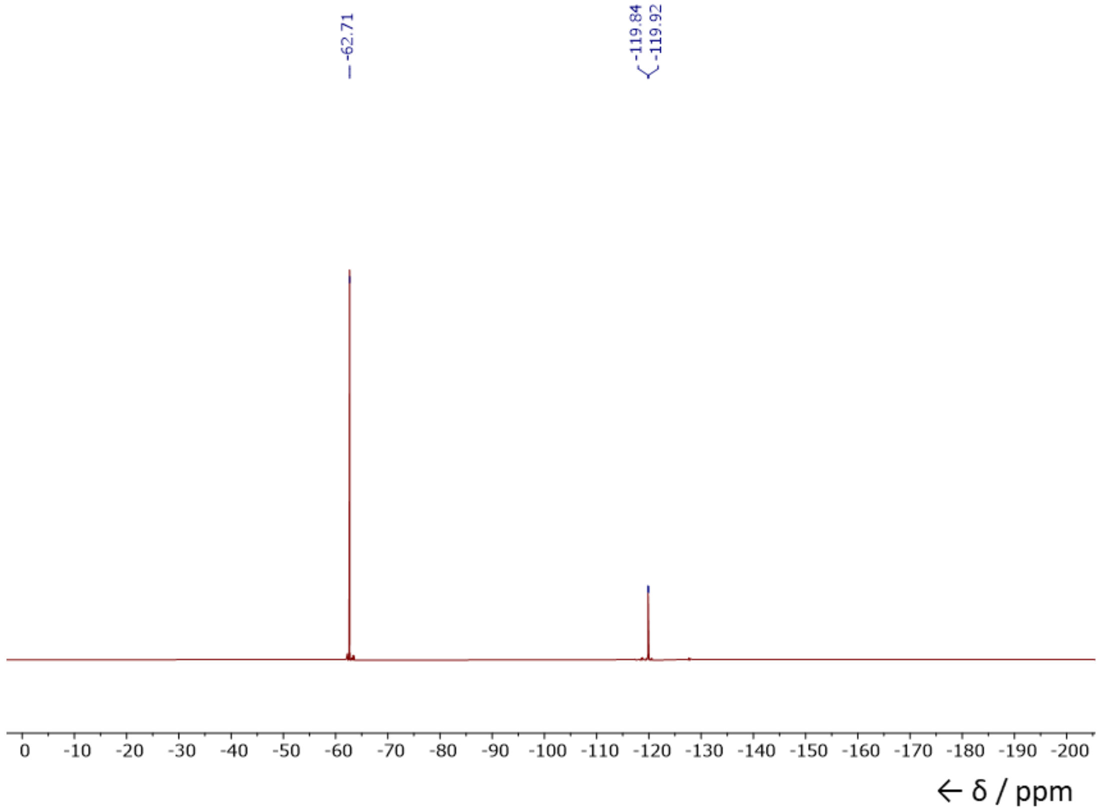

Figure S41. *^{19}F NMR spectrum of compound **4** ($CDCl_3$:CD_3OD (2:1), 565 MHz, 298 K).*

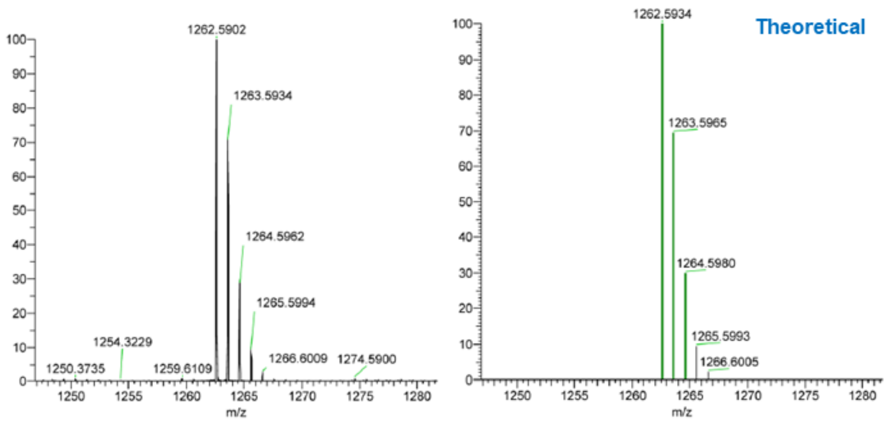

Figure S42. *HRMS spectrum of compound **4**. HRMS-ESI (m/z) calculated for $C_{60}H_{87}O_{10}N_7F_7PS$ $[M+H]^+$, 1262.5934; found 1262.5902.*

3 Photo-isomerisation Experiments

Photo-irradiation of liquid samples was carried out using Thorlabs high-power mounted LEDs (models M530L4 (530 nm, 370 mW) and M405L4 (405 nm, 1000 mW)) using in-house custom built set-up with optical components supplied by Thorlabs, as described previously.[12] For irradiating small vials of samples, NMR tubes and cuvettes, a Thorlabs cuvette holder (CVH100/M) equipped with the mounted LEDs was used. Samples were irradiated for sufficient time to reach the photo-stationary state, as confirmed by ^{1}H NMR or UV-vis experiments.

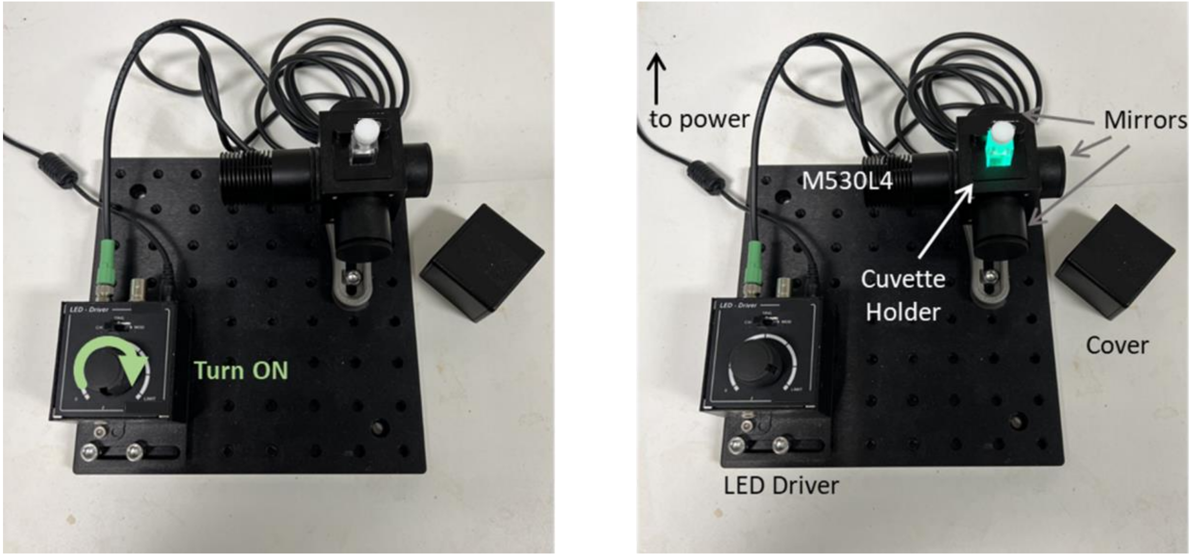

Figure S43. *Apparatus for photo-irradiating cuvettes and liquid samples in small vials.*

3.1 Determination of photo-stationary states (PSS) in solution

The photo-stationary state composition for both 405 nm and 530 nm irradiation was determined by ^{1}H NMR spectroscopy following irradiation of the sample in solution in the NMR tube with LEDs until there was no further change in isomer composition.

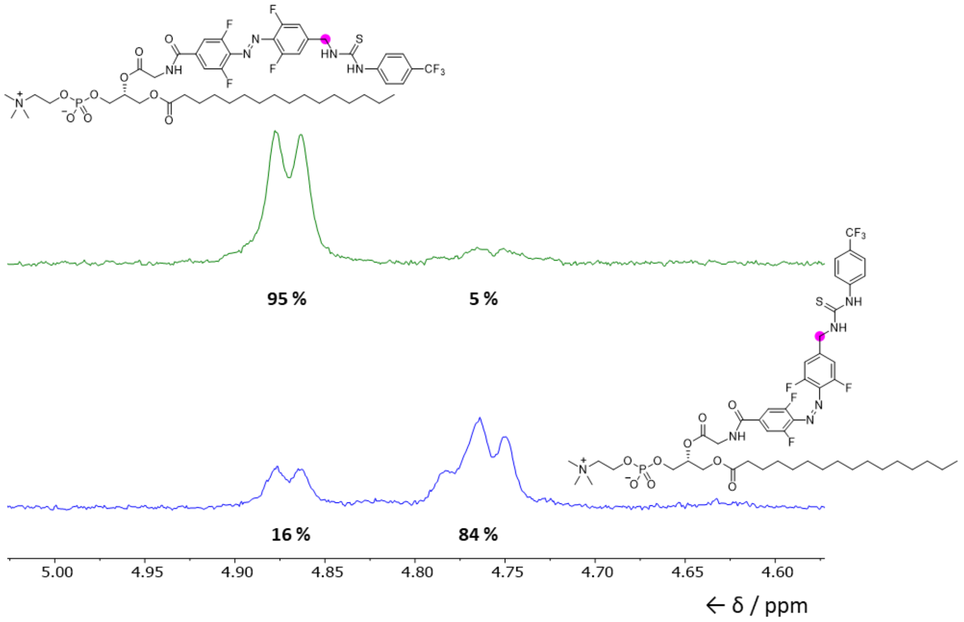

Figure S44. *Partial* [1]*H NMR spectra of* ***1***^{E}/***1***^{Z} *in DMSO-d*$_6$*, showing the PSS achieved by irradiation with blue 405 nm (95% E) and green 530 nm (84% Z) light.*

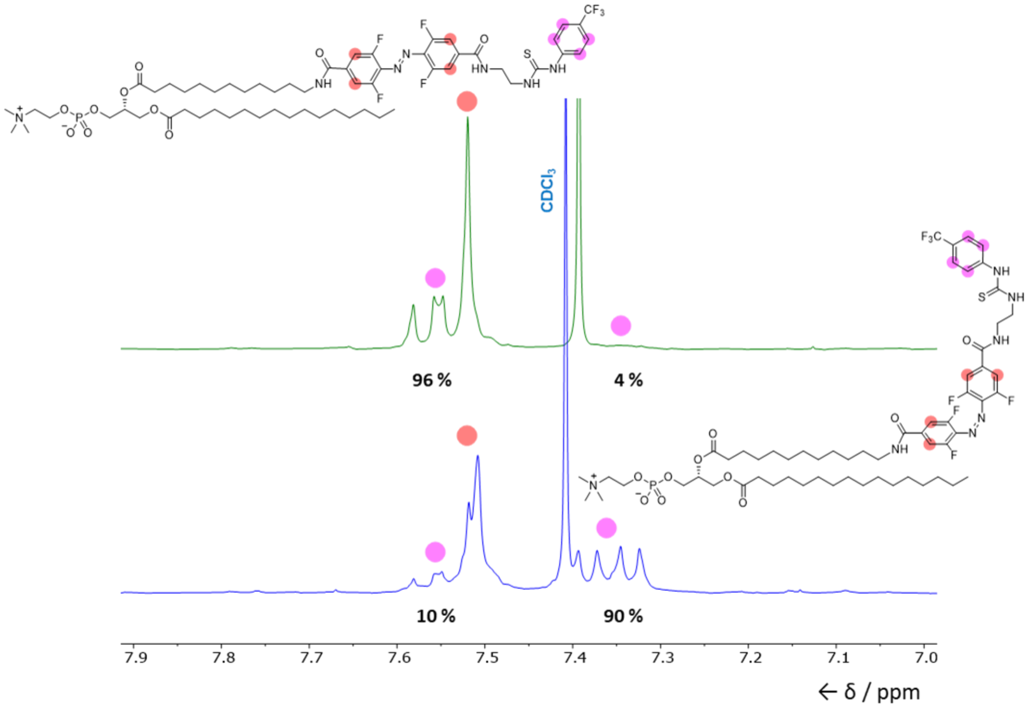

Figure S45. *Partial* [1]*H NMR spectra of* ***4***^{E}/***4***^{Z} *in* $CDCl_3$:CD_3OD *(2:1), showing the PSS achieved by irradiation with blue 405 nm (96% E) and green 530 nm (90% Z) light.*

4 UV-Visible Absorption Analysis

4.1 Solution Phase Analysis

Samples were prepared in DMSO, and photo-switched using both 405 nm and 530 nm irradiation until the photo-stationary state was reached and there was no further change in the spectra.

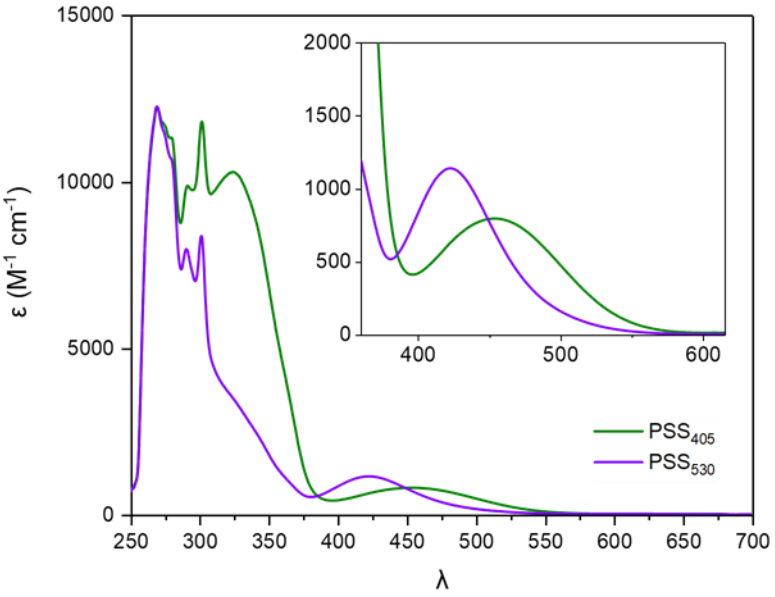

Figure S46. *UV-vis spectra of PSS distributions of* ***9*** *in DMSO, measured after irradiation with 405 nm (95% E) or 530 nm (84% Z) light.*

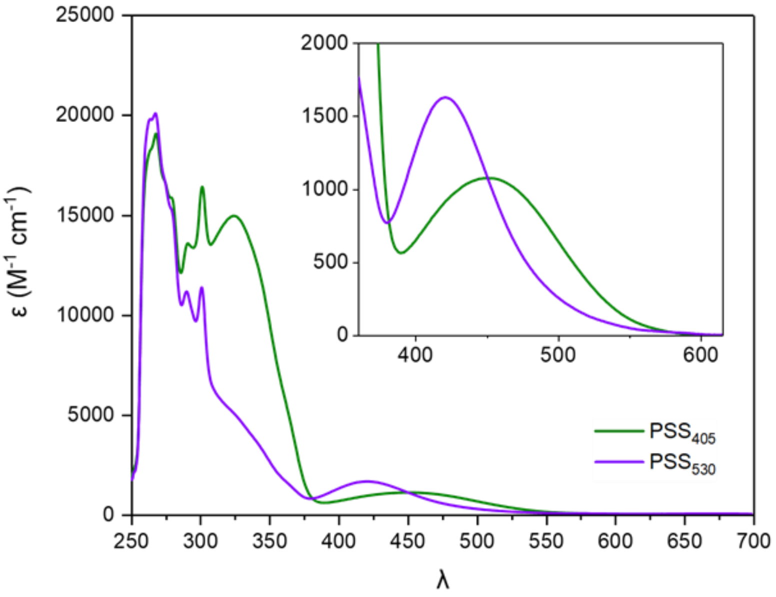

Figure S47. *UV-vis spectra of PSS distributions of **2** in DMSO, measured after irradiation with 405 nm (95% E) or 530 nm (84% Z) light.*

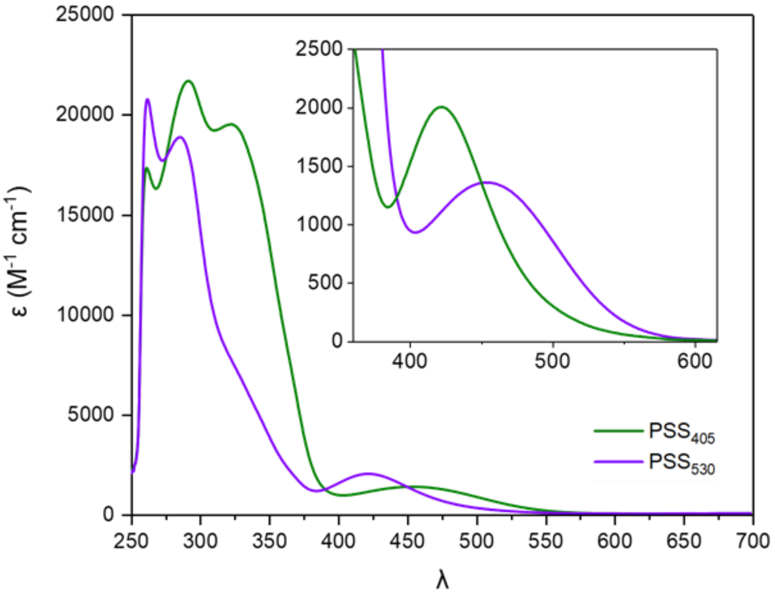

Figure S48. *UV-vis spectra of PSS distributions of **1** in DMSO, measured after irradiation with 405 nm (95% E) or 530 nm (84% Z) light.*

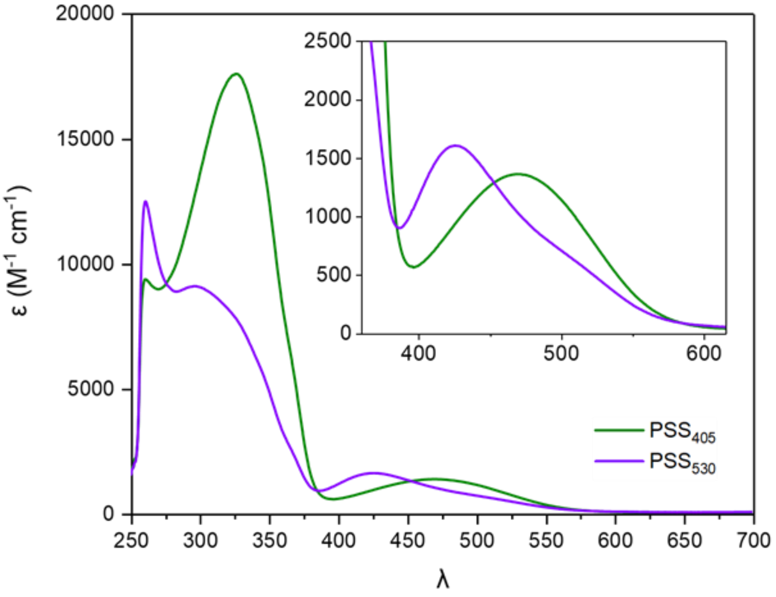

Figure S49. *UV-vis spectra of PSS distributions of **20** in DMSO, measured after irradiation with 405 nm (96% E) or 530 nm (90% Z) light.*

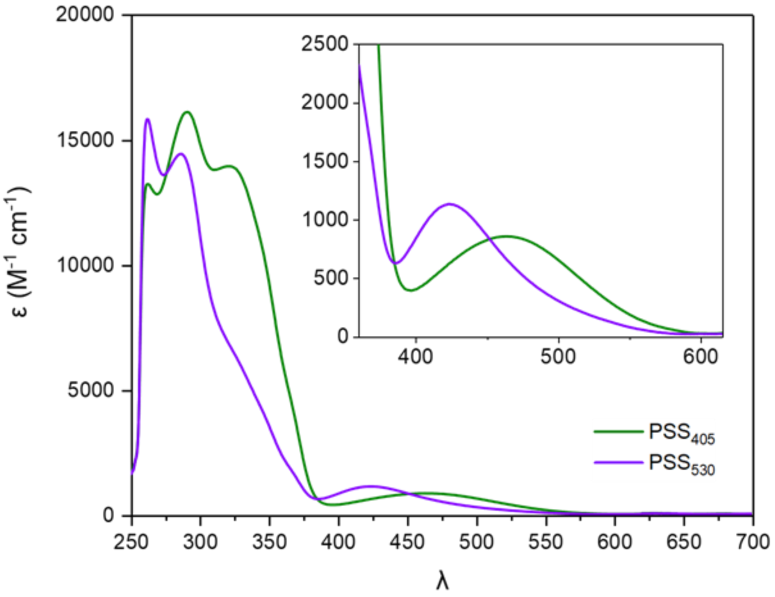

Figure S50. *UV-vis spectra of PSS distributions of **3** in DMSO, measured after irradiation with 405 nm (96% E) or 530 nm (90% Z) light.*

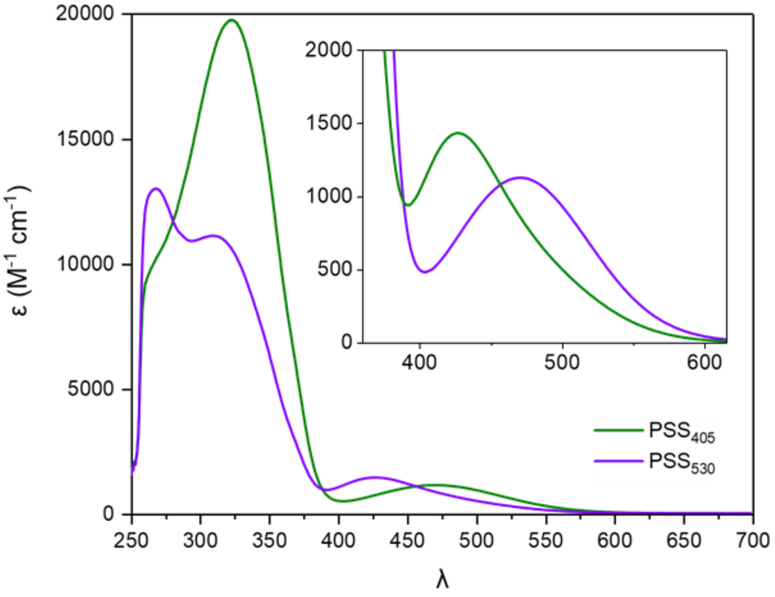

Figure S51. *UV-vis spectra of PSS distributions of **24** in DMSO, measured after irradiation with 405 nm (96% E) or 530 nm (90% Z) light.*

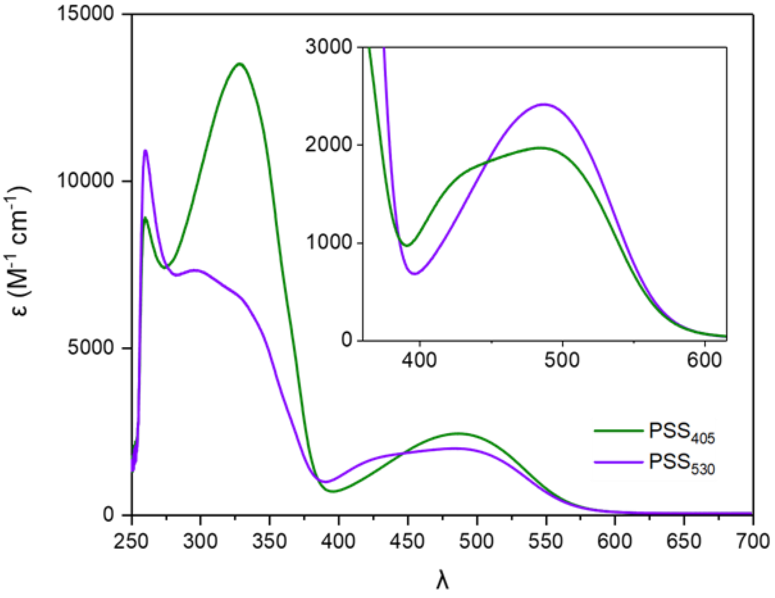

Figure S52. *UV-vis spectra of PSS distributions of **26** in DMSO, measured after irradiation with 405 nm (96% E) or 530 nm (90% Z) light.*

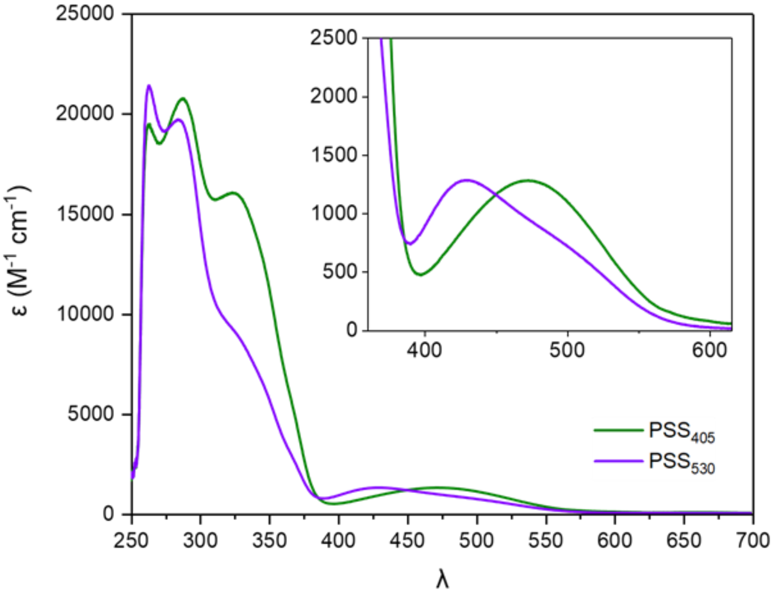

Figure S53. *UV-vis spectra of PSS distributions of **4** in DMSO, measured after irradiation with 405 nm (96% E) or 530 nm (90% Z) light.*

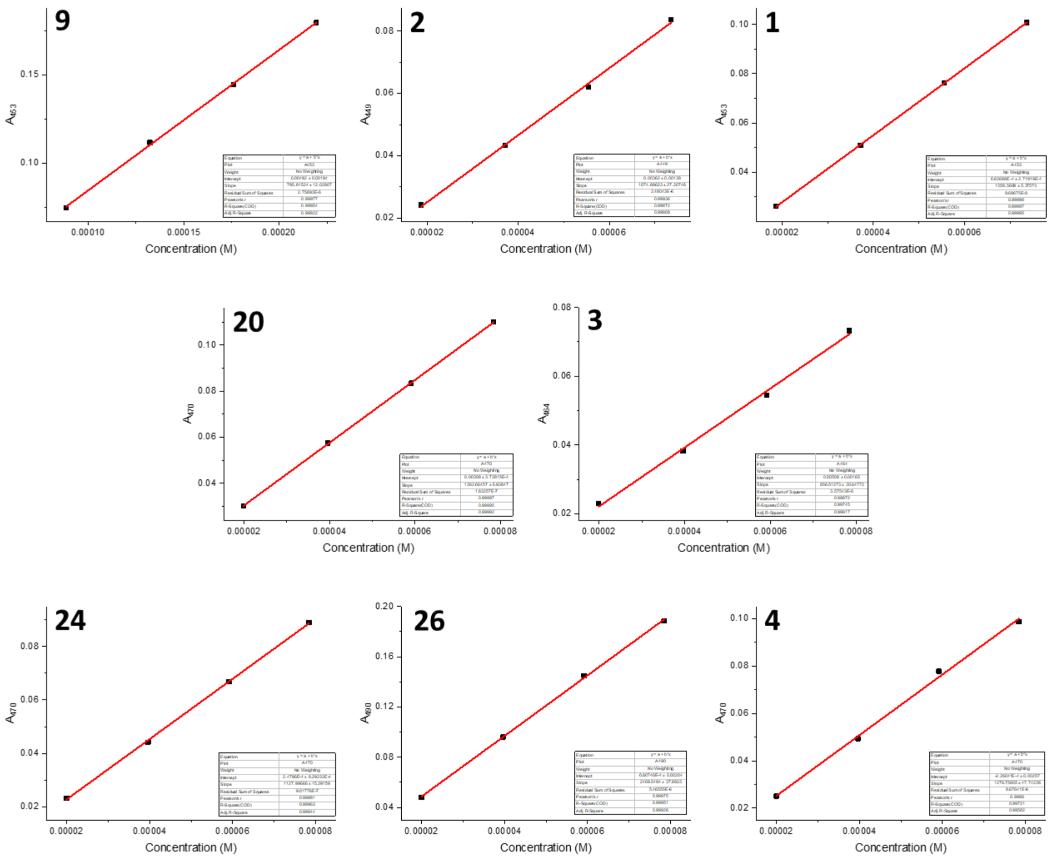

Figure S54. *Beer-Lambert plots for azobenzene derivatives **1-4, 9, 20, 24, 26**.*

4.2 Lipid Bilayer Analysis

UV-vis analysis was employed to determine the incorporation efficiency of the transporter into the membrane of preformed LUVs and the *in-situ* photo-switching efficiency within the bilayer.

To 2.5 mL of POPC LUVs (200 nm, 0.50 mM lipid; internal and external buffer comprising 100 mM NaCl, 10 mM HEPES, pH 7.0) was added compound **1** or **4** in DMSO (10 mM, 6.25 µL, 5 mol% to lipid) and stirred for 10 min. A 0.5 mL aliquot of this solution was diluted to a final volume of 1.0 mL with buffer solution (0.5 mL, 100 mM NaCl, 10 mM HEPES, pH 7.0) to give the 'pre-size exclusion sample.' Another 1.75 mL aliquot of the initial solution was eluted through a size exclusion Sephadex G-25 column with buffer solution (100 mM NaCl, 10 mM HEPES, pH 7.0) collecting the vesicles in 3.5 mL of solvent to give the 'post-size exclusion sample'; with the same total concentration of vesicles. The UV-vis spectra were recorded and the baseline spectrum of POPC LUVs (200 nm, 0.25 mM lipid, 100 mM NaCl, 10 mM HEPES, pH 7.0) subtracted. The same procedure was repeated used POPC LUVs prepared with 5 mol% of **1** pre-incorporated in the lipid film prior to vesicle preparation to obtain data for the mixed pre-incorporated and externally added experiment.

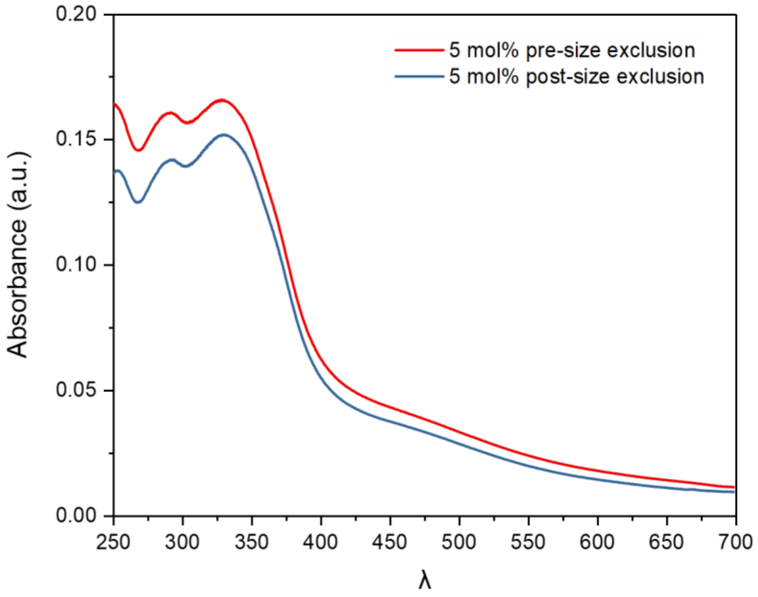

Figure S55. *UV-vis spectra of* $\mathbf{1}^{E}$ *(5 mol% to lipid) embedded in POPC LUVs pre- and post-size exclusion. Incorporation efficiency was determined to be ~90%.*

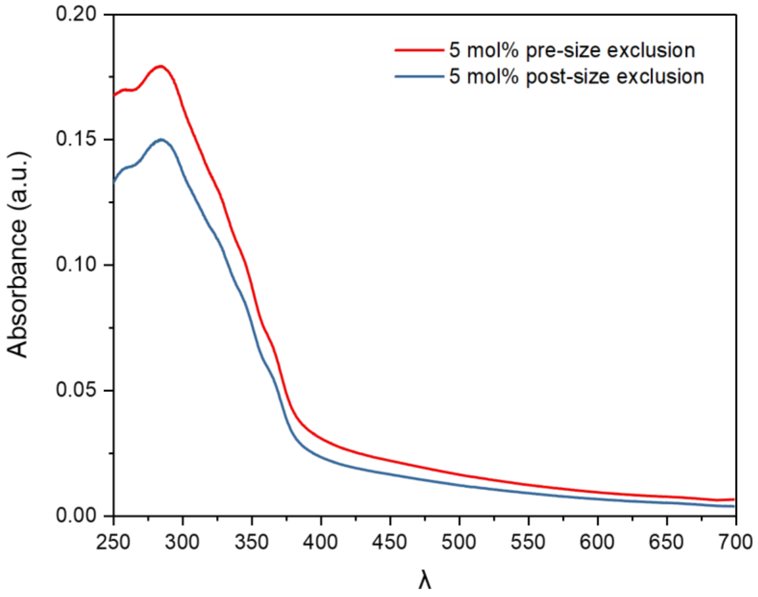

Figure S56. *UV-vis spectra of* ***4**^{E}* *(5 mol% to lipid) embedded in POPC LUVs pre- and post-size exclusion. Incorporation efficiency was determined to be ~90%.*

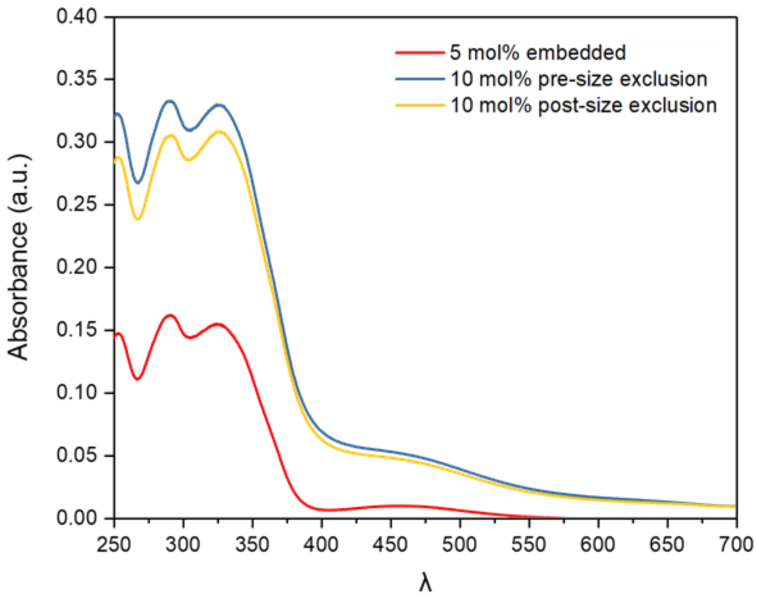

Figure S57. *UV-vis spectra of* ***1*** *(5 mol% to lipid) pre-incorporated into POPC LUVs is shown in red compared to the UV-vis spectra of* ***1*** *(10 mol% to lipid: 5 mol% pre-incorporated plus 5 mol% externally added as a DMSO solution) pre- and post-size exclusion. Incorporation efficiency was determined to be ~90%.*

To observe the *in-situ* photo-switching of compound **1** in the bilayer of POPC LUVs a similar procedure as above was employed to generate POPC LUVs with compound **1** embedded, but with a higher loading (34 mol% to lipid). In brief, **1** (50 µL of 8.4 mM DMSO stock) was added to 2 mL of POPC LUVs (200 nm, 0.625 mM lipid; internal and external buffer comprising 100 mM NaCl, 10 mM HEPES, pH 7.0). The post-size exclusion sample was then irradiated for 3 minutes with blue light (405 nm) and green light (530 nm) independently, which was sufficient to obtain the photo-stationary state distribution of the *E*- and *Z*-isomers in bilayer by UV-vis analysis. Compound **1** could be repeatedly photo-isomerised within the bilayer with no degradation after multiple cycles of irradiation.

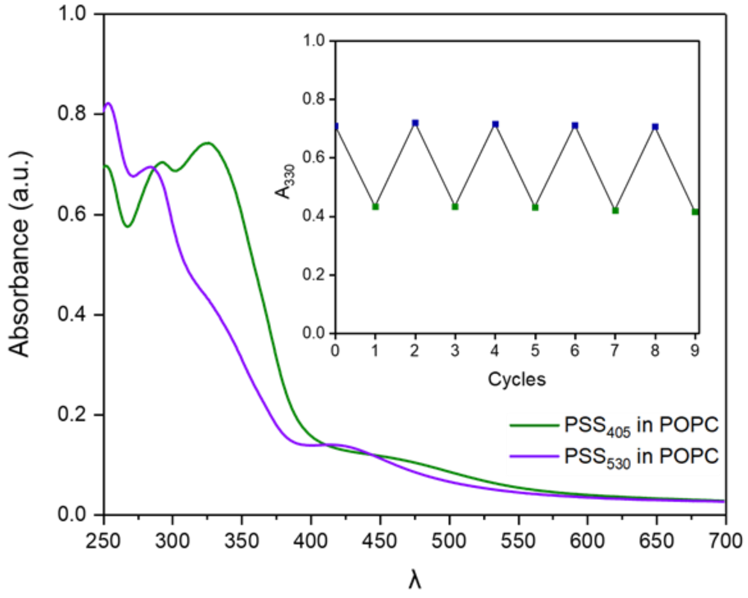

Figure S58. *UV-vis spectrum of **1** (31 mol% to lipid) in POPC LUVs measured after irradiation with 405 nm or 530 nm light for 3 min. Insert shows the reversibility of photo-switching within the bilayer, monitoring the absorbance at 330 nm.*

To determine the photo-stationary state of **1** in the bilayer, vesicles containing **1** were pre-irradiated with 405 nm or 530 nm and then dissolved in DMSO (25% aqueous LUV suspension, 75% DMSO) and the UV-vis spectrum recorded. Separately, **1** was pre-irradiated in DMSO solution and the PSS was confirmed by ^{1}H NMR analysis. This solution was diluted in an equivalent solution of 25% aqueous buffer (100 mM NaCl, 10 mM HEPES, pH 7.0) / 75% DMSO. The in-membrane photo-isomerised spectra and in-DMSO photo-isomerised spectra were identical, demonstrating that the photo-stationary state obtained in the bilayer is equivalent to that observed in DMSO solution (PSS_{530} = 84% $\mathbf{1}^{Z}$; PSS_{405} = 95% $\mathbf{1}^{E}$)

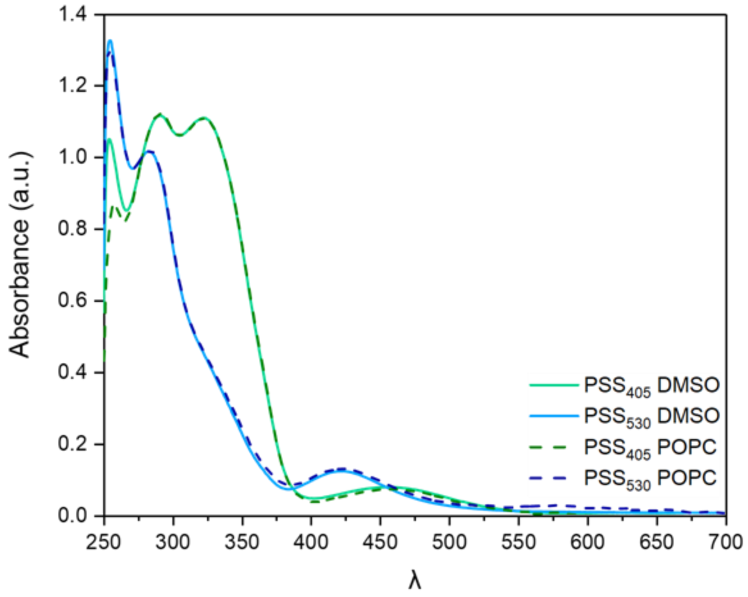

Figure S59. *UV-vis spectrum of **1** in 25% aqueous buffer (100 mM NaCl, 10 mM HEPES, pH 7.0) 75% DMSO. Compound **1** was pre-irradiated in the bilayer of POPC LUVs or DMSO solution with 405 nm or 530 nm light for 3 min prior to being diluted with DMSO or aqueous buffer and DMSO respectively. Data is normalised to 1 at the isosbestic point at 278 nm.*

Quantification of the thermal half-life of compound **1** in the bilayer was carried out following the change in absorption intensity at 330 nm over time, at numerous temperatures. Data for 25 °C was obtained for 31 mol% to lipid of $\mathbf{1}^{Z}$ in POPC LUVs (200 nm, 0.179 mM lipid; internal and external buffer comprising 100 mM NaCl, 10 mM HEPES, pH 7.0). Data for 45 °C and 60 °C was obtained for 5 mol% to lipid of $\mathbf{1}^{Z}$ in POPC LUVs (200 nm, 0.25 mM lipid; internal and external buffer comprising 100 mM NaCl, 10 mM HEPES, pH 7.0). The vesicles were irradiated with 530 nm light for 3 minutes immediately prior to starting the experiment. No appreciable thermal relaxation at 25 °C of the *ortho*-tetrafluorozobenzene core when embedded in the bilayer membrane was observed over more than 15 hrs, in agreement with previously reported related photo-switches.[13] The thermally promoted conversion of $\mathbf{1}^{Z} \rightarrow \mathbf{1}^{E}$ occurs too slowly at the elevated temperature of 45 °C to be fit to an exponential decay curve. At 60 °C (beyond which the integrity of the membrane will likely be compromised), the data allowed for determination of a thermal half-life of 24 hrs.

This data confirms that, due to the long half-life of the photoswitch in the bilayer environment, thermal relaxation is negligible under the conditions and timescales of all assays in this work.

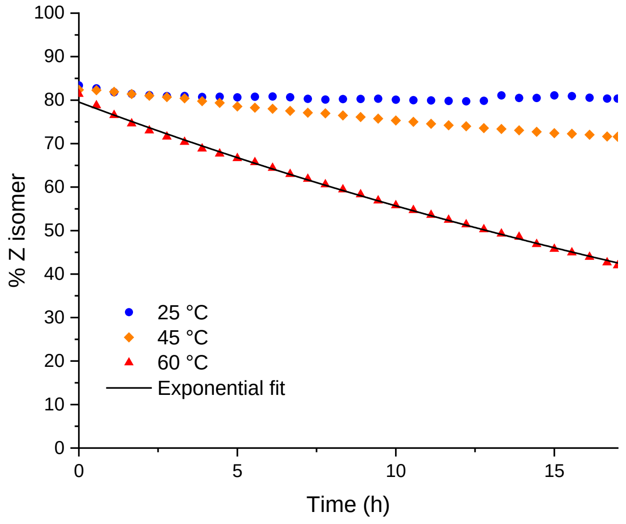

Figure S60. *Thermal relaxation of* $\mathbf{1}^{Z}$ *to* $\mathbf{1}^{E}$ *in the lipid bilayer of POPC LUVs was monitored over time at three different temperatures. Monitoring the absorbance wavelength at 330 nm allowed for the determination of % Z-isomer in the bilayer. The black line represents the first order exponential decay fitted to the 60 °C data (half-life = 24 hours).*

5 ^{1}H NMR Titration Experiments

Aliquots of anion ([TBACl] = 50 mM in acetone-d_6) were added to an acetone-d_6 solution of the receptor ([Host] = 1 mM). Spectra were recorded at 0, 0.2, 0.4, 0.6, 0.8, 1.0, 1.2, 1.4, 1.6, 1.8, 2.0, 2.5, 3.0, 4.0, 5.0, 7.0 and 10 equivalents. Acetone solvent was used to mimic the low polarity environment of the membrane interior, whilst ensuing good solubility of host and guest. Chemical shifts were referenced to the residual solvent peak.

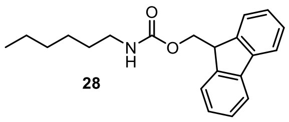

28

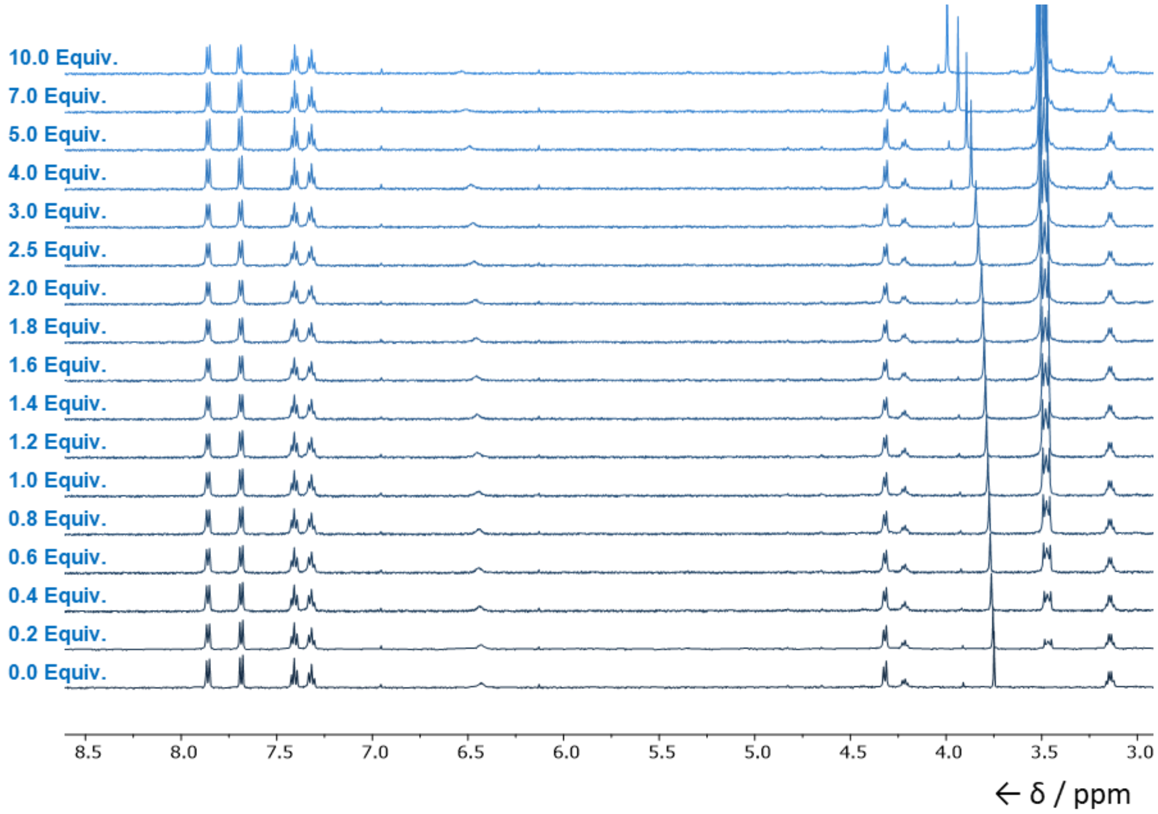

Figure S61. *Stacked partial ^{1}H NMR of hexyl Fmoc derivative* ***28*** *(1 mM, acetone-d_6, 500 MHz, 298 K) titrated with TBACl (no binding observed in this solvent).*

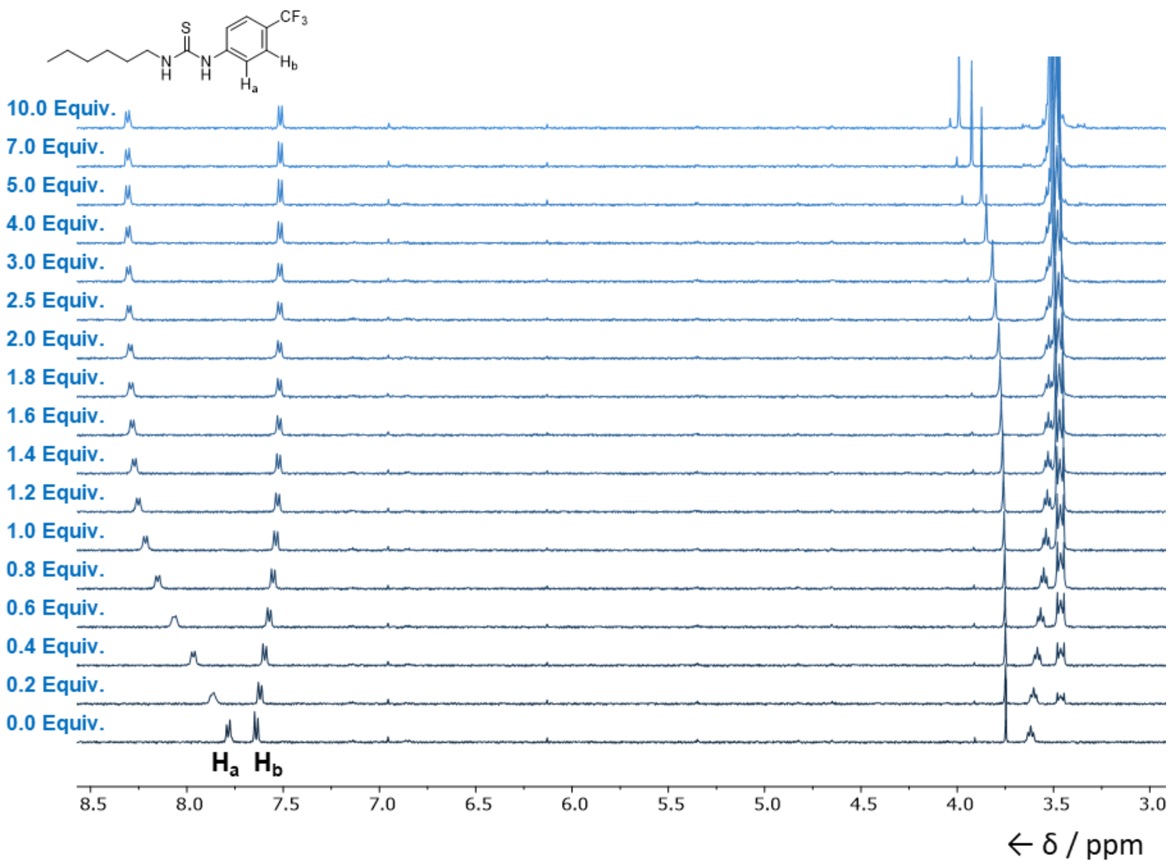

Figure S62. *Stacked partial* ^{1}*H NMR of hexyl thiourea* ***29*** *(1 mM, acetone-d*$_6$*, 500 MHz, 298 K) titrated with TBACl.*

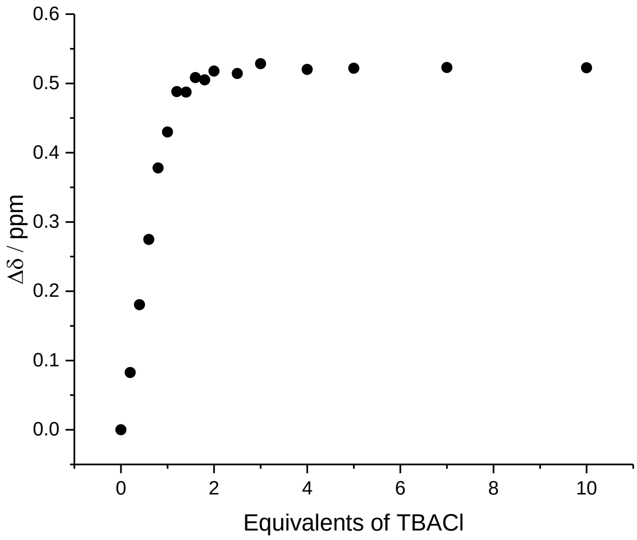

Figure S63. *Binding isotherm for* ^{1}*H NMR TBACl titrations with* ***29*** *(acetone-d*$_6$*), where circles represent experimental chemical shift perturbation of proton* H_a *upon chloride binding. Binding constant too high to be determined (>*10^4 M^{-1}*).*

6 Anion Transport Experiments

6.1 Vesicle Preparation

A thin film of lipid and transporter in various ratios was formed by evaporating a chloroform solution under reduced pressure on a rotary evaporator (25 °C) and then under high vacuum for 6 hours. The lipid film was hydrated by vortexing with the prepared buffer (100 mM NaCl, 10 mM HEPES, 1 mM 8-hydroxypyrene-1,3,6-trisulfonic acid trisodium salt (HPTS), pH 7.0). The lipid suspension was then subjected to 5 freeze-thaw cycles using liquid nitrogen and a water bath (40°C) followed by extrusion 19 times through a polycarbonate membrane (pore size 200 nm). Extrusion was performed at 50°C in the case of DPPC lipids. Extra-vesicular components were removed by size exclusion chromatography on a Sephadex G-25 column eluted with 100 mM NaCl, 10 mM HEPES, pH 7.0. Final conditions: LUVs (0.625 mM lipid); inside 100 mM NaCl, 10 mM HEPES, 1 mM HPTS, pH 7.0; outside: 100 mM NaCl, 10 mM HEPES, pH 7.0. Vesicles for the sodium gluconate, sodium bromide and sodium iodide assay were prepared by the same procedure, substituting NaCl for NaGluconate/NaBr/NaI respectively in the buffer solution.

Samples of vesicles in the cuvette containing pre-incorporated and externally added relay transporter were subjected to 2 minutes of photo-irradiation with 530 or 405 nm light from LEDs (as described above) in order to generate the PSS distribution in the membrane.

6.2 Transport assays with HPTS

In a typical experiment, the LUVs containing HPTS (100 μL, final lipid concentration 31.3 μM) were added to buffer (1880 μL of 100 mM NaCl, 10 mM HEPES, pH 7.0) at 25°C under gentle stirring. The cuvette was irradiated with 405 nm / 530 nm light for 2 minutes to generate the photo-stationary state, immediately before a pulse of NaOH (20 μL of 0.5 M solution, final concentration 5 mM) was added to initiate the experiment. After 200 s, detergent (25 μL of Triton X-100 in 7:1 (v/v) H_2O-DMSO) was added to lyse the vesicles and calibrate the assay. The fluorescence emission was monitored at λ_{em} = 510 nm (λ_{ex} = 405/460 nm). The fractional fluorescence intensity (I_{rel}) was calculated from Equation S1, where R_t is the fluorescence ratio at time *t*, (ratio of intensities from 460 nm / 405 nm excitation) R_0 is the fluorescence ratio at time 0 immediately after the base pulse, and R_d is the fluorescence ratio at time 350 s, after the addition of detergent. For each compound as the *E* and *Z* isomer, each individual concentration was repeated at-least three times and averaged; error bars represent standard deviations.

$$I_{rel} = \frac{R_t - R_0}{R_d - R_0} \qquad \textbf{(S1)}$$

The k_{ini} values were calculated by fitting the transport kinetics curves (I_{rel} vs *t*) with the exponential function I_{rel} = a – exp(-*k*t/b) using Origin 17. k_{ini} (the initial rate of transport at *t* = 0) is then given by differentiation as k_{ini} = 1/b (s^{-1}).

Experiments with DPPC lipids were conducted in the same way. For elevated temperature studies, the buffer was equilibrated at 45°C for 5 minutes prior to initiating the experiment, after the cuvette had been irradiated with 405 nm / 530 nm light for 2 minutes to generate the photo-stationary state.

Experiments in the presence of protonophore trifluoromethoxy carbonylcyanide phenylhydrazone (FCCP) were carried out using the above procedure, except that a DMSO solution of FCCP (5 µL of 100 µM solution, final concentration 0.25 µM/0.8 mol%) was added to the vesicle suspension prior to the addition of the NaOH pulse, but after the irradiation step. At this concentration, FCCP does not cause appreciable dissipation of the transmembrane pH gradient alone.

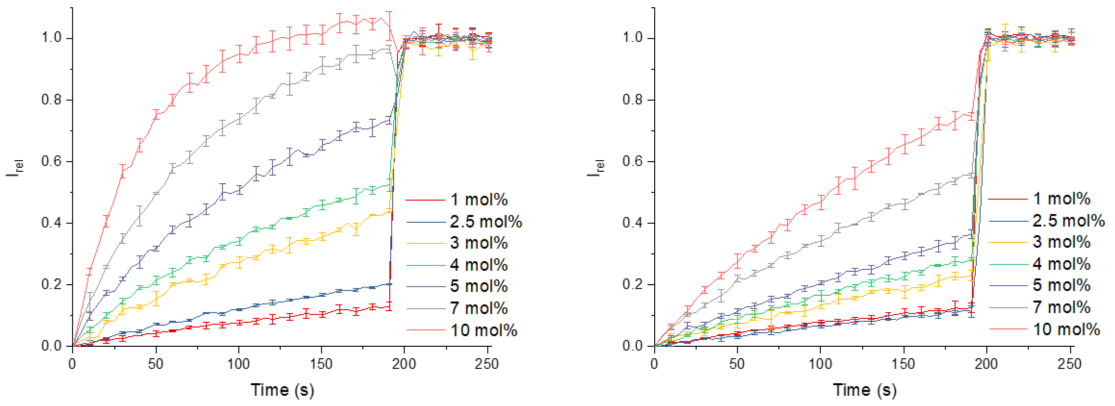

__Figure S64.__ Ion transport HPTS assay data for pre-incorporated $\mathbf{1}^E$ *(left, 1–10 mol% to lipid, PSS_{405} = 95:5 E:Z) and for pre-incorporated* $\mathbf{1}^Z$ *(right, 1–10 mol% to lipid, PSS_{530} = 15:85 E:Z) in POPC LUVs. Data shows change in ratiometric emission, I_{rel} (λ_{em} = 510 nm; λ_{ex1} = 405 nm, λ_{ex2} = 460 nm) upon addition of a NaOH base pulse (5 mM) to POPC LUVs (31.3 µM) containing 1 mM HPTS, 100 mM internal and external NaCl, buffered with 10 mM HEPES at pH 7.0. The relay transporter is pre-incorporated during vesicle preparation, and irradiated with 405 / 530 nm LEDs for 2 minutes in the cuvette to generate the photo-stationary state before addition of the base pulse.*

Hill analysis of the dose response curve of the fraction fluorescence intensities immediately prior to lysis, *y*, vs concentration of $\mathbf{1}^E$ in the membrane, afforded equivalent results to the initial rates kinetic analysis: n ~2 is indicative of two relay transporters with the anion in the rate limiting process.

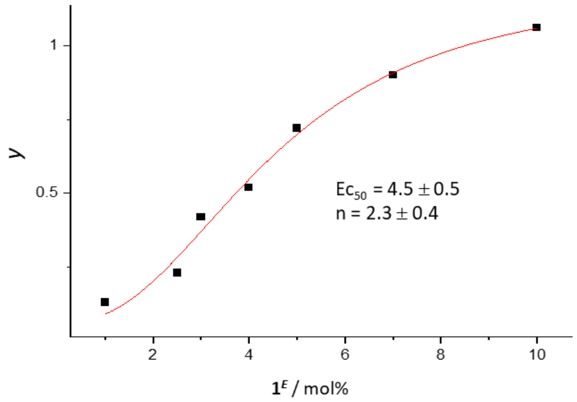

__Figure S65.__ Dependence on I_{rel} immediately prior to lysis on concentration of pre-incorporated $\mathbf{1}^E$ *(■), and fit to the Hill equation (solid line) (1–10 mol% to lipid, PSS_{405} = 95: 5 E:Z) in POPC LUVs.*

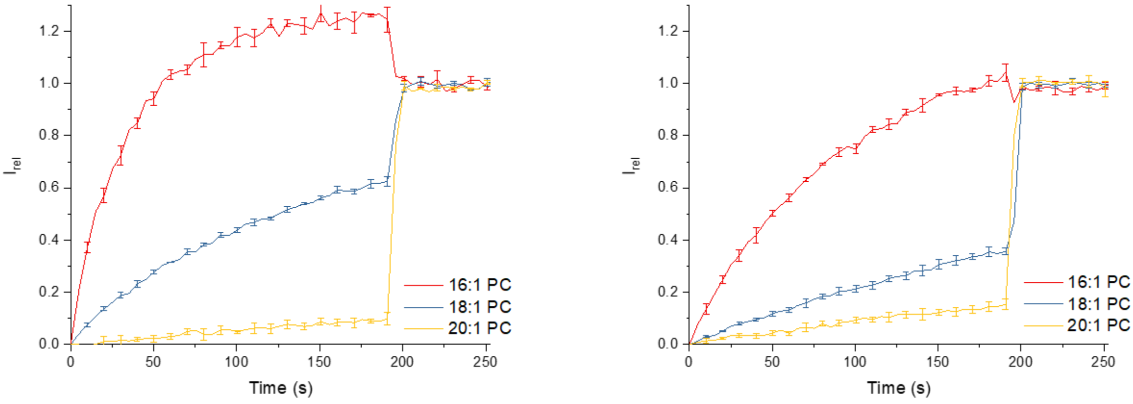

Figure S66. *Ion transport HPTS assay data for pre-incorporated $\mathbf{1}^E$ (left, 5 mol% to lipid, PSS_{405} = 95:5 E:Z) and $\mathbf{1}^Z$ (right, 5 mol% to lipid, PSS_{530} = 15:85 E:Z) in LUVs prepared from different lipids.*

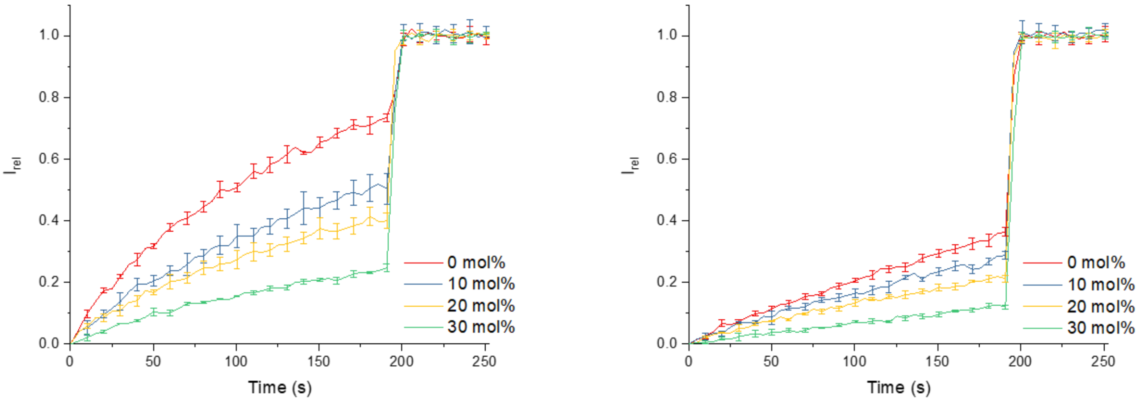

Figure S67. *Ion transport HPTS assay data for pre-incorporated $\mathbf{1}^E$ (left, 5 mol% to lipid, PSS_{405} = 95:5 E:Z) and $\mathbf{1}^Z$ (right, 5 mol% to lipid, PSS_{530} = 15:85 E:Z) in POPC LUVs with varying concentrations of cholesterol (mol%).*

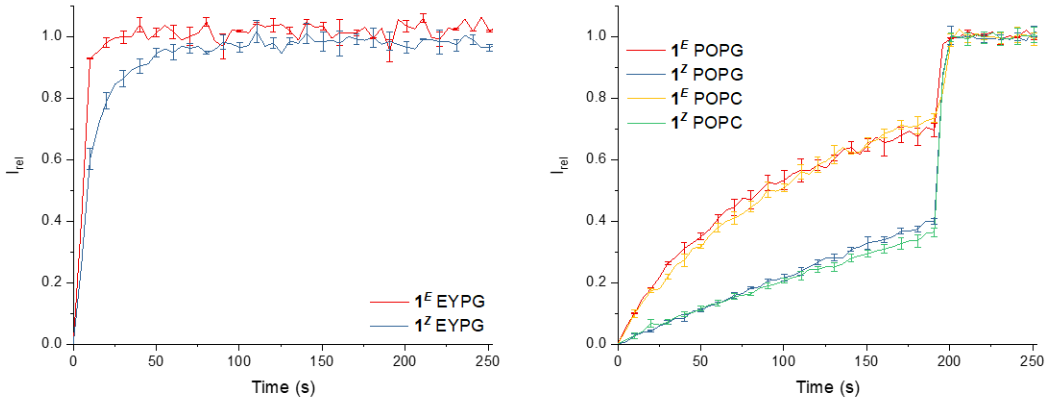

Figure S68. *Ion transport HPTS assay data for pre-incorporated $\mathbf{1}^E$ (5 mol% to lipid, PSS_{405} = 95:5 E:Z) and $\mathbf{1}^Z$ (5 mol% to lipid, PSS_{530} = 15:85 E:Z) in EYPG LUVs (left) and POPG LUVs compared to POPC LUVs (right).*

Note that EYPG is a mixture of POPG and shorter lipids, which results in faster observed transport rates (left) that pure POPG (right)

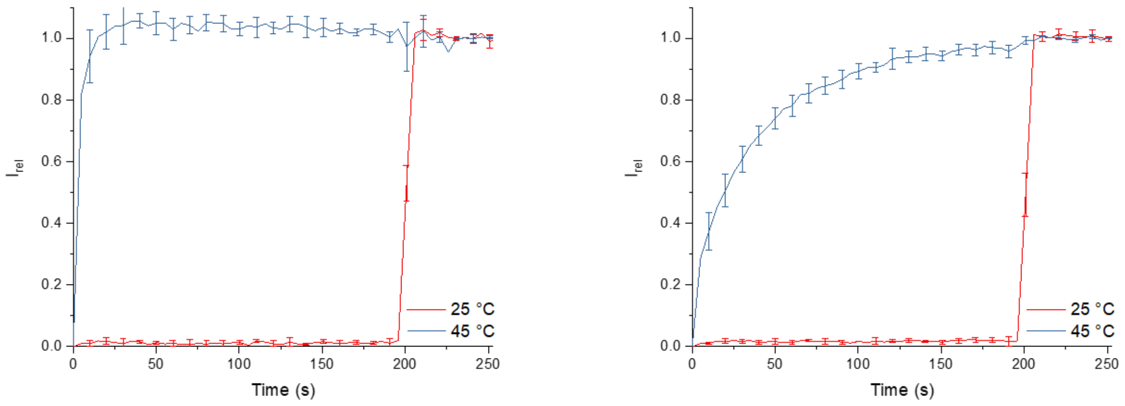

Figure S69. *Ion transport HPTS assay data for pre-incorporated* ***1***^{E} *(left, 5 mol% to lipid, PSS*$_{405}$ *= 95:5 E:Z) and* ***1***^{Z} *(right, 5 mol% to lipid, PSS*$_{530}$ *= 15:85 E:Z) in DPPC LUVs at 25 °C and 45 °C.*

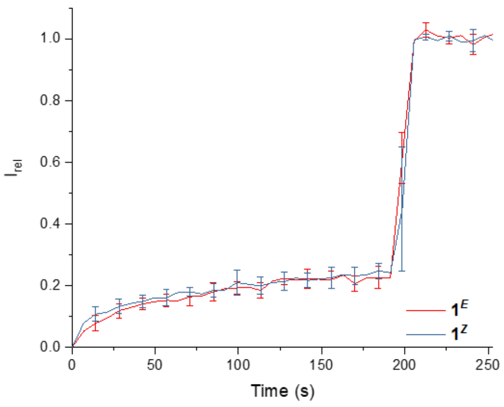

Figure S70. *Ion transport HPTS assay data for pre-incorporated* ***1***^{E} *(5 mol% to lipid, PSS*$_{405}$ *= 95:5 E:Z) and* ***1***^{Z} *(5 mol% to lipid, PSS*$_{530}$ *= 15:85 E:Z) in POPC LUVs with NaGluconate buffer.*

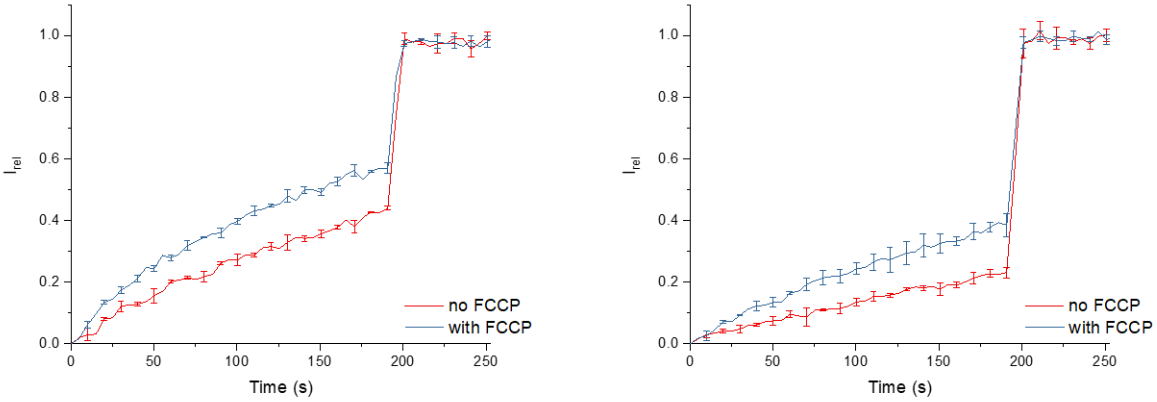

Figure S71. *Ion transport HPTS assay data for pre-incorporated* $\mathbf{1}^{E}$ *(left, 3 mol% to lipid,* PSS_{405} *= 95:5 E:Z) and* $\mathbf{1}^{Z}$ *(right, 3 mol% to lipid,* PSS_{530} *= 15:85 E:Z) in POPC LUVs with 10 mM NaCl in 100 mM HEPES buffer in the presence and absence of FCCP (5 µL of 100 µM solution in DMSO) added prior to start of the experiment.*

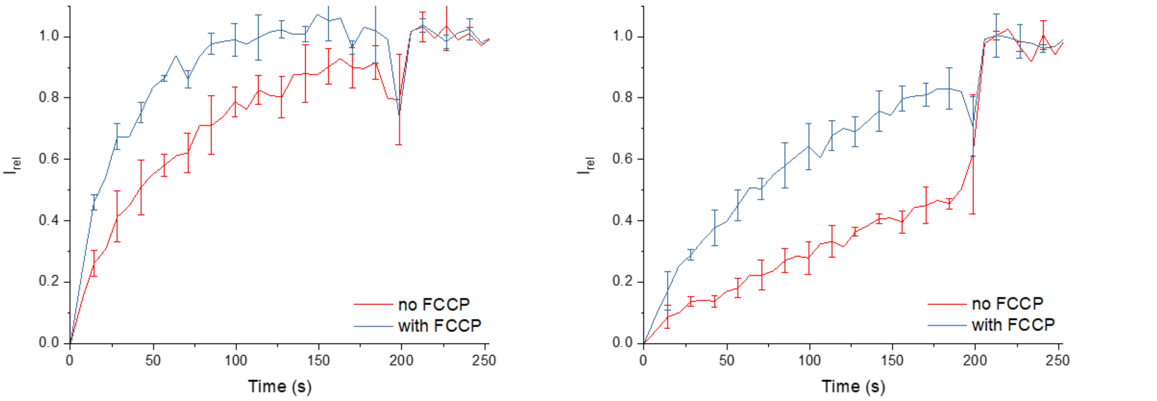

Figure S72. *Ion transport HPTS assay data for pre-incorporated* $\mathbf{1}^{E}$ *(left, 5 mol% to lipid,* PSS_{405} *= 95:5 E:Z) and* $\mathbf{1}^{Z}$ *(right, 5 mol% to lipid,* PSS_{530} *= 15:85 E:Z) in POPC LUVs with 10 mM NaBr in 100 mM HEPES buffer in the presence and absence of FCCP (5 µL of 100 µM solution in DMSO) added prior to start of the experiment.*

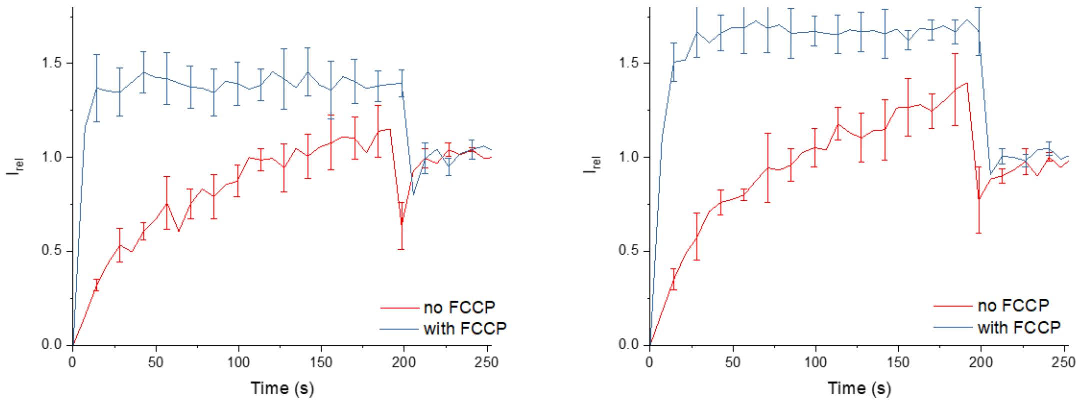

Figure S73. *Ion transport HPTS assay data for pre-incorporated* ***1***^{E} *(left, 5 mol% to lipid, PSS*$_{405}$ *= 95:5 E:Z) and* ***1***^{Z} *(right, 5 mol% to lipid, PSS*$_{530}$ *= 15:85 E:Z) in POPC LUVs with 10 mM NaI in 100 mM HEPES buffer in the presence and absence of FCCP (5 µL of 100 µM solution in DMSO) prior to start of the experiment.*

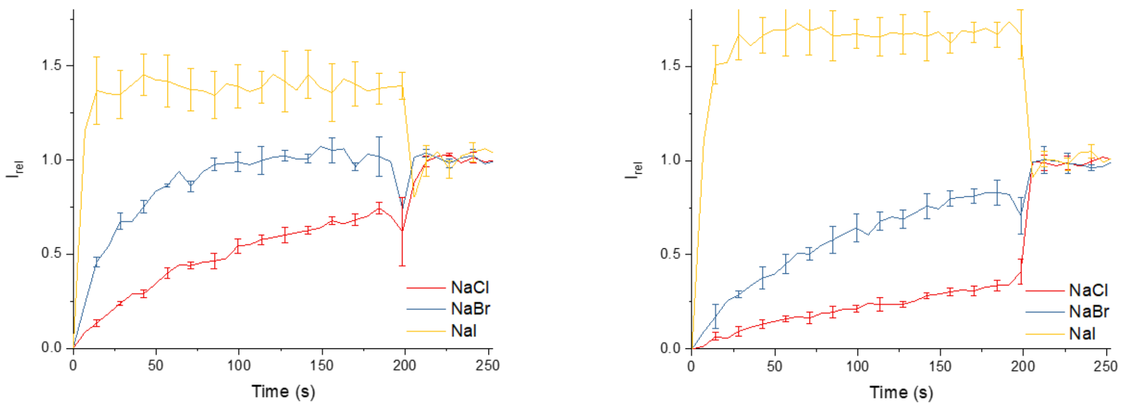

Figure S74. *Ion transport HPTS assay data for pre-incorporated* ***1***^{E} *(left, 5 mol% to lipid, PSS*$_{405}$ *= 95:5 E:Z) and* ***1***^{Z} *(right, 5 mol% to lipid, PSS*$_{530}$ *= 15:85 E:Z) in POPC LUVs with 10 mM NaX in 100 mM HEPES buffer in the presence of FCCP (5 µL of 100 µM solution in DMSO) added prior to the start of the experiment (X = Cl, Br, I).*

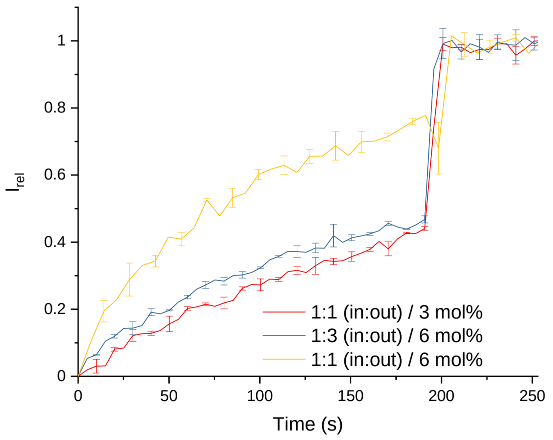

Figure S75. *Ion transport HPTS assay data for pre-incorporated **1** (3 mol% to lipid) with and without the external addition of **1** (5 µL of 375 µM DMSO stock solution) prior to the irradiation of the cuvette with 405 nm light for 2 minutes (**1**^{E} PSS$_{405}$ = 95:5 E:Z) in POPC LUVs compared to transport of pre-incorporated **1**^{E} (6 mol% to lipid, PSS$_{405}$ = 95:5 E:Z).*

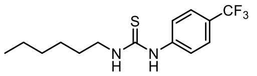

29

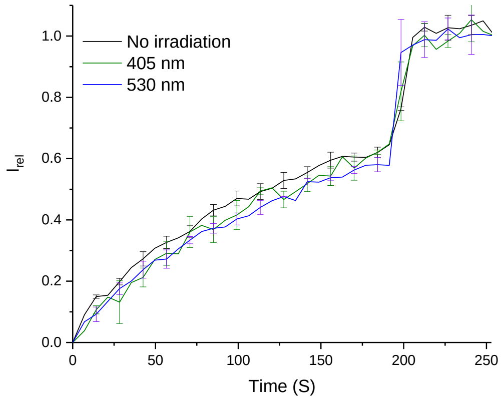

Figure S76. *Ion transport HPTS assay data for thiourea mobile carrier **29** (100 nM, 0.32 mol% to lipid) in POPC LUVs when externally added (5 µL of DMSO stock solution) and then irradiated with light (405 nm / 530 nm) for 5 minutes.*

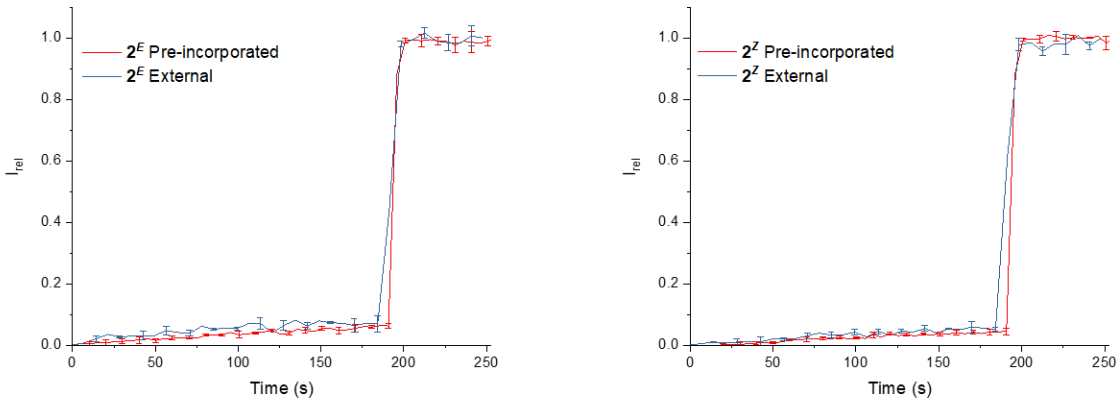

Figure S77. *Ion transport HPTS assay data for* $\mathbf{2^E}$ *(left, 5 mol% to lipid, PSS*$_{405}$ *= 95:5 E:Z) and* $\mathbf{2^Z}$ *(right, 5 mol% to lipid, PSS*$_{530}$ *= 16:84 E:Z) in POPC LUVs when pre-incorporated and externally added (5 µL of DMSO stock solution) and irradiated in the bilayer for 2 minutes using 530 nm or 405 nm LEDS to reach the photo-stationary state in the HPTS assay.*

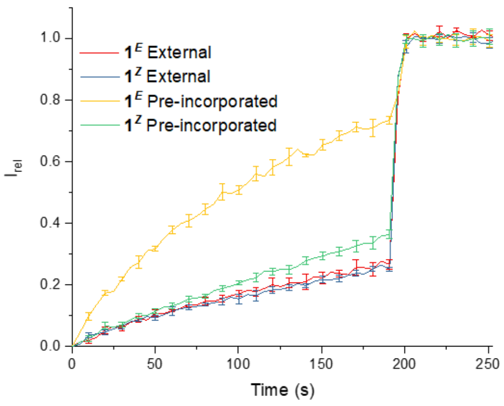

Figure S78. *Ion transport HPTS assay data for* $\mathbf{1^E}$ *(5 mol% to lipid, PSS*$_{405}$ *= 95:5 E:Z) and* $\mathbf{1^Z}$ *(5 mol% to lipid, PSS*$_{530}$ *= 15:85 E:Z) in POPC LUVs when pre-incorporated and externally added (5 µL of DMSO stock solution) and irradiated in the bilayer for 2 minutes using 530 nm or 405 nm LEDS to reach the photo-stationary state in the HPTS assay.*

Note that external addition of both $\mathbf{1^E}$ and $\mathbf{1^Z}$ led to minimal, yet identical background activity, (presumably due to a small increase in membrane ion permeability following external addition of transporter) and demonstrates that transporters in the outer leaflet alone are incapable of relay transport. In contrast, pre-incorporation of transporters in both leaflets facilitates faster transport for $\mathbf{1^E}$ *via* a transmembrane relay mechanism.

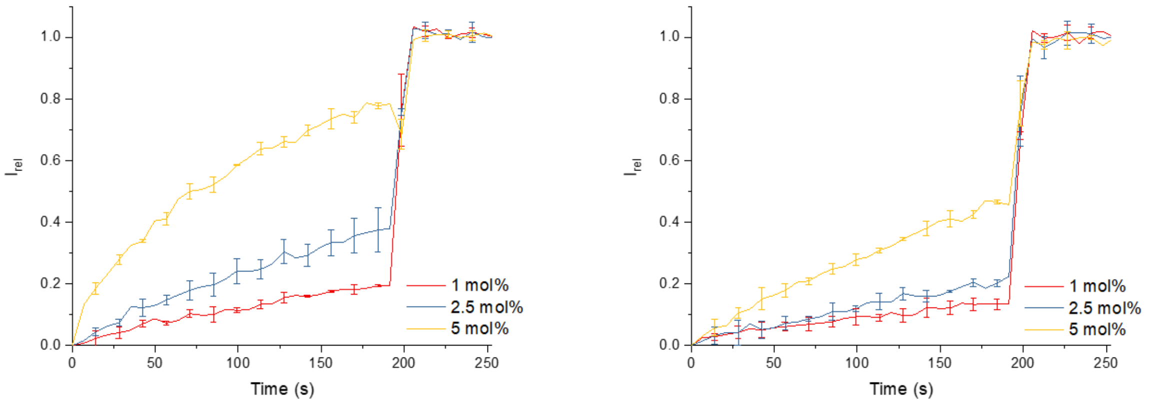

Figure S79. *Ion transport HPTS assay data for pre-incorporated* $\mathbf{3}^{E}$ *(left, 1-5 mol% to lipid,* PSS_{405} *= 96:4 E:Z) and* $\mathbf{3}^{Z}$ *(right, 1-5 mol% to lipid,* PSS_{530} *= 10:90 E:Z) in POPC LUVs.*

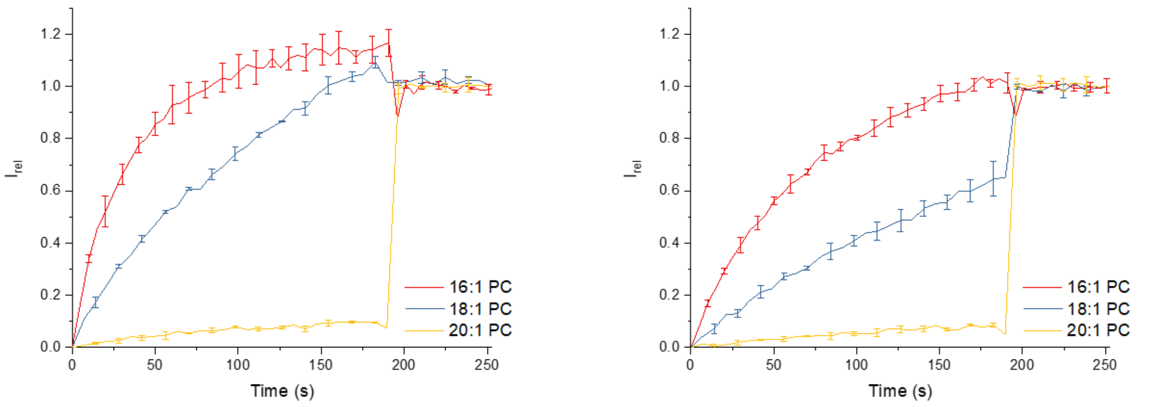

Figure S80. *Ion transport HPTS assay data for pre-incorporated* $\mathbf{3}^{E}$ *(left, 5 mol% to lipid,* PSS_{405} *= 96:4 E:Z) and* $\mathbf{3}^{Z}$ *(right, 5 mol% to lipid,* PSS_{530} *= 10:90 E:Z) in LUVs prepared from different lipids.*

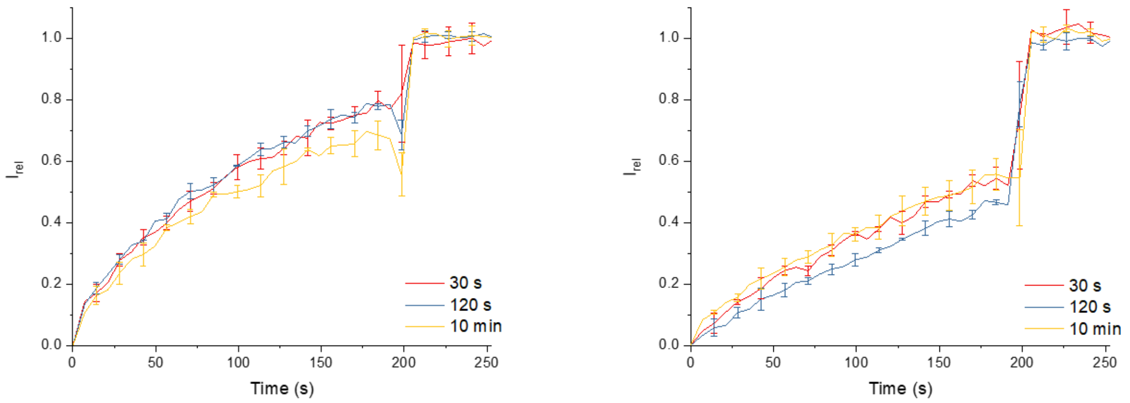

Figure S81. *Ion transport HPTS assay data for pre-incorporated* $\mathbf{3}^{E}$ *(left, 5 mol% to lipid,) and* $\mathbf{3}^{Z}$ *(right, 5 mol% to lipid,) in POPC LUVs with varying lengths of pre-irradiation with 405 nm (left data) or 530 nm (right data) LEDS.*

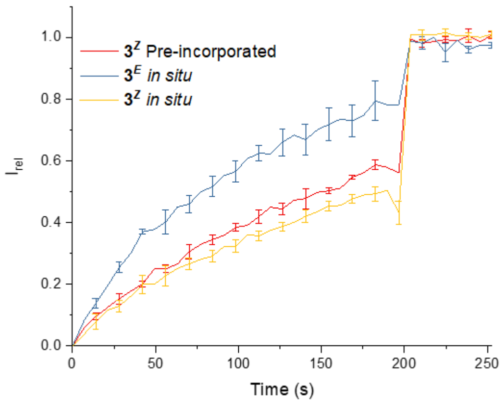

Figure S82. *Ion transport HPTS assay data for* $\mathbf{3}^{E}$ *(5 mol% to lipid,* PSS_{405} *= 96:4 E:Z) and* $\mathbf{3}^{Z}$ *(5 mol% to lipid,* PSS_{530} *= 10:90 E:Z) in POPC LUVs. Compound* $\mathbf{3}^{Z}$ *was pre-irradiated prior to LUV preparation and light was excluded during their preparation to determine transport activity of pre-incorporated* $\mathbf{3}^{Z}$ *(5 mol% to lipid,* PSS_{530} *= 96:4 E:Z). The vesicle suspension was also pre-irradiated with 405 nm and 530 nm light prior to the HPTS assay to obtain the in-situ irradiated data.*

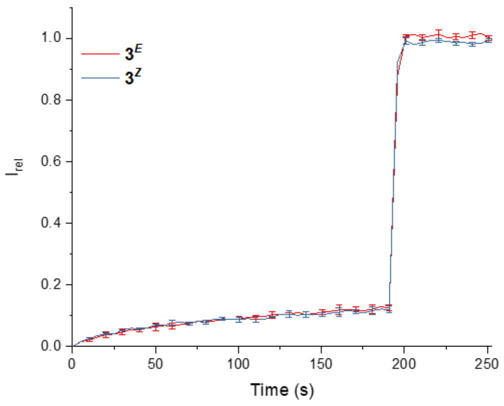

Figure S83. *Ion transport HPTS assay data for pre-incorporated **3^E** (5 mol% to lipid, PSS_{405} = 96:4 E:Z) and **3^Z** (5 mol% to lipid, PSS_{530} = 10:90 E:Z) in POPC LUVs with NaGluconate buffer.*

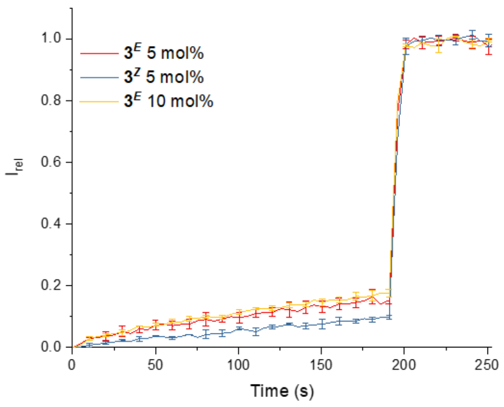

Figure S84. *Ion transport HPTS assay data for **3^E** (5 mol% to lipid, PSS_{405} = 96:4 E:Z) and **3^Z** (5 and 10 mol% to lipid, PSS_{530} = 10:90 E:Z) in POPC LUVs when externally added (5 µL of DMSO stock solution) and then irradiated in the bilayer.*

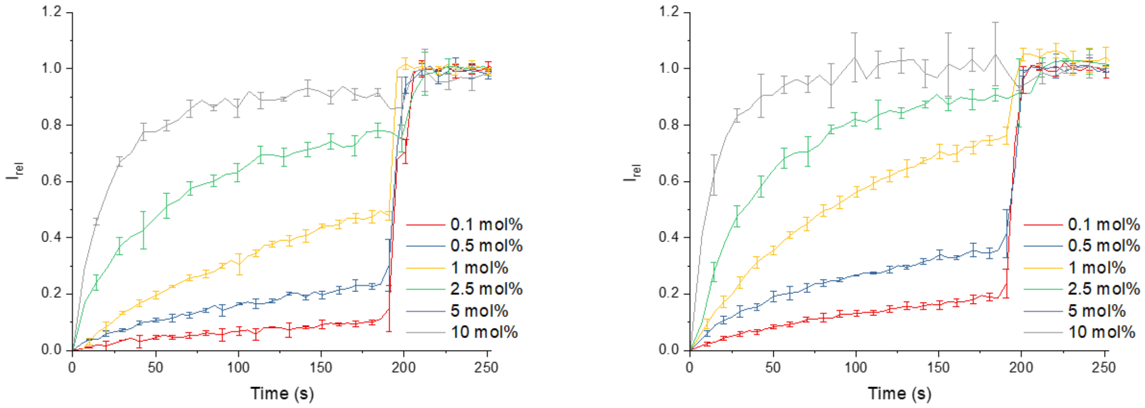

Figure S85. *Ion transport HPTS assay data for pre-incorporated* $\mathbf{4}^{E}$ *(left, 0.1-10 mol% to lipid,* PSS_{405} *= 96:4 E:Z) and* $\mathbf{4}^{Z}$ *(right, 0.1-10 mol% to lipid,* PSS_{530} *= 10:90 E:Z) in POPC LUVs.*

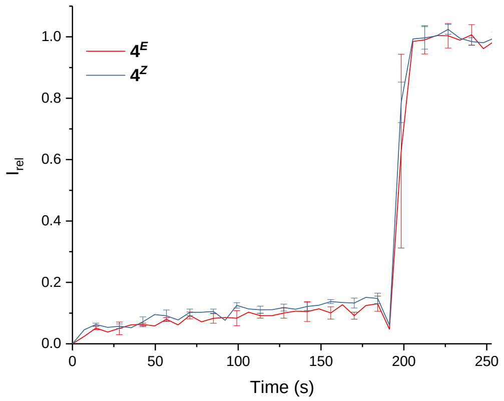

Figure S86. *Ion transport HPTS assay data for pre-incorporated* $\mathbf{4}^{E}$ *(5 mol% to lipid,* PSS_{405} *= 96:4 E:Z) and* $\mathbf{4}^{Z}$ *(5 mol% to lipid,* PSS_{530} *= 10:90 E:Z) in POPC LUVs with NaGluconate buffer.*

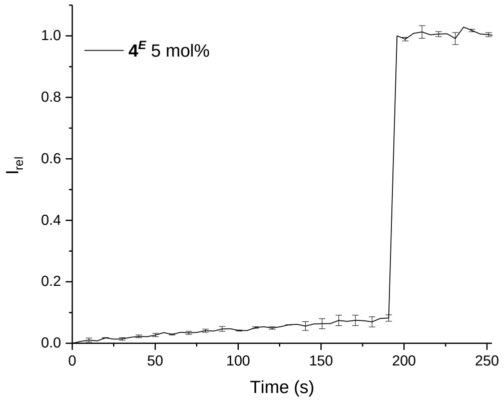

Figure S87. *Ion transport HPTS assay data for* $\mathbf{4}^{E}$ *(5 mol% to lipid,* PSS_{405} *= 96:4 E:Z) in POPC LUVs when externally added as a pre-irradiated solution (5 µL of DMSO stock solution).*

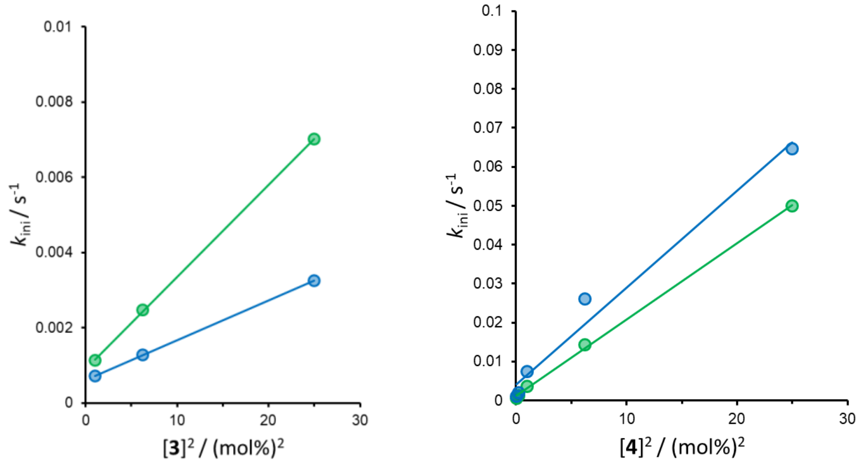

Figure S88. *Dependence of k_{ini} on [**3**]^2 (left) and [**4**]^2 (right) for the E-(green data) and Z-rich (blue data) PSS generated by in-situ photo-isomerization.*

Table S1. *Initial rates of transport for the E- and Z-rich PSS generated by in-situ photo-isomerization of compounds **1**, **3** and **4** when embedded at 5 mol% in POPC LUVs, and in LUVs of various lipid bilayer compositions.*

		POPC	16:1 PC	18:1 PC	20:1 PC	POPC : 10% Cholesterol	POPC : 20% Cholesterol	POPC : 30% Cholesterol
**1**	$k_{ini(E)}$ / 10^{-3} s^{-1}	6.6 (0.3)	25.5 (0.2)	5.0 (0.1)	0.57 (0.08)	3.7 (0.4)	2.6 (0.2)	1.41 (0.09)
	$k_{ini(Z)}$ / 10^{-3} s^{-1}	2.2 (0.1)	13.4 (0.2)	2.3 (0.2)	0.8 (0.1)	1.7 (0.1)	1.23 (0.05)	0.69 (0.06)
	$F_{(E/Z)}$	3.0 (0.1)	1.9 (0.05)	2.1 (0.2)	0.7 (0.2)	2.2 (0.1)	2.1 (0.1)	2.1 (0.1)
**3**	$k_{ini(E)}$ / 10^{-3} s^{-1}	7.0 (0.4)	25.5 (0.9)	12.3 (0.2)	0.52 (0.02)			
	$k_{ini(Z)}$ / 10^{-3} s^{-1}	3.3 (0.2)	14.4 (0.7)	5.0 (1)	0.47 (0.01)			
	$F_{(E/Z)}$	2.2 (0.1)	1.8 (0.1)	2.3 (0.4)	1.1 (0.05)			
**4**	$k_{ini(E)}$ / 10^{-3} s^{-1}	52 (2)						
	$k_{ini(Z)}$ / 10^{-3} s^{-1}	65 (8)						
	$F_{(E/Z)}$	0.8 (0.2)						

$F_{(E/Z)}$ = factor of enhancement of $k_{ini,E}/k_{ini,Z}$ (ratio of rate constants for the *E*-rich PSS over the *Z*-rich PSS). Errors at the 95% confidence limit.

7 References

1 A. Kerckhoffs, Z. Bo, S. E. Penty, F. Duarte and M. J. Langton, *Org. Biomol. Chem.*, 2021, **19**, 9058–9067.

2 L. Albert, A. Peñalver, N. Djokovic, L. Werel, M. Hoffarth, D. Ruzic, J. Xu, L.-O. Essen, K. Nikolic, Y. Dou and O. Vázquez, *ChemBioChem*, 2019, **20**, 1417–1429.

3 E. Fasoli, A. Arnone, A. Caligiuri, P. D'Arrigo, L. de Ferra and S. Servi, *Org. Biomol. Chem.*, 2006, **4**, 2974–2978.

4 A. Antoine John and Q. Lin, *J. Org. Chem.*, 2017, **82**, 9873–9876.

5 L. Zhang, H. Zhang, F. Gao, H. Peng, Y. Ruan, Y. Xu and W. Weng, *RSC Adv.*, 2015, **5**, 12007–12014.

6 L. Perlin, S. MacNeil and S. Rimmer, *Chem. Commun.*, 2008, 5951–5953.

7 S. Schultzke, M. Walther and A. Staubitz, *Molecules*, 2021, **26**, 3916.

8 R. Xu, X. Guan, M. He and J. Yang, *RSC Adv.*, 2017, **7**, 914–918.

9 S. J. Moore, M. Wenzel, M. E. Light, R. Morley, S. J. Bradberry, P. Gómez-Iglesias, V. Soto-Cerrato, R. Pérez-Tomás and P. A. Gale, *Chem. Sci.*, 2012, **3**, 2501–2509.

10 R. Rosseto and J. Hajdu, *Chemistry and Physics of Lipids*, 2014, **183**, 110–116.

11 R. Rosseto and J. Hajdu, *Tetrahedron Let.*, 2005, **46**, 2941–2944.

12 A. Kerckhoffs and M. J. Langton, *Chem. Sci.*, 2020, **11**, 6325–6331.

13 C. Knie, M. Utecht, F. Zhao, H. Kulla, S. Kovalenko, A. M. Brouwer, P. Saalfrank, S. Hecht and D. Bléger, *Chem. Eur. J.*, 2014, **20**, 16492–16501.
